# Spatial patterns of tumour growth impact clonal diversification

**DOI:** 10.1038/s41559-021-01586-x

**Published:** 2021-12-23

**Authors:** Xiao Fu, Yue Zhao, Jose I. Lopez, Andrew Rowan, Lewis Au, Annika Fendler, Steve Hazell, Hang Xu, Stuart Horswell, Scott T.C. Shepherd, Charlotte E. Spencer, Lavinia Spain, Fiona Byrne, Gordon Stamp, Tim O’Brien, David Nicol, Marcellus Augustine, Ashish Chandra, Sarah Rudman, Antonia Toncheva, Andrew J.S. Furness, Lisa Pickering, Santosh Kumar, Dow-Mu Koh, Christina Messiou, Derfel ap Dafydd, Matthew R. Orton, Simon J. Doran, James Larkin, Charles Swanton, Erik Sahai, Kevin Litchfield, Samra Turajlic, the TRACERx Renal Consortium, Lewis Au, Ben Challacombe, Ashish Chandra, Simon Chowdhury, William Drake, Annika Fendler, Archana Fernando, Nicos Fotiadis, Andrew Furness, Emine Hatipoglu, Karen Harrison-Phipps, Steve Hazell, Peter Hill, Catherine Horsfield, James Larkin, Jose I. Lopez, Teresa Marafioti, David Nicol, Tim O’Brien, Jonathon Olsburgh, Lisa Pickering, Alexander Polson, Sergio Quezada, Sarah Rudman, Scott Shepherd, Charlotte E. Spencer, Charles Swanton, Samra Turajlic, Mary Varia, Hema Verma, Paul A. Bates

**Affiliations:** 1 Biomolecular Modelling Laboratory, The Francis Crick Institute, 1 Midland Rd, London NW1 1AT, UK; 2 Tumour Cell Biology Laboratory, The Francis Crick Institute, 1 Midland Rd, London NW1 1AT, UK; 3 Cancer Evolution and Genome Instability Laboratory, The Francis Crick Institute, 1 Midland Rd, London NW1 1AT, UK; 4 Cancer Research UK Lung Cancer Centre of Excellence, University College London Cancer Institute, Paul O’Gorman Building, 72 Huntley Street, London, WC1E 6BT, UK; 5 Department of Thoracic Surgery, Fudan University Shanghai Cancer Center, Shanghai, China. 200032; 6 Department of Oncology, Shanghai Medical College, Fudan University, Shanghai, China. 200032; 7 Department of Pathology, Cruces University Hospital, Biocruces-Bizkaia Institute, 48903 Barakaldo, Bizkaia, Spain; 8 Cancer Dynamics Laboratory, The Francis Crick Institute, 1 Midland Rd, London NW1 1AT, UK; 9 Renal and Skin Units, The Royal Marsden Hospital, London, SW3 6JJ, UK; 10 Department of Pathology, the Royal Marsden NHS Foundation Trust, London SW3 6JJ, UK; 11 Stanford Cancer Institute, Stanford University School of Medicine, Stanford, California, US; 12 Department of Bioinformatics and Biostatistics, The Francis Crick Institute, 1 Midland Rd, London NW1 1AT, UK; 13 Experimental Histopathology Laboratory, The Francis Crick Institute, London NW1 1AT, UK; 14 Urology Centre, Guy’s and St. Thomas’ NHS Foundation Trust, London SE1 9RT, UK; 15 Department of Urology, the Royal Marsden NHS Foundation Trust, London SW3 6JJ, UK; 16 Department of Pathology, Guy’s and St. Thomas NHS Foundation Trust, London SE1 9RT, UK; 17 Department of Medical Oncology, Guy’s and St. Thomas’ NHS Foundation Trust, London SE1 9RT, UK; 18 Biobank, Guy’s and St. Thomas’ NHS Foundation Trust, London SE1 7EH, UK; 19 Division of Radiotherapy and Imaging, Institute of Cancer Research, 15 Cotswold Road, Sutton, Surrey, SM2 5NG; 20 Department of Radiology, Royal Marsden Hospital, Fulham Rd, London, SW3 6JJ; 21 Artificial Intelligence Imaging Hub, Royal Marsden NHS Foundation Trust, Sutton, UK; 22 Department of Medical Oncology, University College London Hospitals, 235 Euston Rd, Fitzrovia, London, NW1 2BU, UK

**Keywords:** Microdiversity, Parallel evolution, Clear cell renal cell carcinoma, Computational modelling, Tumour growth patterns, Time-course analysis

## Abstract

Genetic intra-tumour heterogeneity (ITH) fuels cancer evolution, but our understanding of temporal evolution and the ability to predict clinically relevant evolutionary trajectories remain limited. Towards enhancing this ability, we investigated features of clonal diversification in clear cell renal cell carcinomas (ccRCCs) through combined modelling and tumour analysis. Tumour growth modes impact the extent of subclonal diversification, enabling interpretation, through the lens of temporal evolution, of static data on tumour sizes and clonal diversity in the TRACERx Renal study. Patterns of proliferation and necrosis in diverse growth models underlie the spatial features of clonal diversity and recent subclone births, with proliferation at the surface linked to highly branched evolution and attenuated disease progression. *In-silico* time-course studies indicate initial increase and subsequent collapse in clonal diversity. Finally, we show that radiologically evident budding structures in early-stage ccRCCs could potentially predict future evolutionary steps.

## Introduction

The development of cancer is an evolutionary process^1,2^. Acquisition of genomic alterations including mutations and somatic copy-number alterations (SCNAs) drives the emergence of genetically heterogeneous subpopulations of cancer cells or subclones^3^, resulting in intra-tumour heterogeneity (ITH). A subset of genomic alterations (termed *drivers*) endows subclones with increased fitness. Subclones compete for resources, including physical space, and undergo expansion or extinction according to their fitness under selective pressures imposed by the tumour microenvironment (TME) or therapeutic intervention. With advances in next-generation sequencing, clonal architecture and evolutionary features have been elucidated in a variety of tumour types^4–7^. However, the ability to predict clinically relevant evolutionary trajectories remains limited.

**Figure 1:**
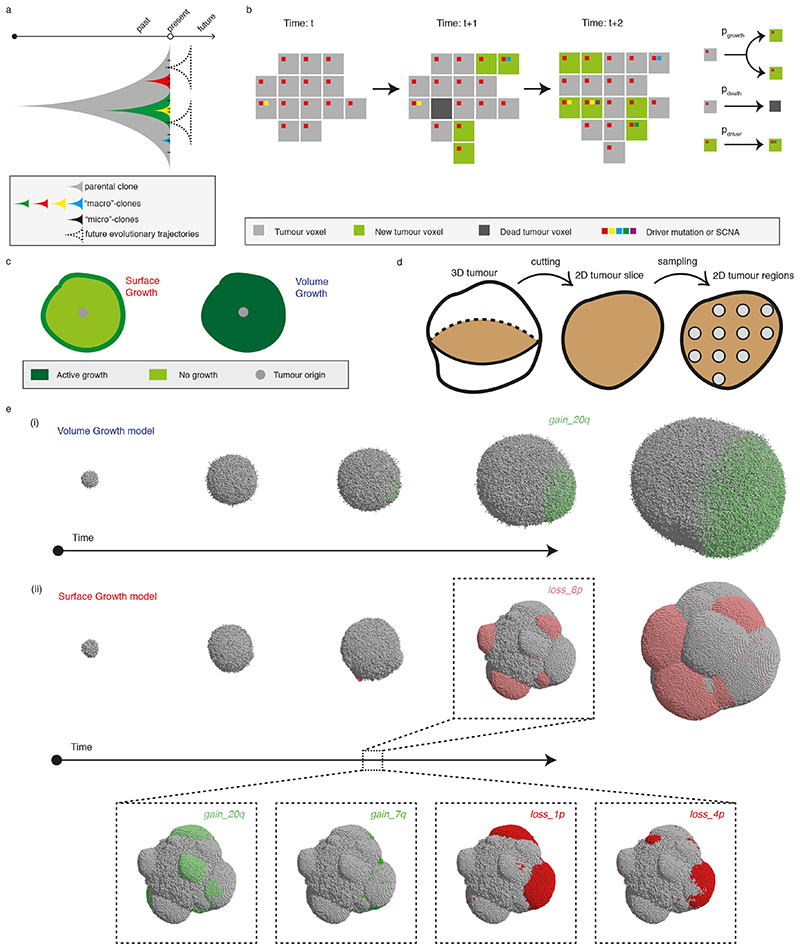
Construction of *in-silico* tumours (a) Schematic figure illustrating future evolutionary trajectories delineated by present under-detected subclones. (b) Schematic figure of probabilistic growth, death, and driver acquisition in a coarse-grained cellular automaton model. (c) Schematic figure of two growth modes: “Surface Growth” with proliferation predominating at the tumour surface and “Volume Growth” with proliferation throughout the tumour volume. (d) Schematic figure of three levels of measurements: from three-dimensional (3D) tumour to two-dimensional (2D) tumour slice and 2D tumour regions within the slice. (e) Representative *in-silico* tumours under Volume Growth (i) and Surface Growth (ii), respectively, from a 3D view. Tumour voxels harbouring select drivers, as indicated in the figure, are colour coded. Tumour voxels harbouring gain of chromosome arm events are in green; tumour voxels harbouring loss of chromosome arm events are in red. Different shades of greens or reds are employed to reflect different driver events.

One potential to enhance this ability lies in the detection and characterisation of ongoing clonal evolution. ITH provides a substrate for the selection of competent clones^8^. Detection of ITH is sampling-dependent and accurate measurement of diversity is enhanced by sampling of multiple small tumour areas^9^. While macrodiversity (i.e., the number of subclones in the whole tumour) reflects established clonal diversity within a tumour, clonal diversity at a narrow spatial scale, or microdiversity (i.e., the number of subclones within a single tumour sample), could represent under-detected ongoing clonal evolution (Figure 1a). Both macrodiversity^6,7^ and microdiversity have clinical implications. Microdiversity predicts poor survival in paediatric kidney cancer^10^ and contributes to invasion in breast tumours^11^. Clonal diversification sometimes manifests as parallel evolution, that is selection of distinct mutations in the same gene in distinct subclones^4,5,7,12,13 14,15^. More recently, parallel evolution of SCNAs was demonstrated through mirrored subclonal allelic imbalance^16^. Thus, understanding microdiversity could offer important insights into clinically relevant evolutionary potential.

Despite increasing understanding of ITH and macrodiversity, understanding of the temporal features of clonal evolution remains limited owing to ethical and logistical challenges in obtaining serial single or multi-regional biopsies from patients^17^. While longitudinal profiling of circulating tumour DNA permit clonal tracking over time^18–21^, this approach provides no resolution of spatial outgrowth and organisation of clones. To improve the understanding of spatial and temporal features of clonal diversification, we developed a coarse-grained cellular automata model of tumour growth with stochastic acquisition of driver events (Figure 1b). Various forms of non-spatial mathematical models, were formulated to describe different types of tumour growth dynamics, including exponential and polynomial growth^24,25^. More recently, modelling work incorporating spatial elements of tumour growth found an impact of growth modes on the classification of neutral evolution and selection^26–29^. Previous analysis of ccRCC supports active proliferation predominating at the tumour surface in a subset of tumours^22^ and we recently described varying rates of proliferation across the tumour^23^. In this study, we evaluated the effects of different tumour growth modes on spatial and temporal features of clonal diversification in our model, focusing on two simple growth modes: (i) uniform growth throughout the tumour volume (referred to as “Volume Growth Model”) and (ii) active proliferation restricted to the tumour surface (referred to as “Surface Growth Model”) (see Methods, Figure 1c). We additionally investigated a broader set of model conditions including the implementation of necrosis in the tumour interior, building upon our recent study^23^. We related our model to the TRAcking Cancer Evolution through therapy (Rx) (TRACERx) Renal study, which previously evaluated the genomic profiles and spatial coordinates of 756 patient tumour regions (referred to as “PT regions” hereafter) from 66 tumours^23^. Tumours with high clonal diversity don’t necessarily harbour metastasising clones^30^, suggesting that the development of metastatic competence and continuing subclonal diversification may be uncoupled in the tumour as independent evolutionary processes. Through combined modelling and clinical analysis, we show how tumour growth modes determine the extent and trajectories of clonal diversity. Crucially, we explore temporal aspects of tumour evolution that would otherwise be inaccessible from single timepoint biopsies.

## Results

### Generation of an agent-based model recapitulating ccRCC evolution

To understand the spatial and temporal features of clonal diversification, we developed a coarse-grained cellular automaton model to simulate the evolutionary dynamics of ccRCCs (see Methods for detailed description). The model includes 12 genes and 14 SCNAs (Extended Data Figure 1) identified as canonical driver events in ccRCCs in the TRACERx Renal study^7^. Each model unit, referred to as a “tumour voxel”, represents a tumour volume of 1 *mm* ^3^. Tumour voxels stochastically undergo growth, death, and acquisition of driver events upon growth. As proliferation proceeds, some tumour voxel acquires a driver event conferring selective advantages, manifested in the current study as an increase in the growth probability (*p_growth_*). Two ways of implementing selective advantages are considered and referred to as “saturated” and “additive” driver advantage models. In the saturated driver advantage model, *p_growt h_* of a tumour voxel can be at one of the three levels $\left\{p_{growt\;h\;}^{\left(initial\;\right)},p_{growt\;h\;}^{\left(moderate\;\right)},p_{growt\;h\;}^{\left(maximal\;\right)}\right\}$ . Each driver endows a tumour voxel with one of these levels, and the relative differences in selective advantage of drivers, denoted as *s*, are assumed to reflect their association with the Ki67 score in tumour regions (Extended Data Figure 1) and their frequencies in the clinical cohort^7^. For simplicity, individual driver gene mutations are assigned with $p_{growt\;h\;}^{\left(initial\right)}$ , whereas four SCNAs with strong association with Ki67 score (7q gain, 20q gain, 4q loss, and 8p loss) are assumed to be the strongest drivers assigned with $p_{growt\;h\;}^{\left(maximal\right)}$ and therefore their acquisition would lead to the biggest increase in growth probability. Importantly, the saturated model is implemented with only two levels of selective advantage, and the growth probability of a tumour voxel becomes saturated at 1 if acquiring the strongest driver. The additive driver advantage model has a more graduated implementation of selective advantage, the growth probability of a tumour voxel is defined by all the drivers it harbours, $p_{growt\;h\;}=p_{growt\;h\;}^{\left(\text{i}nitial\;\right)}+\sum_{k}p_{growt\;h\_k\;}$ , where *p_growth_k_* reflects the amount of growth probability added by driver *k* (Extended Data Figure 2). The amount *p_growth_k_* varies between drivers and is assigned according to different strengths of their association with Ki67 score (Extended Data Figures 1-2).

Additional assumptions were made to keep the model minimal. Individual driver gene mutations are assumed to be acquired with a greater probability (*p_driver_*) than SCNAs. A second mutation in the same gene is assumed to never occur in the same tumour voxel. As the majority of ccRCCs have clonal *VHL* inactivation events, in general and in the TRACERx Renal cohort^7^, the founder tumour voxel is assumed to harbour *VHL* inactivation together with 3p loss as a clonal event. Based on data from the TRACERx Renal study^7^, and functional evidence^31,32^, mutations in *PBRM1* or *BAP1*, are assumed to enhance the probability of SCNA acquisition. Mutations and acquisition of SCNAs are assumed to be proliferation-dependent, which imply DNA replication and chromosome mis-segregation as the main source of genomic alterations. Lastly, the selective advantage endowed by a driver is assumed to be fixed, so the variation in driver advantage dependent on changing environments is not considered in the current study.

Each simulation starts from a single tumour voxel carrying *VHL* and 3p loss and ends when the size exceeds 1 million tumour voxels, reflecting a tumour diameter of approximately 12 cm. Simulated tumours are analysed at multiple spatial scales (Figure 1d). A flow diagram of simulation procedure is presented in Extended Data Figure 3.

### Growth modes impact the extent of clonal diversification

**Figure 2:**
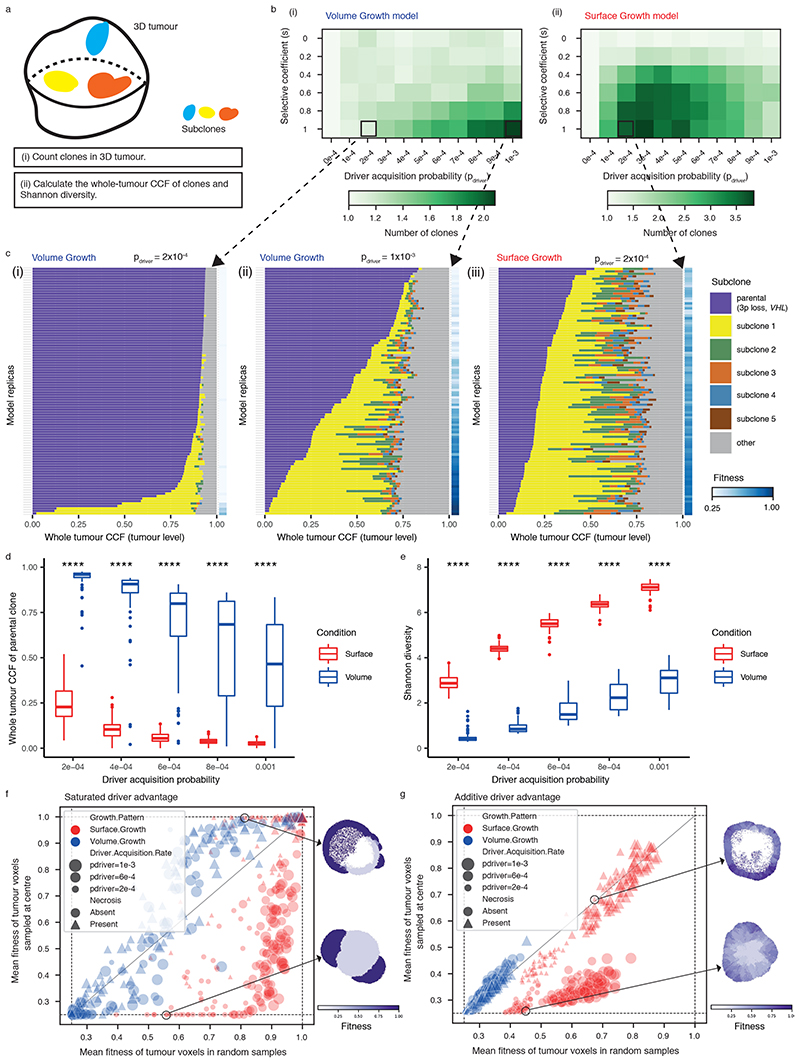
Growth modes impact the extent of clonal diversification and tumour fitness (a) Schematic figure for the whole-tumour analysis of clonal diversity. (b) Heatmap showing the average number of clones (i.e., parental clone and subclones) with respect to driver acquisition probability (*p_driver_*) and selective coefficient (s) in the Volume Growth (i) and Surface Growth (ii) models. The average is calculated from 50 *in silico* tumours per parameter condition. Clones with a whole-tumour cancer cell fraction (CCF) of at least 0.05 are counted. (c) Whole-tumour CCF of parental and largest subclones in *in-silico* tumours under Volume Growth (i-ii) and Surface Growth (iii), respectively. Average fitness in a tumour slice for each simulation is presented as a heat map. Driver acquisition probabilities in these sets of simulations are *p_driver_* = 2 × 10^−4^ in (i), 1 × 10^−3^ in (ii), 2 × 10^−4^ in (iii), respectively. “Parental (3p loss, *VHL*)” clone is shown along with up to five subclones with a whole-tumour CCF of 0.01 or higher. All remaining subclones are represented in the “other” group. (d) Whole-tumour CCF of parental clone in *in-silico* tumours under Volume Growth and Surface Growth with varying driver acquisition probabilities. N = 100 for each condition. (e) Shannon diversity index in *in-silico* tumours under Volume Growth and Surface Growth with varying driver acquisition probabilities. N = 100 for each condition. (f-g) Mean fitness of randomly sampled (10% of all) tumour voxels against the mean fitness of the central-most (10%) tumour voxels, in models with saturated (f) and additive (g) driver advantages. Data points reflect sets of simulations with varying growth patterns (colour), driver acquisition rates (size), and implementation of necrosis (symbol). Heatmaps indicating the fitness in representative *in-silico* tumours under Surface Growth without or with the implementation of necrosis are shown. Statistical annotations in (d-e) reflect two-sided Wilcoxon tests: “****” indicates *P* ≤0.0001. In box plots in (d-e), the ends of the box reflect the lower (Q1) and upper (Q3) quartiles, with the difference indicating the interquartile range (IQR); the horizontal line dividing the box reflects the median; the ends of the vertical line indicate the extreme values within the range from *Q*1 − 1.5 × *IQR* to *Q*3 + 1.5 × *IQR*; dots beyond the vertical line indicate potential outliers.

We hypothesised that the growth mode influences the extent of clonal diversification. To test this, we first assessed the clonal diversity at the end of a simulation. Tumours under Volume Growth commonly harboured only parental clone with the lack of further subclonal diversification or contained a single dominant subclone, whereas tumours under Surface Growth harboured multiple advantageous subclones (Figure 1e). Intriguingly, expanding subclones in tumours under Surface Growth were also associated with changes in tumour morphology. These subclones initially appeared as bulging structures in a localised manner and subsequently outgrew to cover large areas of the tumour surface. Next, we counted the number of clones in the whole tumour (Figure 2a-b). Under Volume Growth, subclones were only observed in tumours with larger *s* and larger *p_driver_* (Figure 2b). By contrast, under Surface Growth model, for a wide range of *s*, tumours with small to moderate *p_driver_* harboured more subclones (Figure 2b). We then illustrated the fractions of a tumour that subpopulations occupied and tumour fitness (measured as the average growth probabilities of tumour voxels in a tumour slice, see Methods). Overall, Volume Growth models depicted a dichotomous pattern of clonal evolution and fitness: limited evidence of clonal diversification with low tumour fitness (Figure 2c (i)) or presence of a single dominant clone with high tumour fitness (Figure 2c (ii)). The latter pattern reflected early fixation of a highly fit clone in a subset of “born to be bad” tumours. In comparison, in the Surface Growth model, extensive subclonal diversification and enhanced tumour fitness were evident in nearly all cases, even with a small *p_driver_* (Figure 2c (iii)). With a large *p_driver_*, almost all tumours achieved peak fitness (Extended Data Figure 1). More extensive subclonal diversification was also noted in models with additive driver advantages (Supplementary Note 1, Extended Data Figure 2). These differences between growth models were quantitatively reflected in the whole-tumour cancer cell fraction (CCF) of the parental clone (Figure 2d) and Shannon diversity index (see Methods, Figure 2e). Using these two metrics, greater extent of diversification in the Surface Growth model was also noted for conditions with still smaller *p_driver_* (Supplementary Figure 1) or smaller *s* (Supplementary Figure 2). In the interest of characterising patterns of subclonal diversification and contrasting the two growth modes, we limit our parameter analysis, to *s* = 1 and a range of *p_driver_* from 2 × 10^−4^ to 1 × 10^−3^.

We further incorporated central necrosis into a subset of models to evaluate its impact on clonal diversification^23^. In brief, where necrosis was incorporated, tumour voxels located far from the tumour surface underwent death with an elevated probability (*p_necrosis_* = 0.5) (see Methods). In both growth modes, incorporation of necrosis generally led to a greater extent of clonal evolution and higher fitness observed at the end of simulations (Extended Data Figure 4, Supplementary Figure 3). To further investigate the impact of necrosis on fitness at different parts of a tumour, we collected samples of centrally, marginally, or randomly located tumour voxels, respectively (see Methods). Surface Growth models in general achieved higher fitness than Volume Growth models, as evidenced in random samples (Figure 2f-g, Supplementary Figures 4-5). When necrosis was implemented, fitness in the tumour centre was significantly elevated in the Surface Growth models (Figure 2f-g, Supplementary Note 2, Supplementary Figures 4-5), in keeping with our recent study^23^. More broadly, surface Growth led to more extensive subclonal diversification, corresponding to highly branched tumour evolution, while Volume Growth resulted in either tumours with limited evidence of clonal diversification or tumours with early fixation of a fit subclone corresponding to punctuated evolution. Interestingly, these modes of evolution correspond to evolutionary subtypes identified in ccRCCs, including tumours with low fitness and limited subclonal diversification; extensive subclonal diversification characterised by a range of drivers, and those with early fixation of a highly fit clone resulting in rapid clonal sweep^7^.

### Growth modes impact the spatial distribution of clonal diversity

**Figure 3:**
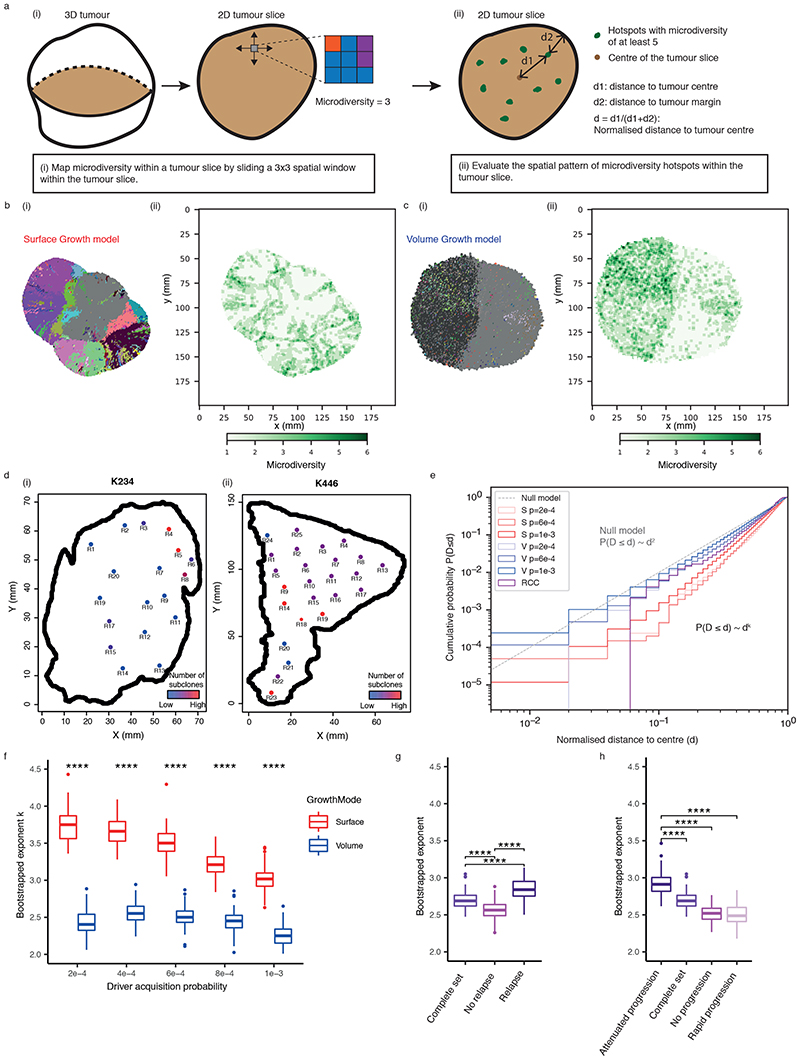
Growth modes impact the spatial features of clonal diversification (a) Schematic figure and procedure for the analysis of microdiversity within a 2D tumour slice. (b-c) Spatial maps of subclones (i) and microdiversity (ii) in a representative *in-silico* tumour under Surface Growth (b) or Volume Growth (c) with *p_driver_* = 2 × 10^−4^. Tumour voxels that belong to the parental clone are in grey, while other subclones are visualised in randomly generated colours. (d) Maps of regional biopsies with the number of subclones within a biopsy colour coded in two cases (K234 and K446) in the TRACERx Renal study. Hues from red to purple to blue reflect decreasing number of subclones. “Low” and “High” reflect 1 and 4 subclones in K234, 2 and 4 subclones in K446, respectively. (e) Cumulative probability distribution, *P*(*D* ≤ *d*), of the normalised distances to tumour centre in *in-silico* tumours under Surface Growth and Volume Growth and in ccRCC tumours. N = 100 for each model condition. “S” and “V” in the figure reflect Surface Growth and Volume Growth, respectively. “p=2e-4” reflects a driver acquisition probability of 2 × 10^−4^. 606 patient tumour (PT) regions from 54 ccRCC tumours are considered for this analysis. “Null model” reflects a power law with an exponent of 2. (f) Bootstrapped power law exponent *k*, as in *P*(*D* ≤ *d*)~*d^k^*, fitted to cumulative probability distributions generated from bootstrap samples (see Methods). N=100 *k* values per condition are presented. (g-h) Bootstrapped power law exponent *k* in ccRCC tumours partitioned according to relapse status (g) or rate of disease progression (h), respectively. N=100 *k* values per condition are presented. Statistical annotations in (f-h) reflect two-sided Wilcoxon tests: “****” indicates *P* ≤0.0001. In box plots in (f-h), the ends of the box reflect the lower (Q1) and upper (Q3) quartiles, with the difference indicating the interquartile range (IQR); the horizontal line dividing the box reflects the median; the ends of the vertical line indicate the extreme values within the range from *Q*1− 1.5 × *IQR* to *Q*3+ 1.5 × *IQR*; dots beyond the vertical line indicate potential outliers.

We next examined the spatial distribution of clonal diversity (Figure 3a, see Methods). Under Surface Growth, multiple subclones, representing macrodiversity, outgrew to occupy distinct spatially contiguous areas with hotspots of microdiversity frequent near the tumour edge (Figure 3b). In the representative Volume Growth model, a single dominant subclone was observed with more uniform distribution of microdiversity hotspots (Figure 3c). Next, we explored whether similar patterns were present in ccRCCs. Using regions that contain at least two subclones as a proxy for microdiversity hotspots in the TRACERx Renal data, we observed both spatial patterns of microdiversity corresponding to Surface Growth model (e.g., “K234”) and Volume Growth model (e.g., “K446”) (Figure 3d). Consistent between the Surface Growth model and clinical data, while microdiversity was generally high near the edge, there was variation along the edge with higher microdiversity apparently at the more bulging regions.

Intriguingly, the cumulative probability distribution with respect to the normalised distance from microdiversity hotspots to tumour centre, *d*, depicted power law scaling (Figure 3e), suggesting that the probability of observing spots with high microdiversity along the radius of a tumour could be estimated using a simple mathematical formula (i.e., *P*(*D* ≤ *d*)~*d^k^*, where *k* is the power law exponent to be fitted). Comparably, the Surface Growth model displayed a larger *k* (Figure 3f, Supplementary Figure 6), indicating a greater likelihood of microdiversity hotspots being enriched near the tumour edge. Critically, spatial homogenisation of subclone patterns abolished the characteristic scaling behaviour (Supplementary Note 3, Extended Data Figure 5), demonstrating the importance of spatial elements of tumour growth in generating microdiversity. Models with additive driver advantages showed similar distributions of microdiversity hotspots (Supplementary Note 4, Extended Data Figure 6). The incorporation of necrosis into the model re-adjusted the spatial profile in Surface Growth models, leading to the enrichment of additional microdiversity hotpsots in the necrotic tumour centre (Supplementary Note 4, Extended Data Figure 6). The power law pattern was also observed in 54 tumours with microdiversity hotspots (Figure 3e). Moreover, there was an association between the power law scaling exponent *k* with relapse status (Figure 3g) and the rate of disease progression (Figure 3h) in TRACERx Renal study (Supplementary Table 1), where tumours mapped to a poorer clinical outcome are typically associated with a steeper spatial distribution of microdiversity hotspots and enrichment towards the tumour margin (Supplementary Note 5, Supplementary Figure 7). Tumours with attenuated progression showed a steep gradient of microdiversity hotspots, consistent with Surface Growth models. Interestingly, both tumours with no progression and those with rapid progression show a shallow gradient of microdiversity hotspots, nicely corresponding to Volume Growth models with limited evidence of evolution and with early fixation of a fit clone, respectively. The observation of spatial features of clonal diversity (Supplementary Table 2) adds to our previous finding that the overall genetic diversity correlated with patient clinical outcome^7^.

### Growth modes impact the spatial patterns of parallel evolution events and youngest subclones

**Figure 4:**
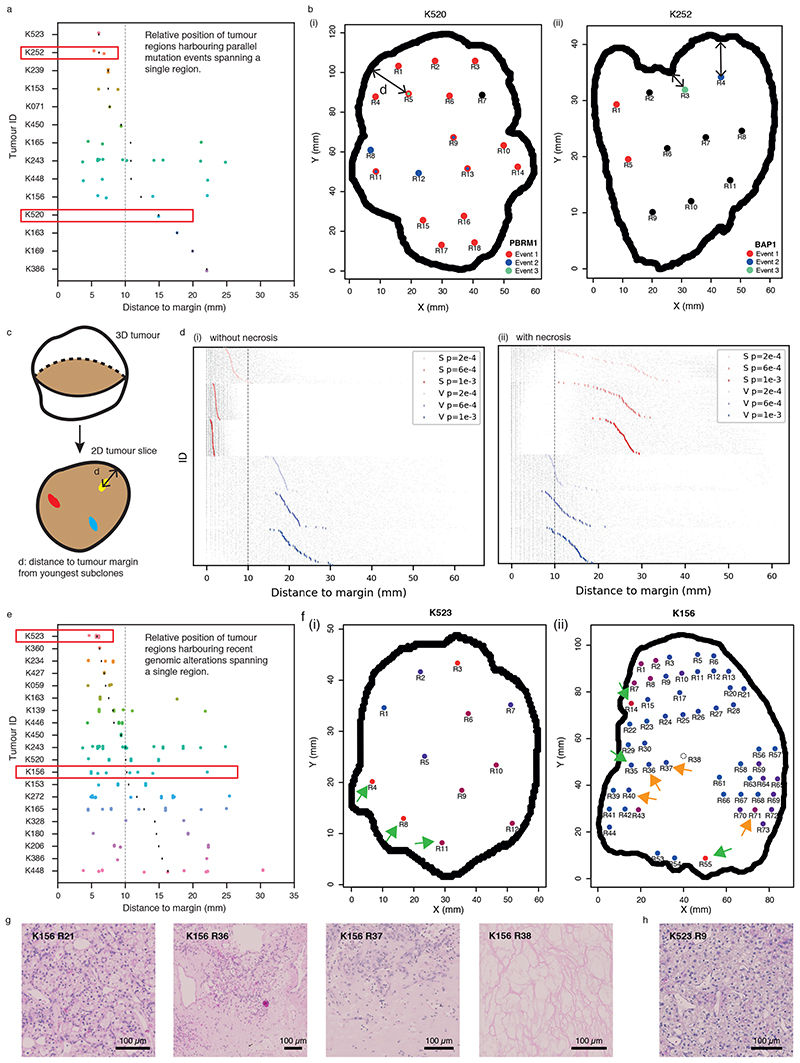
Growth modes impact the spatial features of parallel evolution and youngest subclones (a) Distance from regions harbouring parallel mutational events that span a single region to the tumour margin. Red rectangles indicate the two representative cases shown in (b). (b) Maps of regions containing parallel mutation events in two representative cases (*PBRM1* events in K520 (i) and *BAP1* events in K252 (ii)) in the TRACERx Renal study. Distinct parallel mutation events are indicated using different colours. For regions containing more than two parallel mutations, two colours are applied simultaneously. Double-headed arrow indicates the measurement of distance to tumour edge. (c) Schematic figure for the analysis of youngest subclones within a 2D tumour slice. (d) Distance from the positions of youngest subclones to the tumour margin, in models without (i) or with (ii) the implementation of necrosis. N=100 youngest subclones from each simulation are analysed and shown as grey points, with the mean distance to margin indicated with a coloured vertical bar. N=50 simulations are shown and arranged from small to large mean distance to margin (top to bottom) for each model condition. Surface Growth and Volume Growth models are shown in red and blue, respectively, with increasing driver acquisition probabilities indicated with increasing colour intensity. (e) Distance from regions harbouring genomic alterations that span a single region to the tumour margin. Only tumours with at least 10 regions are included. Red rectangles indicate the two representative cases shown in (f). (f) Maps of regional biopsies with the number of subclones within a biopsy colour coded in two representative cases (K523 and K156) in the TRACERx Renal study. Hues from red to purple to blue reflect decreasing number of subclones. “Low” and “High” reflect 1 and 4 subclones in K523, 1 and 6 subclones in K156, respectively. Regions harbouring events that span a single region are marked by arrows: in green if located within 10 mm from the tumour edge and otherwise in orange. (g-h) Histological images of representative areas from tumour regions of K156 (g) and K523 (h). Vertical dashed line in (a), (d), and (e) corresponds to a distance of 10 mm.

As subclonal diversification could involve acquisition of, and be facilitated by, distinct mutations in the same gene at spatially separate locations, we next evaluated the frequency of parallel evolution events and their spatial features. In the TRACERx Renal study, parallel evolution was observed in 28 tumours, with each event spanning a variable number of regions (Supplementary Note 6, Supplementary Table 3). Interestingly, parallel mutation events with limited clonal expansion (spanning only a single region) showed distinct spatial patterns in different ccRCCs, suggesting that ongoing convergent evolution in the same gene could operate at varying locations of a tumour (Figure 4a). In some cases (e.g., K252), such events were all close to the margin; in other cases (e.g., K520), they were located far from the edge (Figure 4a-b). We hypothesised that the observed distinct patterns of parallel mutation events could be attributed to the patterns of proliferation and accordingly recent subclone births. To test this, we returned to the computational model and examined whether and how different growth models differ in the patterns of youngest subclones (Figure 4c). Consistent with the patterns of microdiversity, we observed a preferential distribution of the youngest subclones near the tumour edge in the Surface Growth model and a more uniform distribution in the Volume Growth models (Figure 4d (i), Extended Data Figure 7). Interestingly, when necrosis was incorporated, Surface Growth models often showed a bimodal distribution with youngest subclones being born either near the tumour surface or in the necrotic interior, a pattern that was rarely observed in Volume Growth models (Figure 4d (ii)).

Motivated by the diverse patterns of youngest subclones observed in models with different growth modes, we then turned to the clinical data. Focusing on the 20 ccRCCs most extensively sampled (*n* ≥ 10 regions) cases from TRACERx Renal, we observe events that spanned a single region (assumed to be the most recent/youngest subclones) close to the tumour margin (e.g., K523, K360, and K234) and located at varying distances from the tumour margin (e.g., K156, K165, and K272) (Figure 4e-f, Extended Data Figure 7). Observations of these representative cases characterised by high levels of clonal diversity^7^ suggest that they evolved via Surface Growth but that necrosis in K156, K165 and K272 enabled birth of young subclones in the interior. Intriguingly, histological assessment of K156 showed the presence of paucicellular areas both macroscopically (Supplementary Figure 8) and microscopically (Figure 4g) in the interior of the tumour, which interfaced youngest subclones, in contrast to the interior of K523 (Figure 4h). These observations suggest that continuing proliferation and clonal evolution occur at the tumour margin but are also facilitated by available space in the interior.

### Growth modes impact the temporal features of clonal diversification

**Figure 5:**
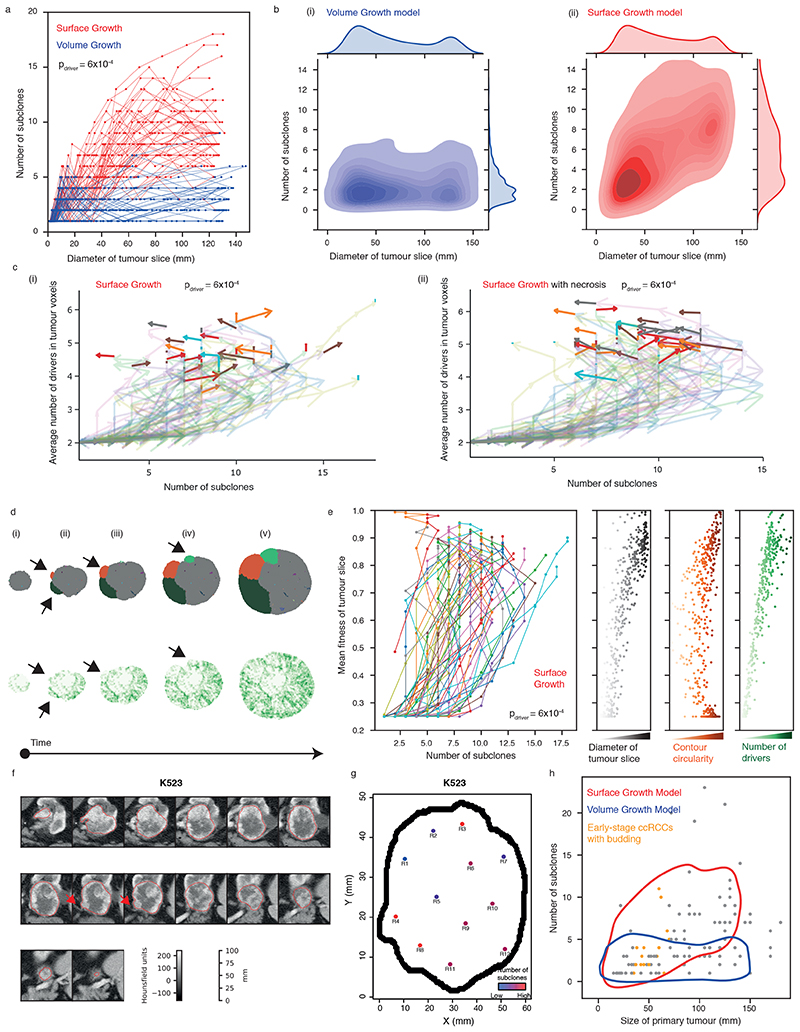
Growth modes impact the temporal features of clonal diversification (a) The number of subclones as a function of the diameter of a 2D tumour slice in *in-silico* tumours under Surface Growth and under Volume Growth, respectively. N = 50 simulations with *p_driver_* = 6 × 10^−4^ are shown for each condition. (b) Kernel density estimation (KDE) with respect to the number of subclones and the diameter of a 2D tumour slice in *in silico* tumours under Volume Growth (i) and under Surface Growth (ii). Each KDE plot is based on 250 simulations (50 per condition) under 5 conditions with *p_driver_* = 2 × 10^−4^, 4 × 10^−4^, 6 × 10^−4^, 8 × 10^−4^, 1 × 10^−3^. (c) Vector maps of evolutionary flows over time with respective to the number of subclones and the average number of drivers accumulated among tumour voxels in Surface Growth models without (i) or with (ii) the implementation of necrosis. N = 50 simulations with *p_driver_* = 6 × 10^−4^ are shown. (d) The spatial patterns of parallel mutations in *PBRM1* (upper panel, distinct events in different colours) and microdiversity (lower panel) over time in a representative *in-silico* tumour under Surface Growth. The arrows indicate budding structures preceding subclonal expansion. (e) Time evolution with respect to the number of subclones and the mean fitness of the tumour slice, along with the diameter of tumour slice, contour circularity, and average number of drivers accumulated, in Surface Growth models. N = 50 simulations with *p_driver_* = 6 × 10^−4^ are shown. (f) Axial image in the corticomedullary contrast phase of a representative case (K523) showing budding structure on the tumour surface (red arrow). Outlines in red reflect the tumour contour giving volumetric tumour coverage. (g) Maps of tumour regions with the number of subclones colour coded in a representative case. Hues from red to purple to blue reflect decreasing number of subclones. “Low” and “High” reflect 1 and 4 subclones, respectively. (h) The number of subclones as a function of ultimate tumour size in the TRACERx Renal study, overlaid with KDE based on simulated data. Tumours with a size up to 7 cm and with radiologically evident budding structures are highlighted (orange). Contours reflect 90% probability density based on *in-silico* tumours under Surface Growth (red) and under Volume Growth (blue), respectively in Figure 6b.

We investigated temporal features of clonal diversification through *in-silico* time-course analysis. Overall, the number of subclones remained limited in Volume Growth models but increased over time before reaching a plateau in Surface Growth models (Figure 5a-b, Extended Data Figure 8). Thus, Surface Growth and Volume Growth models initially showed similar extent of clonal diversification with subsequent divergence. Notably, when necrosis was implemented, Volume Growth models were minimally impacted, but we observe dramatic reduction in clonal diversity at later stages of tumour growth in Surface Growth models, especially when additive driver advantages were implemented (Extended Data Figure 8). This reflected a “pruning” effect on the clonal structure with elimination of the less fit subclones. These observations reconciled previous observation of non-monotonic relationship between tumour size and number of clones, including the collapse of clonal diversity at very large tumour sizes^7^.

As the birth of a new subclone is defined by the acquisition of new driver events in a tumour voxel, expectedly, Surface Growth models, which showed more extensive clonal diversification, accumulated a larger number of drivers, at a faster rate, than Volume Growth models (Extended Data Figure 8). Nevertheless, in contrast to the rate of clonal diversification, the number of accumulated drivers increased monotonically over time in the Surface Growth models. Repeat simulations under Surface Growth clearly exhibited an altered direction of “evolutionary flows”, indicating out-competence of advantageous subclones and reduction of overall clonal diversity at later stages (Figure 5c). Underlying these observations, Surface Growth led to polynomial growth with longer time to reach the stopping condition, while Volume Growth resulted in exponential growth (Extended Data Figure 8). The faster growth rate in Volume Growth models means a large contribution of parental clone to overall tumour growth and shorter time for advantageous subclones to outgrow and compete, leading to tumours with limited diversification.

### Early indication of evolutionary potential

We have previously shown that evolutionary features correlate with clinical outcomes and could be used to guide patient management^7^. Therefore, it is of particular interest to examine whether the computational model could be used to suggest predictive features for the likely evolutionary trajectories and therefore clinical behaviour of individual tumours.

Of note, early-stage tumours under distinct growth modes appeared indistinguishable with respect to the number of subclones (Figure 5a). We specifically investigated features that could indicate subsequent subclonal diversification in the Surface Growth model and noted the appearance and outgrowth of budding structures (Figure 5d). Notably, as the tumour grew with gained fitness, the contour circularity of a tumour slice decreased initially and then recovered, concomitant with the initial increase and subsequent reduction in clonal diversity, respectively (Figure 5e). Exploratory simulations attempting at “replaying” evolution (i.e., re-simulating clonal evolution from a historical tumour state with established clonal structure as a starting point) starting from different tumour sizes suggested that evolution was more repeatable if starting from a historical tumour state with budding structures emerging (Supplementary Note 7, Extended Data Figures 9-10).

With respect to the above findings, the radiological features of 46 tumours with a diameter of < 7*cm* in the TRACERx Renal study were evaluated (Supplementary Table 4). By qualitative examination of radiological images, budding structures were apparent at the surface of 16 tumours and evident in the tumour ex-vivo (one representative K523 shown in Figure 5f-g). In this case, adjacent to the budding structures were regions with high clonal diversity, consistent with Surface Growth models. Interestingly, the presence of budding in these 16 sub-7cm tumours exhibiting increasing diversity as a function of tumour size, combined with the absence of budding and low diversity in some larger tumours (Figure 5h), showed clear concordance with the temporal trend of clonal diversification and morphological variation observed in the Surface Growth models. The failure of this trend to continue in larger tumours supports the observed collapse of subclonal diversity in simulated tumours under Surface Growth.

## Discussion

**Figure 6:**
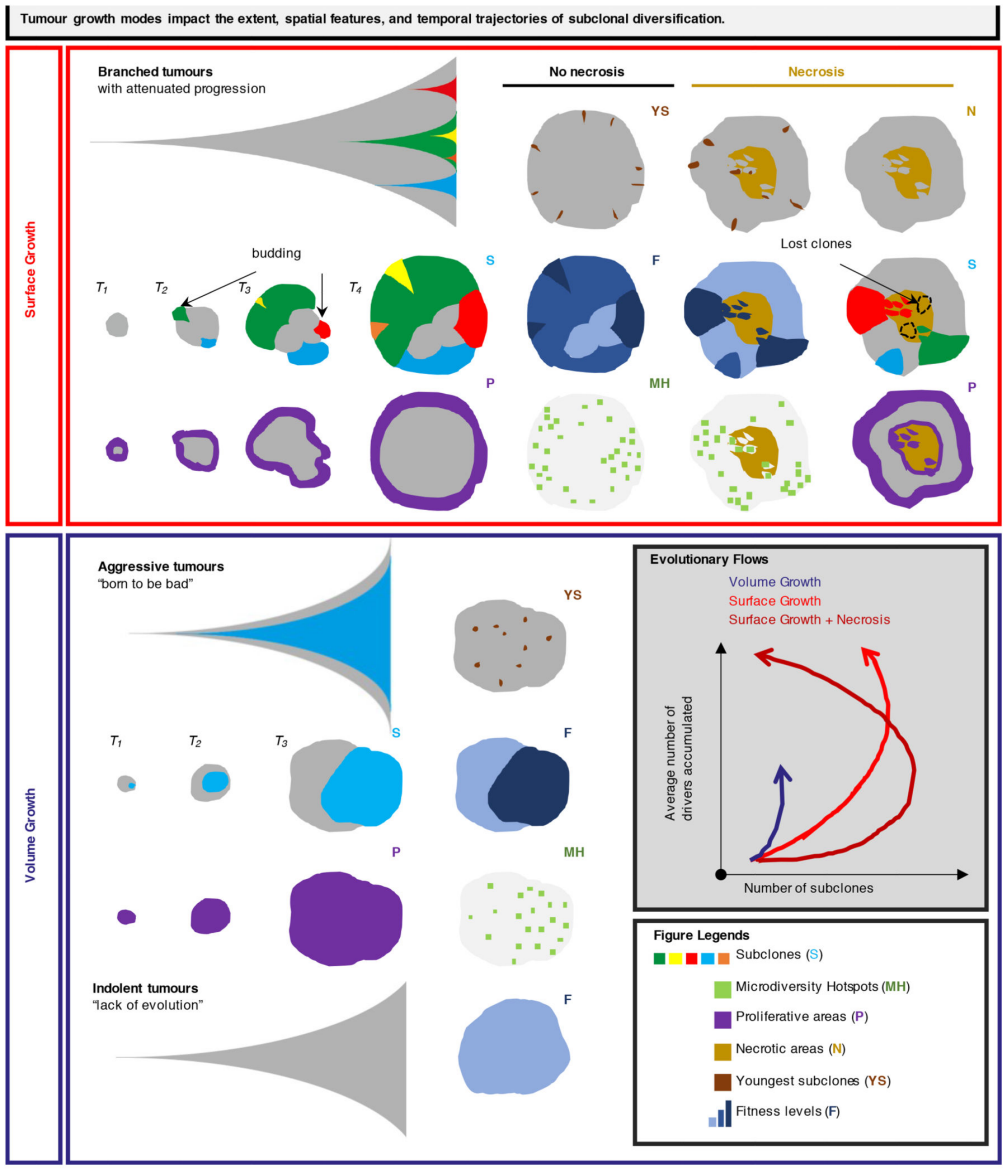
Summary diagram Tumour growth modes impact the extent, spatial features, and temporal trajectories of clonal diversification. Surface Growth models lead to attenuated progression with extensive subclonal diversification, reflective of branched evolution. At the early stage, the birth and outgrowth of proliferatively advantageous subclones causes the formation of surface budding structures and consequently a distorted tumour contour. As these subclones grow to collectively constitute the tumour frontier at a later stage, the tumour contour returns to a more circular shape with enrichment of youngest subclones and microdiversity hotspots and enhancement of fitness near the tumour margin. The incorporation of central necrosis causes the loss of macrodiversity but at the same time permits continued subclonal diversification in the tumour interior, evidenced by the enrichment of youngest subclones and microdiversity hotspots. In contrast, Volume Growth models give rise to dichotomous patterns of tumour growth and clonal evolution – developing tumours that are either indolent with “lack of evolution” or aggressive with early birth of fitter subclone and rapid progression. The distributions of youngest subclones, microdiversity hotspots, and fitness in Volume Growth models are more uniform than those in Surface Growth models.

Genetic intra-tumour heterogeneity arises when clonally related population of cells in the tumour acquire distinct genomic alterations, endowing the subclones with a range of fitness advantages. Our understanding of how major subclones sculpt evolutionary trajectories, has been gleaned primarily from multi-region sampling and deep sequencing. However, understanding of the temporal features of clonal evolution and the ability to predict evolutionary trajectories remain limited. Therefore, we focus on spatial and temporal characterisation of clonal diversity to elucidate predictive features for evolutionary potential, summarised in Figure 6. To this end, we developed an agent-based model to study tumour growth and clonal evolution, with a focus on examining the contribution of different modes of growth, namely, Surface and Volume Growth, and the presence or absence of central necrosis to spatial and temporal features of clonal diversity.

Adding to previous modelling work on spatial elements of tumour growth^26–29,33–37^, our model demonstrated that growth modes impact subclonal diversification. Specifically, Volume Growth resulted in either limited evidence of evolution or punctuated evolution with early fixation of a fit clone, Surface Growth gave rise to branched evolution with extensive subclonal diversification (Figure 6). Intriguingly, Surface Growth models revealed a non-monotonic variation in clonal diversity over time with a dramatic collapse of diversity at large tumour sizes, consistent with the apparent relationship between static data of tumour size and clonal diversity in the TRACERx Renal study. These temporal features of clonal diversity informed by the model raise the possibility that evolutionary modes are not a static property but instead can undergo a dynamic switch from a branched to an apparently punctuated sub-type, with peak diversity occurring in the past, during tumour development.

Spatial analyses further uncovered that microdiversity hotspots and youngest subclones were more uniformly distributed in Volume Growth models while predominantly near the tumour margin in Surface Growth models (Figure 6). As in the model, spatial distribution of microdiversity hotspots exhibited a power law pattern in ccRCCs. Strikingly, the exponent of the power law was associated with previously described different classes of ccRCC evolution. Tumours with attenuated progression had a larger exponent, which is consistent with their more branched phylogenetic trees. Both indolent mono-driver and aggressive poly-driver tumours had lower exponents suggesting Volume Growth patterns, with the aggressive tumours simply determined in our model by the early acquisition of multiple strong drivers.

Investigation on the impact of necrosis in our model further broadened our understanding of the spatial and temporal features of clonal diversification. (Figure 6). Incorporation of necrosis led to enhanced fitness in the tumour interior, suggestive of selection of fitter clones, in keeping with our recent study^23^. Furthermore, with necrosis, tumours under Surface Growth harboured additional microdiversity hotspots and youngest subclones at the centre. The pattern of youngest subclones was corroborated by the analysis of sequencing data and further supported by histological evidence of interface between tumour and acellular areas, suggesting that Surface Growth with necrosis incorporated could explain the evolution of some ccRCCs. Our recent work in the context of TRACERx Renal demonstrated that metastasis-competent subclones are enriched at the tumour centre, suggesting that environmental factors favoured their selection, possibly through acquisition of advantageous traits like epithelial-to-mesenchymal transition. In the current study, we present a complementary, and non-exclusive, perspective that necrosis could accelerate the rate of evolution, via turnover of tumour mass, to achieve enhanced fitness in the tumour centre.

Finally, tracking advantageous subclones over time *in silico* illuminated the rapid increase of their prevalence in small tumours, marked by the appearance of budding structures, concomitant with subclonal diversification in Surface Growth models. Intriguingly, budding structures were radiologically apparent in 16 early-stage ccRCC tumours, a subset of which already showed high clonal diversity, by molecular profiling. While budding structures in our model arose from advantageous subclonal outgrowth, alternative mechanisms cannot be excluded^38 33^.

To conclude, we have developed a model that enables us to understand how spatial patterns of growth and necrosis determine patterns of clonal diversity in space and time (Figure 6). We validate our finding using patient data, thereby opening the potential for predicting future clinical behaviour, precision medicine’s holy grail.

## Methods

### Computational model

Tumour growth and clonal evolution in a spatio-temporal context have increasingly been studied with the aid of computational models that incorporate spatial elements of tumour growth and acquisition of genomic alterations^26,27,33,34^. Spatial patterns of tumour growth^26–29^ have been shown to impact the ability to classify neutral evolution in contrast to selection, suggesting that spatial growth of a structured population interplays with evolutionary forces (driver acquisition, selection, and genetic drift) to shape the spatial patterning of subclones.

In the present study, to establish an understanding of the spatial and temporal features of clonal diversification and to enhance the ability to predict evolutionary trajectories in ccRCCs, we constructed a coarse-grained cellular automaton model to simulate tumour growth and clonal evolution. A basic model unit reflects a tumour volume of 1 × 1 × 1 *mm* ^3^, referred to as a “tumour voxel”. The full simulation lattice comprises 200 × 200 × 200 lattice sites, each of which can accommodate a single tumour voxel when a tumour grows. As a simulation proceeds, tumour voxels stochastically undergo growth, death, and acquisition of driver events upon growth (Extended Data Figure 3). The subsequent sections detail the model components and assumptions.

### Growth and death

Tumour voxels stochastically undergo growth and death, with baseline probabilities per simulation step of *p_growt h_* = 0.25 and *p_deat h_* = 0.05, respectively. Upon death, a tumour voxel is removed from the simulation lattice, rendering the site empty and available for accommodating new tumour voxels. Two different modes of spatial tumour growth are considered: Surface Growth and Volume Growth (Figure 1c). For Surface Growth, proliferation only takes place when space is available, namely, when at least one of the 26 neighbouring lattice sites of the tumour voxel selected to divide is empty. Upon duplication of a parent tumour voxel, one child tumour voxel retains the position of the parent while the other is placed at a randomly selected adjacent empty site. For Volume Growth, all tumour voxels can proliferate; upon duplication, one child tumour voxel retains the position of the parent while the other is placed at a selected adjacent site according to the rule described below and pushes tumour voxels in that orientation outward. The process for selecting an adjacent site includes two steps: (1) to randomly sample 10 candidate positions out of the 26 neighbouring lattice sites; (2) to select the orientation (i.e., pointing from the position of the parent tumour voxel to the candidate position) giving the smallest distance from the tumour surface, similar to the algorithm described in Waclaw et al (2015)^34^.

### Driver events

A panel of 26 ccRCC drivers that were highlighted in Turajlic et al. 2018, including mutations in 12 genes and 14 somatic copy number alterations (SCNAs), are considered in the present work (Extended Data Figure 1). For simplicity, the selective advantage conferred by a driver is assumed to manifest as growth advantage.

Two ways of implementing selective advantages are considered and referred to as “saturated” and “additive” driver advantage models. In the saturated driver advantage model, *p_growt h_* of a tumour voxel can be at one of the three levels $\left\{p_{growt\;h\;}^{\left(initial\;\right)},p_{growt\;h\;}^{\left(moderate\;\right)},p_{growt\;h\;}^{\left(maximal\;\right)}\right\}$ . Each driver endows a tumour voxel with one of these growth probabilities, and the relative differences in selective advantage of drivers are assumed to reflect their association with the Ki67 score in tumour regions (Extended Data Figure 1) and their frequencies in the clinical cohort^7^ (Turajlic et al. 2018). In a general form, $p_{growt\;h\;}^{\left(moderate\;\right)}=g(s)p_{growt\;h\;}^{\left(initial\right)}$ and $p_{growt\;h\;}^{\left(maximal\;\right)}=h(s)p_{growt\;h\;}^{\left(\text{i}nitial\right)}$ are functions of the baseline growth probability, where *h*(*s*) ≥ *g*(*s*) ≥ 1 reflect the growth advantages relative to the baseline. As one specific implementation, $p_{growt\;h\;}^{\left(initial\;\right)}=0.25$ , $p_{growt\;h\;}^{\left(moderate\;\right)}=(1+s)p_{growt\;h\;}^{\left(\text{i}nitial\;\right)}$ and $p_{growt\;h\;}^{\left(maximal\;\right)}=(1+s{)}^{2}p_{growt\;h}^{\left(initial\right)}$ , where 0 ≤ *s* ≤1 reflects the selective advantage. For simplicity, individual driver gene mutations are assigned with $p_{growt\;h\;}^{\left(initial\right)}$ , whereas four SCNAs with strong association with Ki67 score (7q gain, 20q gain, 4q loss, and 8p loss) are assumed to be the strongest drivers assigned with $p_{growt\;h\;}^{\left(maximal\right)}$ and therefore their acquisition would lead to the biggest increase in growth probability. Importantly, the saturated model is implemented with only two levels of selective advantage, and the growth probability of a tumour voxel becomes saturated at 1 if acquiring the strongest driver. In comparison, the additive driver advantage model has a more graduated implementation of selective advantage. In this implementation, each driver adds a certain amount of growth probability to the tumour voxel that acquires the driver, namely, $p_{growt\;h\;}=p_{growt\;h\;}^{\left(initial\;\right)}+\sum_{k}p_{growt\;h\_k}$ , where *p_growth_k_* reflects the amount of growth probability added by driver *k* (Extended Data Figure 2). *p_growt h_* is set to one if the calculated probability exceeds one. The amount *p_growth_k_* varies between drivers, reflecting different strengths of their association with Ki67 score (Extended Data Figure 1). Three different scenarios were explored to reflect different amounts of growth probability endowed by drivers on average, as determined by *s_k_* of the weakest driver, namely, min(*s_k_*), and the difference in *s_k_* between consecutive two drivers in their advantages, namely, Δ*s_k_*. (Extended Data Figure 2).

Upon proliferation of a parent tumour voxel, child tumour voxels inherit existing driver events harboured by the parent tumour voxel and stochastically acquire new drivers. Individual driver gene mutations are assumed to be acquired with a greater probability (*p_driver_*) than SCNAs (0.001*p_driver_*). A second mutation in the same gene is assumed to never occur in the same tumour voxel, but multiple independent, distinct mutations in the same gene may be acquired in parallel within a simulated tumour in different tumour voxels. As the majority of ccRCCs have clonal *VHL* inactivation events, in general and in the TRACERx Renal cohort^7^, the founder tumour voxel is assumed to harbour *VHL* inactivation together with 3p loss as a clonal event. The subpopulation of tumour voxels that only harbour these two events is referred to as the parental clone. Based on their association with a high weighted genome instability index (wGII) in the data from the TRACERx Renal study^7^, and functional evidence^31,32^, mutations in *PBRM1* or *BAP1*, are assumed to enhance the probability of SCNA acquisition (to *p_driver_*). A range of driver acquisition probabilities have been studied to explore its impact on patterns we investigate (See in Supplementary Note 8 considerations for the selection of *p_driver_* values in the coarse-grained model). Mutations and acquisition of SCNAs are assumed to be proliferation-dependent, which imply DNA replication and chromosome mis-segregation as the main source of genomic alterations. Lastly, the selective advantage endowed by a driver is assumed to be fixed, so the variation in driver advantage dependent on changing environments is not considered in the current study.

### Necrosis

Building upon our previous work^23^, necrosis is implemented in a subset of model conditions to evaluate its impact on features of clonal diversification. Specifically, tumour voxels located at a distance greater than *d_necrosis_* = 15 *mm* from the tumour surface undergo death with a probability of *p_necrosis_*. A probability of *p_necrosis_* = 0.5 is used in this study, in keeping with our previous work. Like the spontaneous death described above, upon necrosis-induced death, a tumour voxel is removed from the simulation lattice, rendering the site empty and available for accommodating new tumour voxels.

### Simulation

The procedure for simulating events of death, proliferation, and acquisition of driver events is illustrated in a flow diagram (Extended Data Figure 3). Briefly, each simulation starts from a single tumour voxel (i.e., founder tumour voxel) that harbours a *VHL* mutation and 3p loss as truncal events, placed at the centre of the lattice, (*x*_0_, *y*_0_, *z*_0_). During the evaluation of possible death events, for each of all tumour voxels alive, *p_deat h_* is compared to a random number generated between [0,1]. If *p_deat h_* is larger, a death event occurs, resulting in the lattice site freed for accommodating a newly born tumour voxel in the future. During the evaluation of possible proliferation events, for each of all valid tumour voxels (see above for the difference between Surface Growth and Volume Growth), *p_growt h_* is determined according to the driver events harboured (see above for the difference between “saturated” and “additive” models of fitness advantage) and compared to a random number generated between [0,1]. If *p_growt h_* is larger, a proliferation event occurs, resulting in a new tumour voxel created nearby. During the evaluation of possible acquisition of driver events, for each of all daughter tumour voxels just arising from proliferation and for each of the ccRCC drivers, *p_driver_* is compared to a random number generated between [0,1]. If *p_driver_* is larger and that driver is not currently harboured by the tumour voxel, acquisition of the driver takes place in the given tumour voxel. In a subset of simulations, necrosis is implemented. During the evaluation of necrotic death events, for each of all tumour voxels alive, if it’s located at a distance of greater than *d_necrosis_* from the tumour surface, *p_necrosis_* is compared to a random number generated between [0,1]. If *p_necrosis_* is larger, a necrotic death event occurs, resulting in the lattice site freed for accommodating a newly born tumour voxel in the future. The simulation runs until the tumour grows to at least 1 million tumour voxels after the last simulation step. The computer code is written in CUDA C++.

### Evolutionary replay

The procedure for simulating evolutionary replay is illustrated in a flow diagram (Extended Data Figure 9). Briefly, a preparation step is performed to create evolutionary snapshots of a simulated tumour at different time points. Specifically, each snapshot contains precise information about the positions, subclone identities, and drivers of all tumour voxels. At the beginning of evolutionary replay, *N* replicate tumours are reconstructed, each with a copy of the same evolutionary snapshot at a given time point *t*. Then, these replicate tumours undergo the events described above, each with a different unique random seed, and grow to the predefined stopping size. Evolutionary outcomes from these replicate tumours are evaluated and compared.

### Model analyses

#### Levels of analysis

Analyses are conducted at three different levels (Figure 1D): (1) whole tumour level, which takes into account all tumour voxels in the 3D volume; (2) tumour slice level, which takes into account all tumour voxels within a 2D plane (*z* = *z*_0_); (3) regional biopsy level, which takes into account tumour voxels within regional biopsies. A regional biopsy is defined as all tumour voxels within a region in the 2D slice. Spatially uniform sampling is performed in this study. This process is carried out by locating the centres of candidate regional biopsies in the 200*mm* × 200*mm* 2D lattice with a spacing of 20 *mm* and collecting all voxels within a distance of 5 *mm* from each biopsy centre.

#### Cancer cell fraction (CCF) of subclones

CCF of a subclone is calculated as the number of tumour voxels that belong to a subclone divided by the total number tumour voxels in the domain of interest, depending on the level of analyses. A subclone is identified by a set of driver events, shared by a subpopulation of tumour voxels, which are accumulated within the subclone-initiating tumour voxel. A subclone-initiating tumour voxel is defined as a tumour voxel that acquires a new driver event upon birth. A subclone is considered detectable if the CCF is greater than 0.01.

#### Shannon diversity index

As a measure of clonal diversity, the Shannon diversity index is defined as $S=\sum_{i}-f_{i}\ln f_{i}$ , where *f_i_* is the CCF of the subclone *i*. All subclones are considered in this calculation.

#### Number of drivers accumulated

Drivers (including both mutations and SCNAs) harboured by each tumour voxel within a tumour slice are counted at each time point. The average number of drivers among all tumour voxels is then calculated.

#### Fitness

Fitness of a tumour voxel is defined as the instantaneous growth probability, *p_growt h_*, dependent on the set of drivers harboured by that tumour voxel. In representative cases, fitness of tumour voxels is mapped within a 2D tumour slice. To examine the spatial features of tumour fitness, the distances from every tumour voxel to the centre of a tumour slice and to the nearest point along the tumour contour are calculated, respectively. The central-most 10% of tumour voxels, with the shortest distances to the centre, and the marginal-most 10% of tumour voxels, with the shortest distances to the margin, are sampled to calculate a sample-average fitness. Additionally, another 10% of tumour voxels is randomly sampled for comparison. The ratio of the mean fitness of the central-most 10% of tumour voxels to that of the marginal-most 10% of tumour voxels, denoted as “Ratio_C2M” is calculated for each simulation.

#### Microdiversity

Microdiversity is defined as the number of subclones contained in a 3 × 3 *mm* ^2^ region within the tumour slice. In representative cases, microdiversity is spatially mapped within a tumour slice, by sliding a 3 × 3 *mm* ^2^ spatial window throughout the tumour slice. Microdiversity hotpots are defined as a subset of these small regions with 5 or more subclones. The distance from a microdiversity hotspot to the centre of a tumour slice is referred to as the distance to tumour centre (*d_1_*). The distance from a microdiversity hotspot to the nearest point along the tumour contour is referred to as the distance to tumour margin (*d_2_*). The normalised distance to tumour centre is defined as *d* = *d*_1_/(*d*_1_ + *d*_2_). Cumulative probability distribution, *P*(*D* ≤ *d*), of *d* is generated by combining microdiversity hotspots from repeat simulations. The power law exponent is obtained by bootstrapping 100 samples of 400 hotspots per sample and fitting a power law function to the cumulative probability distribution of *d* in each sample.

#### Youngest subclones

Youngest subclones are defined as the subclones that emerge closest to the end of a simulation. For the analysis of their spatial distribution, we recorded the 100 youngest subclones within a 2D tumour slice and, for each of them, measured the distance from its position to the tumour margin. The mean of the distances for these 100 subclones was calculated for comparison between different replicate simulations.

#### Contour circularity

Tumour contours are determined from 2D tumour slices. Smoothening is performed for each tumour voxel along the tumour contour by calculating the mean (*x*, *y*) position of this tumour voxel and adjacent tumour voxels. After smoothening, contour circularity is calculated as: $circularity\;=\frac{4\pi \cdot Area}{Perimeter}$ .

#### Temporal analysis

For the time-course study, 2D tumour slices are collected over multiple time points. The number of subclones is counted within each historical tumour slice. Kernel density estimation with a Gaussian kernel is performed with respect to the number of subclones and the diameter of the tumour slice, based on all simulations under a given model condition, to produce a continuous density estimate.

### Analyses of tumour data

#### TRACERx Renal cohort

79 tumour sections of 66 unique primary tumours are included in this study; see the exclusion criterion in our previous publication^23^.

#### CT images

Contrast-enhanced Computed Tomography (CT) images were obtained using standard-of-care imaging sequences in 91 patients and curated using a local research picture archiving and communication system (PACS) (based on the Extensible Neuroimaging Archive Toolkit

(XNAT) platform^39^). Outlines were drawn by consensus between an oncologist (S.T.C.S.) and a radiologist (D.A.) giving volumetric tumour coverage, from which image strips were prepared for rapid visualisation of all tumour slices for all patients using an in-house script written in python.

To detect presence or absence of budding structures in the contoured CT data, qualitative assessment of contoured tumours was performed by X.F. and S.T.C.S and verified by a radiologist (D.A.).

#### Histological analysis

Haematoxylin and eosin-stained histological sections representative cases were evaluated by two pathologists (J.I.L./C.E.S.). Each sample was qualitatively assessed for the relative amount of viable tumour cells, fibrosis and presence of necrosis.

#### Microdiversity

Spatial maps of regional clone diversity are created for two representative tumour sections. In these maps, regions are colour-coded based on the number of subclones. Regions that harbour at least one subclone are treated as a proxy for microdiversity hotspots defined in the model analysis. In total, there are 606 regions from 54 tumours that satisfy this criterion. In evaluation of association between microdiversity features and clinical annotations, subsets of tumours are considered. Subsets with different relapse statuses consist of 270 (“Relapse”) and 336 regions (“No relapse”), respectively. Subsets with different rates of disease progression consist of 276 (“No progression”), 265 (“Attenuated progression”), and 65 regions (“Rapid progression”), respectively. For tumour regions, the normalised distance to tumour centre is measured as described above in “Model analysis - Microdiversity”.

#### Parallel evolution

Spatial maps of parallel mutational events in *PBRM1* and in *BAP1*, respectively, are created for two representative tumours. In these maps, regions are coloured differently according to different parallel mutational events. Regions that harbour more than one event are indicated with multiple colours. To study the spatial distribution of mutational events with limited clonal expansion, the maximum distance from an event spanning a single region to the tumour margin is measured.

#### Statistical analysis

The two-sided Wilcoxon’s rank test was performed to compare a particular measurement between different conditions. Statistical significance is annotated within box plots using stat_compare_means(method = “wilcox.test”, label = “p.signif”) in R.

Bootstrapping was performed to generate 100 random samples of 400 microdiversity hotspots per sample with replacement, using random.choice() in Python. The power law exponent was then determined by fitting a power law function to the cumulative probability distribution from each sample, using scipy.optimize.curve_fit() in Python.

The Quantile-Quantile (Q-Q) plot was generated to compare the actual distribution of microdiversity hotspots to a power law distribution with the exponent as the median of fitted values in bootstrapping, using statsmodels.graphics.gofplots.qqplot() in Python.

Kernel density estimation was performed for simulations with respect to the size of tumour slice and the number of subclones, using seaborn.jointplot(kind=“kde”) in Python.

R version 3.6.2 and Python version 3.7.7 are used for these analyses.

## Extended Data

**Figure:**
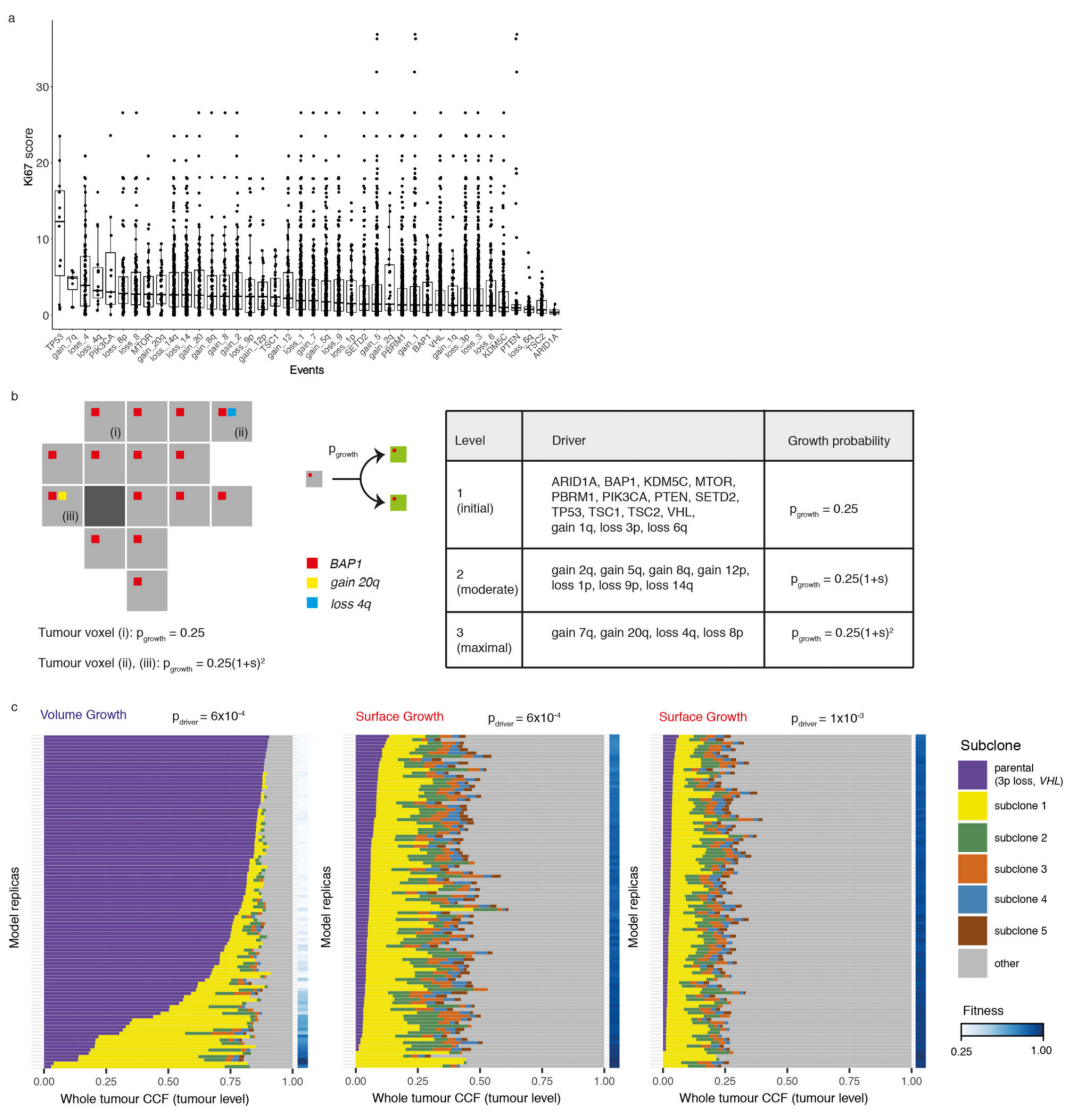


**Figure:**
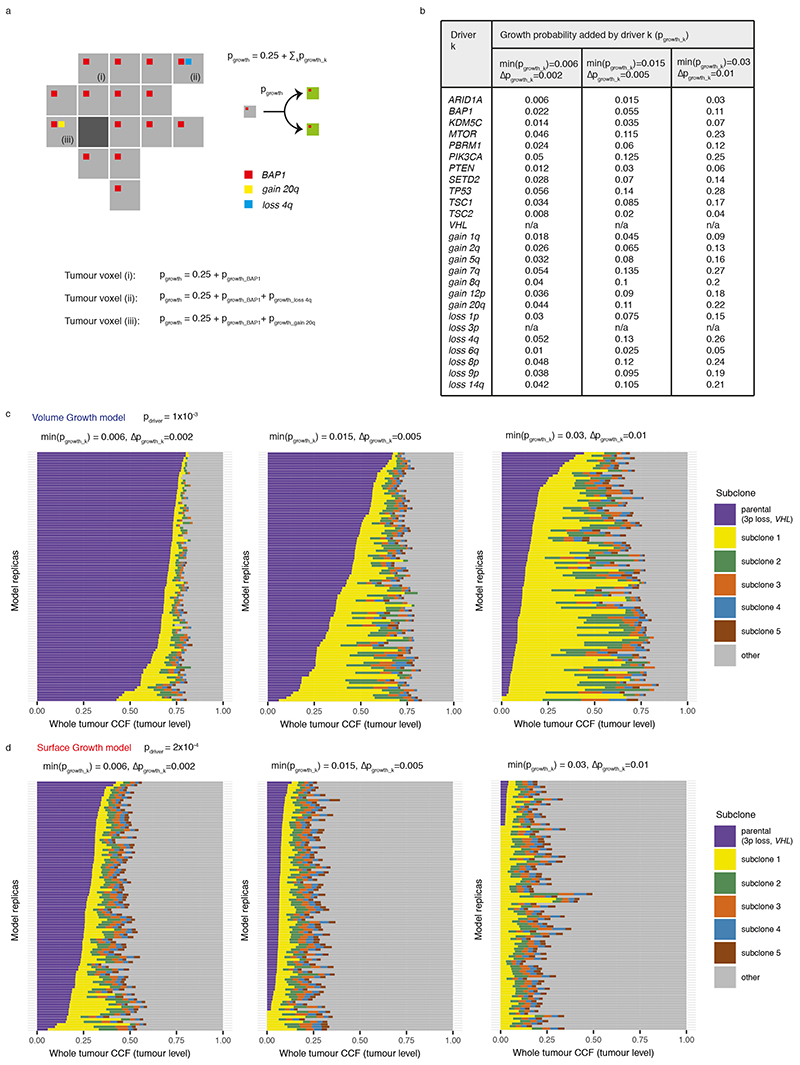


**Figure:**
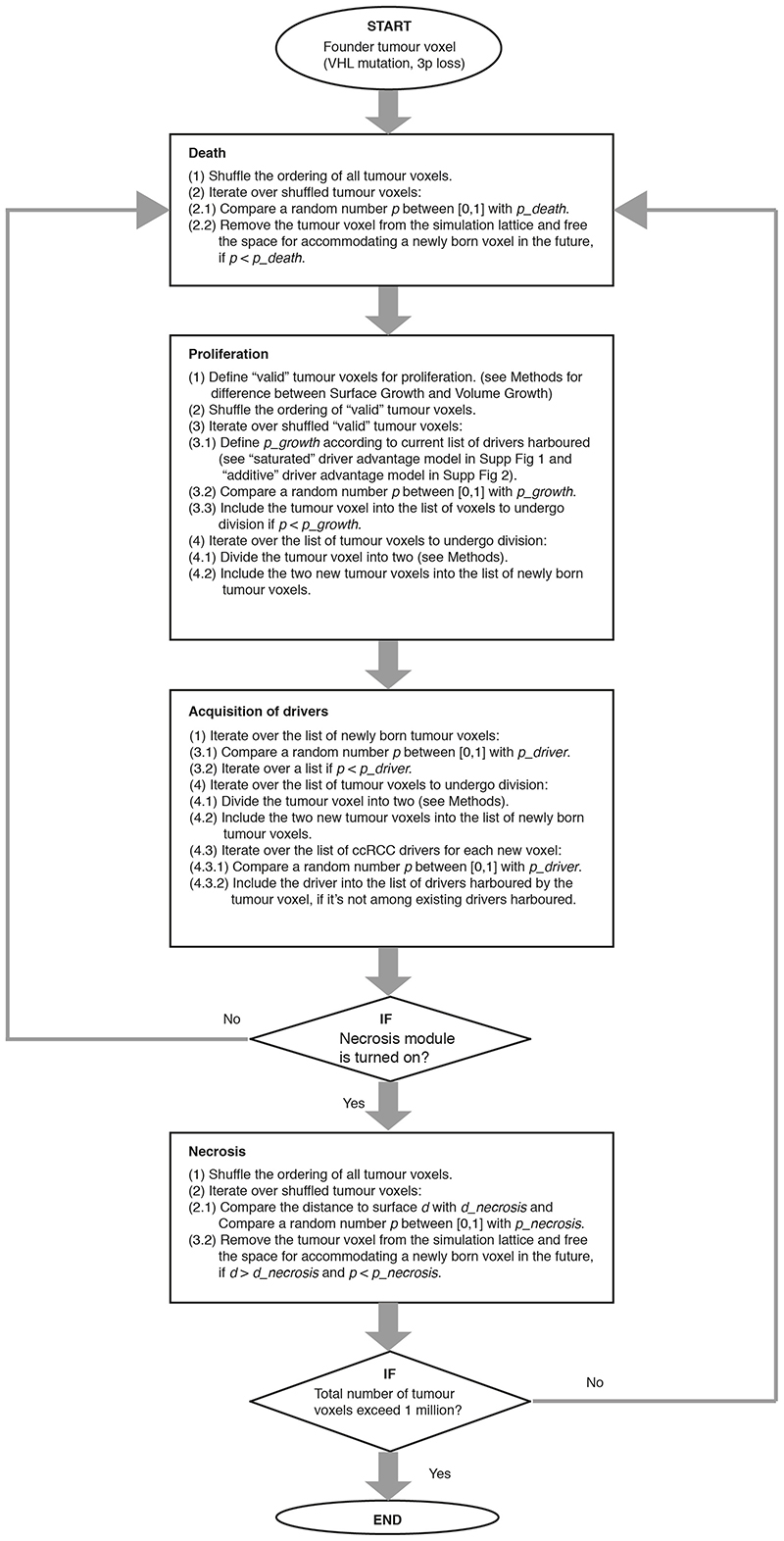


**Figure:**
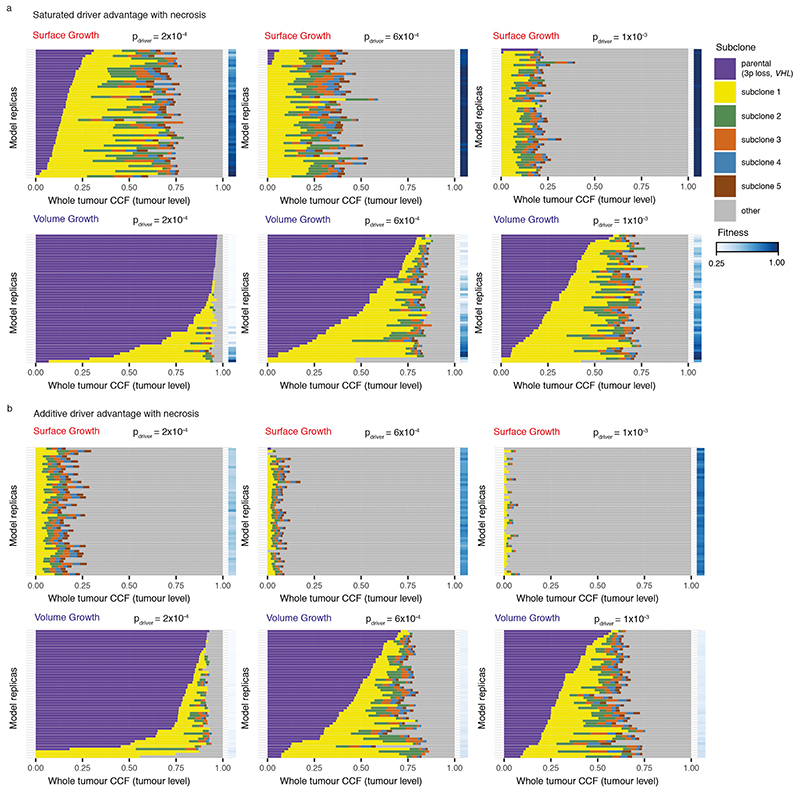


**Figure:**
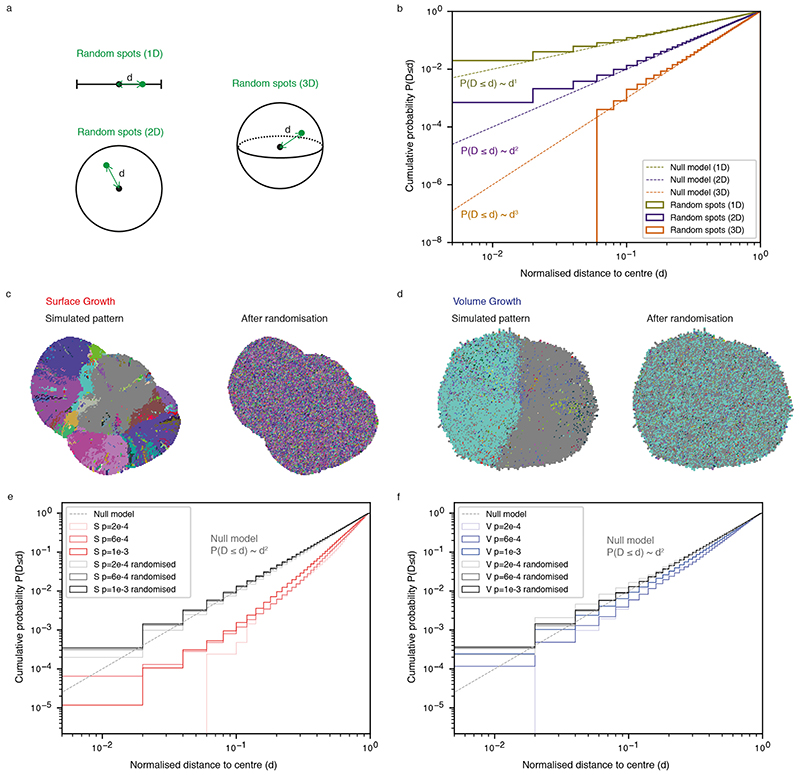


**Figure:**
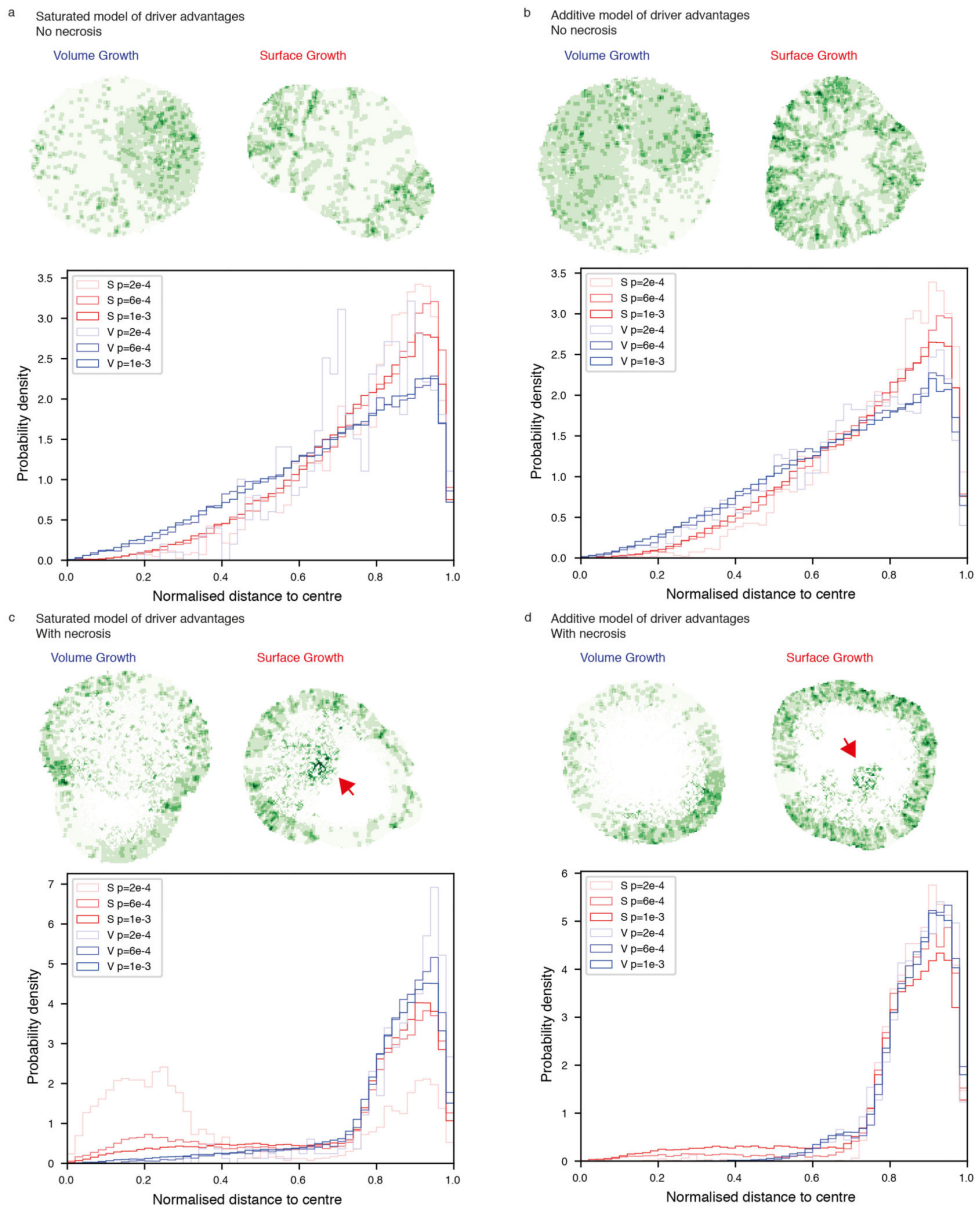


**Figure:**
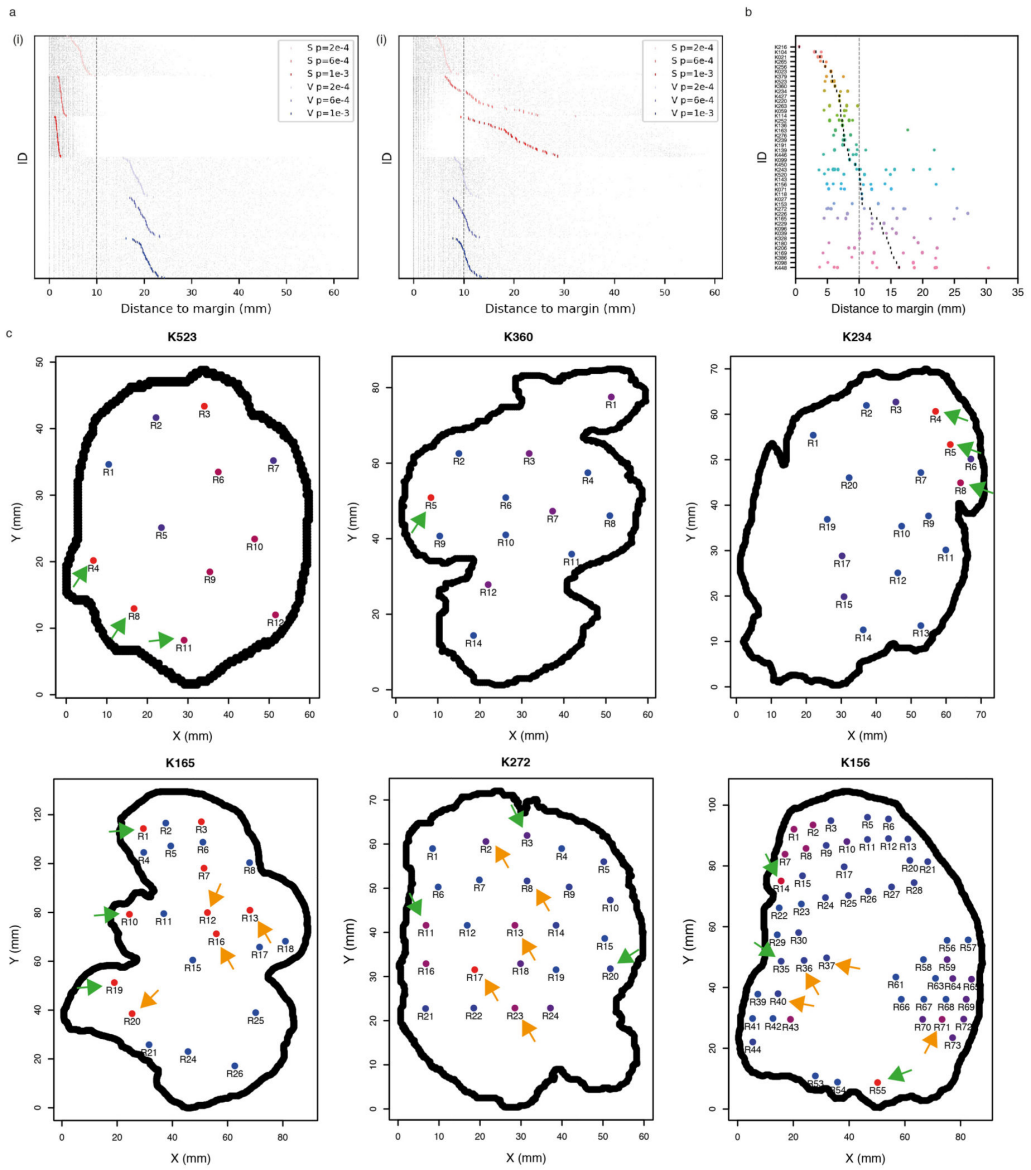


**Figure:**
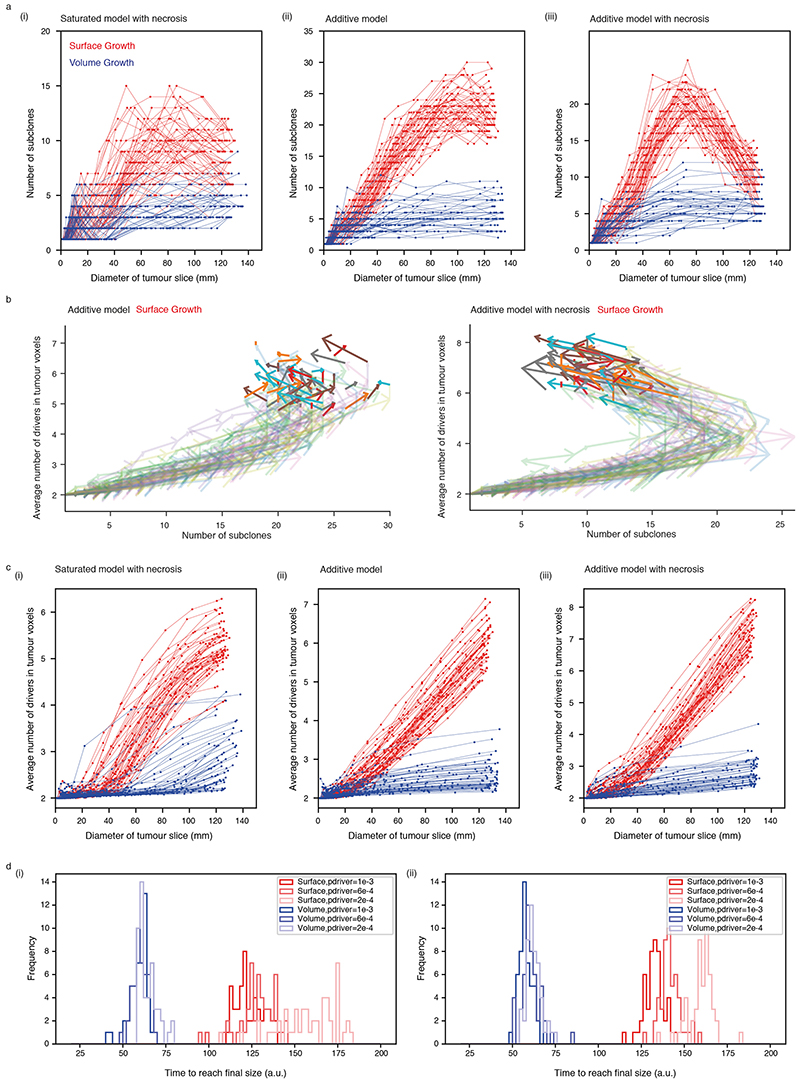


**Figure:**
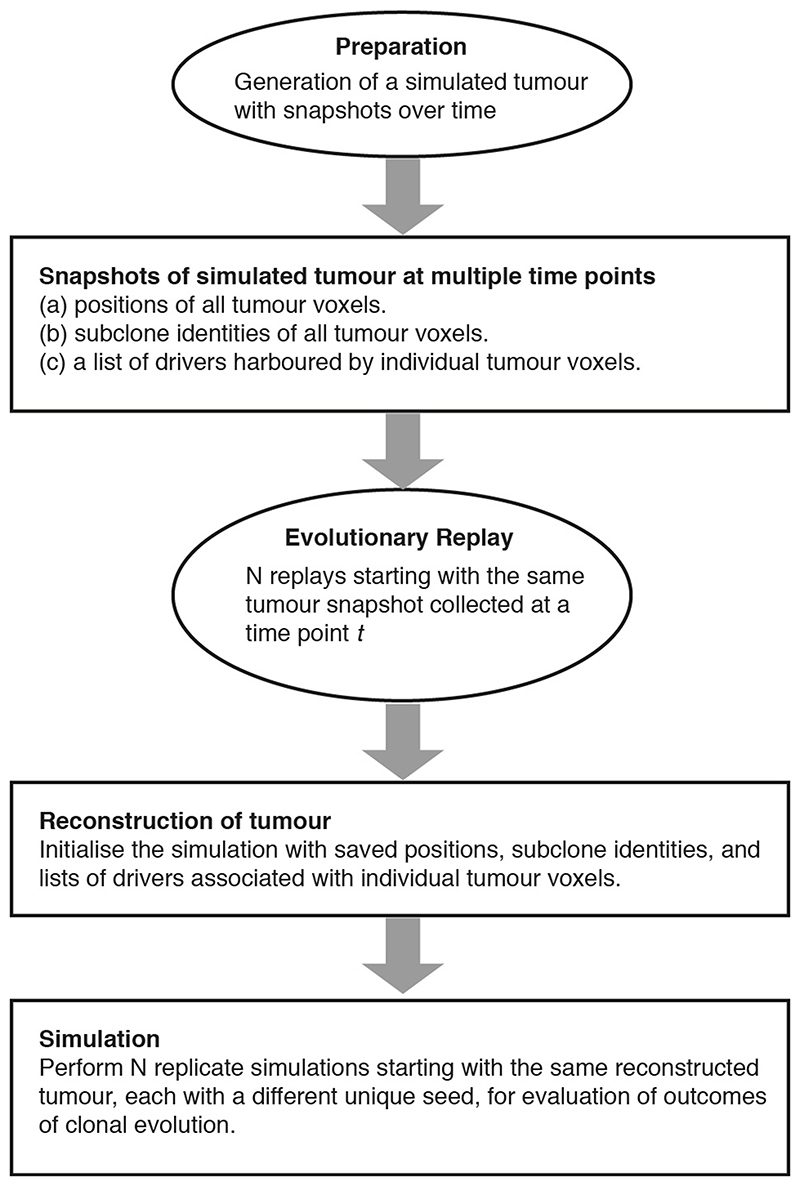


**Figure:**
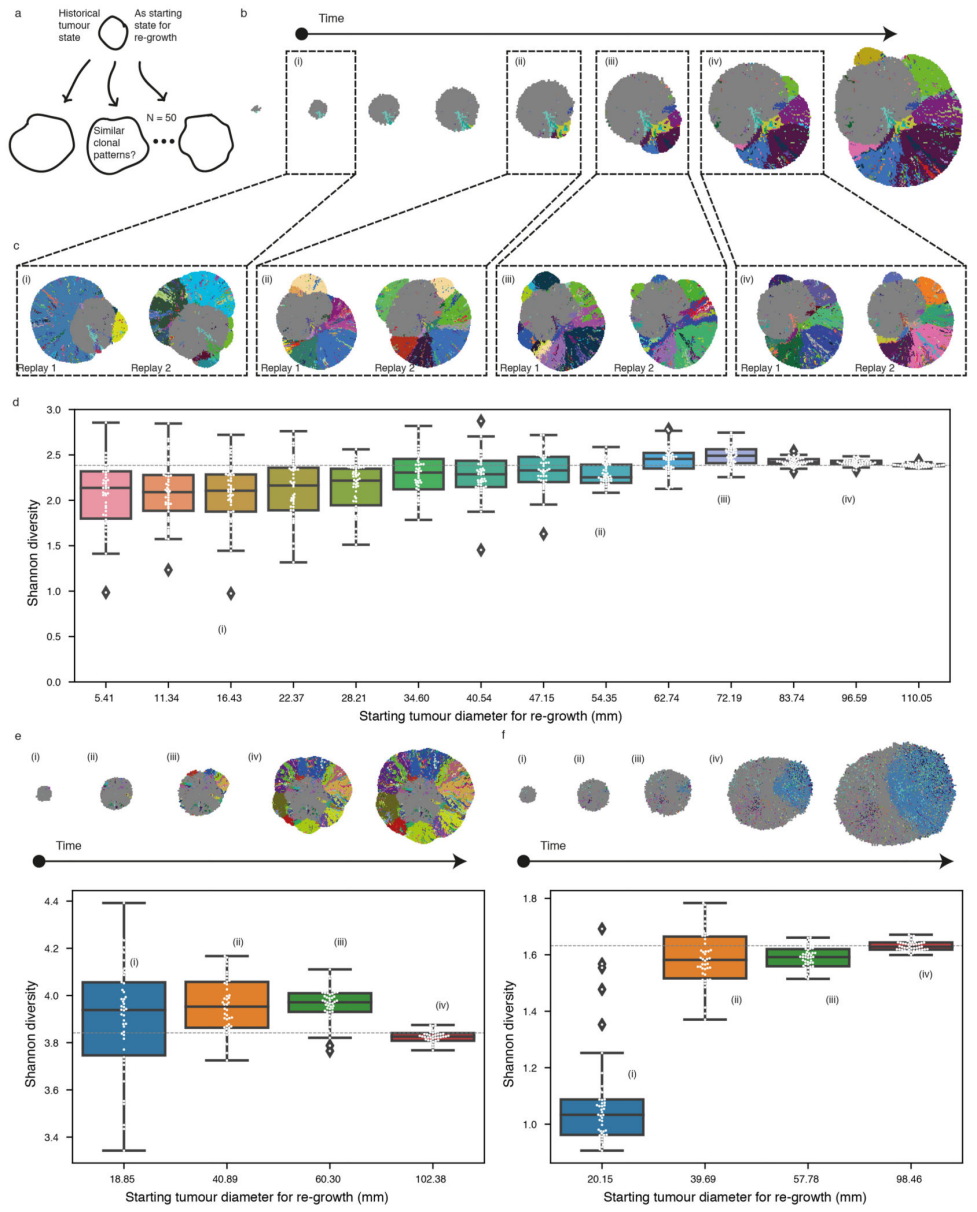


## Supplementary Material

Source Data for Extended Data Figure 1

Source Data for Extended Data Figure 2

Source Data for Extended Data Figure 4

Source Data for Extended Data Figure 5

Source Data for Extended Data Figure 6

Source Data for Extended Data Figure 7

Source Data for Extended Data Figure 8

Source Data for Extended Data Figure 10

Source Data for Figure 2

Source Data for Figure 3

Source Data for Figure 4

Source Data for Figure 5

Supplementary Information

Supplementary Tables

## Data Availability

Multi-region sequencing data that support the analysis in this study were published in the previous TRACERx Renal study^7^ and are deposited on European Genome-Phenome Archive https://ega-archive.org/studies/EGAS00001002793. Data on spatial features of microdiversity and parallel evolution and characterisation of budding structures in tumours are provided in Supplementary Tables. Source data for Main Figures and Extended Data Figures are submitted together with this manuscript and are available on GitHub repositories https://github.com/FrancisCrickInstitute/tumour-growth-patterns-impact-evolution and https://github.com/iamfuxiao/tumour-growth-patterns-impact-evolution ^40^

## Code availability

CUDA C++ computer code developed for the study and analysis scripts for generating figures are available on GitHub repositories https://github.com/FrancisCrickInstitute/tumour-growth-patterns-impact-evolution and https://github.com/iamfuxiao/tumour-growth-patterns-impact-evolution ^40^.
