# Supplementary material for: Spatial patterns of tumour growth impact clonal diversification
Source: Nat Ecol Evol. Author manuscript; Available in PMC 2022 Jan 17. (PMC8752443; doi:10.1038/s41559-021-01586-x)
Supplement: Supplementary Information [file EMS136364-supplement-Supplementary_Information.pdf]

Supplementary Information Flat Files for:

Title:

Spatial patterns of tumour growth impact clonal diversification

Authors:

Xiao Fu[1,2*], Yue Zhao[3,4,5,6*], Jose I. Lopez[7], Andrew Rowan[3], Lewis Au[8,9], Annika Fendler[8], Steve Hazell[10], Hang Xu[3,11], Stuart Horswell[12], Scott T. C. Shepherd[8,9], Charlotte E. Spencer[8,9], Lavinia Spain[8,9], Fiona Byrne[8], Gordon Stamp[13], Tim O'Brien[14], David Nicol[15], Marcellus Augustine[3], Ashish Chandra[16], Sarah Rudman[17], Antonia Toncheva[18], Andrew J. S. Furness[8,9], Lisa Pickering[9], Santosh Kumar[19], Dow-Mu Koh[19,20], Christina Messiou[19,20], Derfel ap Dafydd[20], Matthew R. Orton[21], Simon J. Doran[19], James Larkin[9], Charles Swanton[3,4,22], Erik Sahai[2#], Kevin Litchfield[3,4#], Samra Turajlic[8,9#] on behalf of the TRACERx Renal Consortium, Paul A. Bates[1#]

1. Biomolecular Modelling Laboratory, The Francis Crick Institute, 1 Midland Rd, London NW1 1AT, UK

2. Tumour Cell Biology Laboratory, The Francis Crick Institute, 1 Midland Rd, London NW1 1AT, UK

3. Cancer Evolution and Genome Instability Laboratory, The Francis Crick Institute, 1 Midland Rd, London NW1 1AT, UK

4. Cancer Research UK Lung Cancer Centre of Excellence, University College London Cancer Institute, Paul O'Gorman Building, 72 Huntley Street, London, WC1E 6BT, UK

5. Department of Thoracic Surgery, Fudan University Shanghai Cancer Center, Shanghai, China. 200032.

6. Department of Oncology, Shanghai Medical College, Fudan University, Shanghai, China. 200032.

7. Department of Pathology, Cruces University Hospital, Biocruces-Bizkaia Institute, 48903 Barakaldo, Bizkaia, Spain

8. Cancer Dynamics Laboratory, The Francis Crick Institute, 1 Midland Rd, London NW1 1AT, UK

9. Renal and Skin Units, The Royal Marsden Hospital, London, SW3 6JJ, UK

10. Department of Pathology, the Royal Marsden NHS Foundation Trust, London SW3 6JJ, UK

11. Stanford Cancer Institute, Stanford University School of Medicine, Stanford, California, US

12. Department of Bioinformatics and Biostatistics, The Francis Crick Institute, 1 Midland Rd, London NW1 1AT, UK

13. Experimental Histopathology Laboratory, The Francis Crick Institute, London NW1 1AT, UK

14. Urology Centre, Guy's and St. Thomas' NHS Foundation Trust, London SE1 9RT, UK

15. Department of Urology, the Royal Marsden NHS Foundation Trust, London SW3 6JJ, UK

16. Department of Pathology, Guy's and St. Thomas NHS Foundation Trust, London SE1 9RT, UK

17. Department of Medical Oncology, Guy's and St. Thomas' NHS Foundation Trust, London SE1 9RT, UK

18. Biobank, Guy's and St. Thomas' NHS Foundation Trust, London SE1 7EH, UK

19. Division of Radiotherapy and Imaging, Institute of Cancer Research, 15 Cotswold Road, Sutton, Surrey, SM2 5NG

20. Department of Radiology, Royal Marsden Hospital, Fulham Rd, London, SW3 6JJ

21. Artificial Intelligence Imaging Hub, Royal Marsden NHS Foundation Trust, Sutton, UK

22. Department of Medical Oncology, University College London Hospitals, 235 Euston Rd, Fitzrovia, London, NW1 2BU, UK

Table of Contents

TITLE: **1**

AUTHORS: **1**

SUPPLEMENTARY NOTES **5**

SUPPLEMENTARY NOTE 1. ADDITIVE MODEL OF DRIVER ADVANTAGES. 5
SUPPLEMENTARY NOTE 2. GROWTH MODES IMPACT THE SPATIAL PATTERNS OF FITNESS. 6
SUPPLEMENTARY NOTE 3. SPATIAL HOMOGENENISATION OF SUBCLONES ABOLISHES CHARACTERISTIC PATTERNS OF MICRODIVERSITY HOTSPOTS. 7
SUPPLEMENTARY NOTE 4. SPATIAL FEATURES OF MICRODIVERSITY IN AN EXPANDED SET OF GROWTH MODELS. 8
SUPPLEMENTARY NOTE 5. SCALING FEATURES OF MICRODIVERSITY IN CCRCC TUMOURS ARE ASSOCIATED WITH CLINICAL BEHAVIOURS 9
SUPPLEMENTARY NOTE 6. FREQUENCY OF PARALLEL MUTATIONAL EVENTS IN CCRCCS 10
SUPPLEMENTARY NOTE 7. EVOLUTIONARY REPLAY *IN SILICO* SUGGESTS THAT BUDDING MAY INFORM EVOLUTIONARY TRAJECTORIES 11
SUPPLEMENTARY NOTE 8. CONSIDERATIONS FOR THE SELECTION OF DRIVER ACQUISITION RATES IN THE COARSE-GRAINED MODEL 12

SUPPLEMENTARY FIGURE LEGENDS **14**

SUPPLEMENTARY FIGURE 1. CLONAL DIVERSITY IN MODELS WITH SMALL DRIVER ACQUISITION PROBABILITIES ($pdriver$). 14
SUPPLEMENTARY FIGURE 2. CLONAL DIVERSITY IN MODELS WITH ZERO OR SMALL SELECTIVE COEFFICIENT (s). 14
SUPPLEMENTARY FIGURE 3. REPRESENTATIVE EXAMPLES OF SPATIAL MAPS OF SUBCLONES AND FITNESS UNDER VARIOUS MODEL CONDITIONS. 15
SUPPLEMENTARY FIGURE 4. SPATIAL FEATURES OF TUMOUR FITNESS IN MODELS WITH SATURATED DRIVER ADVANTAGES. 15
SUPPLEMENTARY FIGURE 5. SPATIAL FEATURES OF TUMOUR FITNESS IN MODELS WITH ADDITIVE DRIVER ADVANTAGES. 16
SUPPLEMENTARY FIGURE 6. QUANTILE-QUANTILE (Q-Q) PLOTS OF OBSERVED DISTRIBUTION OF MICRODIVERSITY HOTSPOTS VERSUS THE FITTED POWER LAW DISTRIBUTION. 16
SUPPLEMENTARY FIGURE 7. ASSOCIATION BETWEEN SPATIAL FEATURES OF MICRODIVERISTY AND CLINICAL BEHAVIOUR. 17
SUPPLEMENTARY FIGURE 8. PHOTOGRAPH AND HISTOLOGICAL IMAGES OF REPRESENTATIVE CASE K156. 17

Supplementary Notes

Supplementary Note 1. Additive model of driver advantages.

In the main text, we focused on presenting a saturated model of driver advantages, assuming that each of the ccRCC drivers endowed a tumour voxel with one of the three possible levels of growth probabilities and that, once acquiring one of the strongest drivers, the growth probability of a tumour voxel became saturated.

Separately, we evaluated an additive model of driver advantages (**Extended Data Figure 2a**, see Methods), assuming that acquisition of each ccRCC driver added to the growth probability of a tumour voxel and the relative selective advantages of drivers were defined according to their association with Ki67 score. Three scenarios were considered to reflect increasing amount of growth probabilities added by drivers on average (**Extended Data Figure 2b**).

For a qualitative comparison between two models of driver advantages, we focused on examining the clonal diversity observed at the end of simulation for conditions of driver acquisition probability highlighted for Surface Growth model ($p_{driver} = 2 \times 10^{-4}$) and for Volume Growth model ($p_{driver} = 1 \times 10^{-3}$) in the Main Text (**Main Figure 2**). Consistent with the finding discussed in the main text, for all of these scenarios, Volume Growth models (**Extended Data Figure 2c**), even with a greater p_{driver} employed, resulted in less extensive subclonal diversification than Surface Growth models (**Extended Data Figure 2d**). In both growth models, greater average growth probabilities added by drivers led to more extensive diversification. Additionally, in the Volume Growth model, clonal proportions across repeated simulations with the second scenrio (i.e., $\min(s_k) = 0.015$, $\Delta s_k = 0.005$) were similar to those highlighted in the Main Text (**Main Figure 2c (ii)**), suggesting a minimal

impact of the implementation of selective advantage on the observed clonal diversity at the end of simulation. In constrast, in the Surface Growth model, repeated simulations with the second scenario displayed a greater extent of subclonal diversification (i.e., smaller size of largest subclone “subclone 1”) than those highlighted in the Main Text (**Main Figure 2c (iii)**), suggesting that Surface Growth mode could be more sensitive to the implementation of selective advantage conferred by drivers, with greater extent of diversification in additive model of driver advantages.

Supplementary Note 2. Growth modes impact the spatial patterns of fitness.

In the main text, we presented the impact of growth modes on spatial patterns of fitness (**Main Figure 2**). Here, we discuss this finding in greater depth. In the model, we defined the “fitness” of a tumour voxel as its growth probability dependent on the list of drivers it harbours (See Methods). Then the fitness of each tumour voxel within a tumour slice was mapped (**Supplementary Figure 3**). In Surface Growth models with saturated driver advantages, bulging regions along the tumour contour were represented by outgrowing subclones with much greater fitness than adjacent tumour areas, while in those with additive driver advantages, many subclones, with small differences in fitness, coexisted at the tumour frontier. By contrast, Volume Growth models with saturated driver advantages showed few subclones with high fitness, while those with additive driver advantages generally lacked subclones with high fitness. With the incorporation of necrosis, less fit clones became extinct while fitter clones were selected to dominate at the tumour centre, in keeping with our recent study (Zhao Y., *et al. Nat. Ecol. Evol.* (2021)).

The spatial features of fitness were quantitatively analysed by taking samples from the simulated tumours (see Methods; **Supplementary Figure 4-5**). For Volume Growth models, the presence of necrosis generally enhanced the fitness in both tumour margin and centre, with a pronounced effect in models with implementation of saturated driver advantage. For Surface Growth models, the tumour margin showed high fitness and was not impacted by necrosis; however, the fitness of the tumour centre was dramatically enhanced when necrosis was incorporated.

Collectively, we found that necrosis in the simulated tumours could lead to the selection of fitter subclones with greater growth probabilities in the tumour centre, consistent with our experimental observation that aggressive subclones were enriched in the tumour centre (Zhao Y., *et al. Nat. Ecol. Evol.* (2021)).

Supplementary Note 3. Spatial homogenenisation of subclones abolishes characteristic patterns of microdiversity hotspots.

In the main text, we presented that microdiversity hotspots were increasingly frequent towards the tumour margin and the cumulative probablility distribution follows power law scaling (**Main Figure 3**). Importantly, the observed power law distribution exhibited an exponent much greater than the value as would be expected from a uniform spatial distribution in a circular area in two dimensions (2D) (**Extended Data Figure 5a-b**), suggesting a preferential enrichment of microdiversity hotspots at the margin.

Here, we present additional analyses supporting that the spatial organisation of subclones underlied the observed chracterisatic scaling patterns of microdiversity hotspots. Briefly, we spatially homogenenised the patterns of subclones within the a tumour slice. Importantly, the proportions of subclones in a tumour slice were kept unchanged but the spatial organisation

of subclones was entirely lost (**Extended Data Figure 5c-d**). Interestingly, this spatial homogenenisation was sufficient to reduce the scaling exponent to approximately 2, as would be expected via random sampling of spots from a 2D circular area (**Extended Data Figure 5e-f**). Collectively, these data together with analyses presented in the main text (**Main Figure 4**) suggested the importance of the emergent spatial organisation of subclones, as a consequence of tumour growth in a spatial context, in shaping the scaling patterns of microdiversity hotspots.

Supplementary Note 4. Spatial features of microdiversity in an expanded set of growth models.

In the main text, we presented the spatial patterns of microdiversity in Surface and Volume Growth models with saturated model of driver advantages in the absence of necrosis (**Main Figure 3**).

Here, we further discuss the spatial patterns of microdiversity in an expanded set of growth models, considering the implementation of additive driver advantages and incorporation of necrosis (see Methods; **Extended Data Figure 6**). Models with different implementations of driver advantages showed similar profiles of probability density distributions characterising the spatial locations of microdiversity hotspots, in the absence of necrosis. With necrosis incorporated, microdiversity hotspots were largely enriched in the non-necrotic tumour margin. However, Surface Growth models, especially those with saturated driver advantages, clearly showed a bi-modal probability density distribution indicating the enrichment of additional microdiversity hotspots at the necrotic tumour centre. This finding was in accordance with our recent finding that harsher environments at the tumour interior could

select aggressive clones and potentiate continual subclonal diversification (Zhao Y. *et al. Nat. Ecol. Evol.* (2021)).

Supplementary Note 5. Scaling features of microdiversity in ccRCC tumours are associated with clinical behaviours.

In the main text, we presented that, corroborating the modelling observations, 606 regions with at least 2 clones from 54 tumours, defined as microdiversity hotspots in the tumour data, were increasingly frequent towards the tumour margin (**Main Figure 3**). Furthermore, the cumulative probability distribution characterising the spatial locations of these regions followed a power law distribution, consistent with the model, and the exponent of the power law was associated with clinical behaviours, based on previously published annotations (Turajlic S., *et al. Cell* (2018), **Supplementary Table 2**).

Here, we present the analysis that established this association. When the 54 tumours were split into two subsets according to whether the patient has relapsed (270 tumour regions) or not (336 tumour regions), the subset where the patient has relapsed showed a significantly steeper gradient of spatially distributed microdiversity hotspots (i.e., larger power law exponent) than the subset where the patient hasn't (**Supplementary Figure 7**). Additionally, when the 54 tumours are split into three subsets according to the rates of disease progression – attenuated progression (265 tumour regions), rapid progression (65 tumour regions), and no progression (276 tumour regions), the subset with attenuated progression showed the steepest gradient of spatial distribution of microdiversity hotspots, while subsets with either lack of or rapid progression showed shallow gradient (**Supplementary Figure 7**).

These data suggested that tumours mapped to a poorer clinical outcome are typically associated with a steeper spatial distribution of microdiversity hotspots and enrichment towards the tumour margin. Furthermore, together with the macrodiversity and

microdiversity analysis in the simulated tumours (**Main Figures 2-3**), we conclude that different growth modes correspond to tumours with distinct patterns of evolution and progression. Surface Growth models, showing enrichment microdiversity hotspots towards the margin, are mapped to tumours with branched evolution and attenuated progression; Volume Growth models, showing more uniform distribution of microdiversity hospots, are mapped to either indolent tumours with lack of evolution and progression or aggression tumours with punctuated evolution and rapid progression.

Supplementary Note 6. Frequency of parallel mutational events in ccRCCs

In the main text, we presented the spatial patterns of parallel mutational events with limited clonal expansion (**Main Figure 4**). Here, we also briefly report the frequency of parallel evolution events in the cohort.

The observation of parallel evolution in ccRCCs was reported previously (Turajlic S., *et al. Cell* (2018)). Due to the limits of spatial sampling and sequencing, parallel gene mutations were detected in 28 out of 66 tumours (**Supplementary Table 3**). A total of 71 of 114 parallel events in 18 tumours were alterations in known ccRCC drivers, including *ARID1A*, *BAP1*, *KDM5C*, *PBRM1*, *PTEN*, *SETD2*, and *VHL*. Among these drivers, parallel evolution of alterations in *PBRM1*, *SETD2*, and *BAP1* were most frequent, in 6, 5, and 4 tumours, respectively. Consistent with previous observations (Turajlic S., *et al. Cell* (2018)), parallel mutational events could span a variable number of patient tumour (PT) regions. For example, in K520 (**Main Figure 5**), multiple parallel events of *PBRM1* mutations co-existed, with one highly prevalent event spanning more than 10 PT regions and additional events displaying less clonal expansion.

Supplementary Note 7. Evolutionary replay *in silico* suggests that budding may inform evolutionary trajectories

In the main text, we presented that the appearance of budding structures in Surface Growth models preceded the subsequent subclonal outgrowth and diversification (**Main Figure 5**). This suggested that the evolutionary trajectories could become more constrained after the the emergence of budding structures. Here, we explored this by performing evolutionary replay simulations.

Specifically, an *in-silico* tumour under Surface Growth was prepared for evolutionary replay (**Extended Data Figure 9, Extended Data Figure 10a**). Using the historical state of this tumour at a particular time point as a common starting state, 50 new *in-silico* tumours were simulated (**Extended Data Figure 10b**). While re-grown tumours starting from earlier historical states displayed markedly divergent patterns of subclones in the end (**Extended Data Figure 10c (i)**), those grown from historical states collected from later stages, especially after the emergence of the budding structure, appeared very similar to the original pattern of subclones (**Extended Data Figure 10c (ii-iv)**). Quantitatively, this was evidenced by a decreasing divergence in Shannon diversity at the end of simulations after evolutionary replay as a function of the size of the starting tumour state (**Extended Data Figure 10d**). Similarly, this trend of decreasing divergence was noted in *in-silico* tumours under Surface Growth with a greater probability of driver acquisition (**Extended Data Figure 10e**) as well as those under Volume Growth (**Extended Data Figure 10f**). This finding suggested that budding structures in a tumour under Surface Growth could indicate future evolutionary trajectories.

Supplementary Note 8. Considerations for the selection of driver acquisition rates in the coarse-grained model

For a number of reasons, we believe that choosing an exact driver acquisition rate in this coarse-grained model is fundamentally difficult and may be unnecessary for our aim of study:

Firstly, and most importantly, employing a mutation rate based on a “macroscopic” metric of evolutionary outcome via inference (e.g., as in Williams et al. (2016) Nature Genetics) may well lead to the risk of generating circular arguments in our study. As discussed in other parts of the manuscript (e.g., **Main Figure 5, Extended Data Figure 8**), Volume Growth and Surface Growth models show very different time scales of growth and spatial extent and uniformity of driver accumulation. These two models would apparently differ in the inferred mutation rates given the same “macroscopic” metric. For example, to achieve the same Shannon diversity at the end, it requires a much higher driver acquisition rate for Volume Growth than for Surface Growth (**Main Figure 2e**). Thus, we chose to contrast two models always at the same driver acquisition rate, for a range of values, and with the same implementation of driver advantages.

Secondly, a mapping relationship from mutation rate per cell division to the effective mutation rate at the tumour voxel level is lacking and would be an interesting question for future study. With our coarse-grained approach, we focused on large-scale clonal dynamics and therefore neglected finer-scale clonal dynamics within each tumour voxel as well as the impact of cell migratory dynamics, which will be needed for establishing a mapping relationship. Recent work started to shed lights on this question by simulating clonal dynamics under domains with varying sizes and found that the spatial constraint could influence the type of realised evolution. i.e., neutral vs. Darwinian evolution (West et al Nature Communications (2021)).

Thirdly, the ways to collecting samples could make inference of evolutionary parameters difficult. As demonstrated in Chkhaidze et al PLoS Comput Biol. (2019), spatial tumour growth and specific sampling procedures could influence the inferred type of evolution. Given that in the TRACERx Renal cohort, the number and spatial distribution of regional samples vary among tumours, it's difficult to dissect the difference in mutation rates from these factors.

With all the considerations above, we decided to examine our model outputs across a range of driver acquisition rates and evaluate rates that range from very small value (**Supplementary Figure 1**) where both growth models lack macrodiversity to large value where both growth models show high macrodiversity. While this choice doesn't inform us of the mutation rates in ccRCCs, which is not the focus of our study, we are able to consistently contrast outcomes in clonal diversification between Volume Growth and Surface Growth models at the same driver acquisition rate, across a wide range of values.

Supplementary Figure Legends

Supplementary Figure 1. Clonal diversity in models with small driver acquisition probabilities (p_{driver}).

(a-b) Whole-tumour CCF of parental and largest subclones in *in-silico* tumours under Surface Growth (a) and Volume Growth (b), respectively, under the indicated parameter conditions. "Parental (3p loss, *VHL*)" clone is shown along with up to five subclones with a whole-tumour CCF of 0.01 or higher. All remaining subclones are represented in the "other" group.

(d) Whole-tumour CCF of parental clone in *in-silico* tumours under Volume Growth and Surface Growth with varying driver acquisition probabilities. N = 100 for each condition.

(e) Shannon diversity index in *in-silico* tumours under Volume Growth and Surface Growth with varying driver acquisition probabilities. N = 100 for each condition.

Statistical annotations in (d-e) reflect two-sided Wilcoxon tests: "****" indicates $P \leq 0.0001$.

Supplementary Figure 2. Clonal diversity in models with zero or small selective coefficient (s).

(a-b) Reproduced from **Main Figure 2a-b** for reference to parameter domains. (a) Schematic figure for the whole-tumour analysis of clonal diversity. (b) Heatmap showing the average number of clones (i.e., parental clone and subclones) with respect to driver acquisition probability and selective coefficient in the Volume Growth (i) and Surface Growth (ii) models.

The average is calculated from 50 *in-silico* tumours per parameter condition. Clones with a whole-tumour CCF of at least 0.05 are counted for this analysis.

(c-d) Whole-tumour CCF of parental clone and largest subclones in *in-silico* tumours under Surface Growth and Volume Growth, respectively, under the indicated parameter conditions. "Parental (3p loss, *VHL*)" clone is shown along with up to five subclones with a whole-tumour CCF of 0.01 or higher. All remaining subclones are represented in the "other" group.

Supplementary Figure 3. Representative examples of spatial maps of subclones and fitness under various model conditions.

Implementations without or with necrosis are shown at top or bottom, respectively. Subclones are shown in randomly generated colours, while parental clone is shown in grey in spatial maps of subclones. Higher fitness values are reflected by greater intensities of purple. Driver acquisition probabilities in these representative simulations are $p_{driver} = 2 \times 10^{-4}$.

Supplementary Figure 4. Spatial features of tumour fitness in models with saturated driver advantages.

(a) Mean fitness of marginal-most (10%) of tumour voxels. (b) Mean fitness of central-most (10%) tumour voxels. (c) Ratio of the mean fitness of central-most (10%) tumour voxels to that of marginal-most (10%) tumour voxels ("Ratio_C2M"). Panels (i)-(iii) in (a-c) reflect runs with varying driver acquisition probabilities. (d) Mean fitness of randomly sampled (10%) tumour voxels against the Ratio_C2M. Data points in (d) reflect sets of simulations

with varying growth patterns (colour), driver acquisition rates (size), and implementation of necrosis (symbol). N = 50 simulations for each condition.

Supplementary Figure 5. Spatial features of tumour fitness in models with additive driver advantages.

(a) Mean fitness of marginal-most (10%) tumour voxels. (b) Mean fitness of central-most (10%) of tumour voxels. (c) Ratio of mean fitness of central-most (10%) tumour voxels to that of marginal-most (10%) tumour voxels ("Ratio_C2M"). Panels (i)-(iii) in (a-c) reflect runs with varying driver acquisition probabilities. (d) Mean fitness of randomly sampled (10%) tumour voxels against the Ratio_C2M. Data points in (d) reflect sets of simulations with varying growth patterns (colour), driver acquisition rates (size), and implementation of necrosis (symbol). N = 50 simulations for each condition.

Supplementary Figure 6. Quantile-Quantile (Q-Q) plots of observed distribution of microdiversity hotspots versus the fitted power law distribution.

From left to right represent Surface Growth Model, Volume Growth Model and experimental data, with conditions indicated within figures. "S" and "V" in the figure reflect Surface Growth and Volume Growth, respectively. "p=2e-4" reflects a driver acquisition probability of 2e-4. In addition, the median fitted power law exponent k, as in $P(D \leq d) \sim d^k$, from bootstrapping (in **Main Figure 3f-g**) is indicated within figures.

Supplementary Figure 7. Association between spatial features of microdiveristy and clinical behaviour.

(a-b) Cumulative probability distribution, $P(D \leq d)$, of the normalised distance to tumour centre in ccRCC tumours, split into subsets according to either relapse status (a) or rates of disease progression (b). 606 patient tumour (PT) regions from 54 ccRCC tumours are considered for this analysis ("complete.set"). Subsets with different relapse statuses consist of 270 ("relapse") and 336 regions ("no.relapse"), respectively. Subsets with different rates of disease progression consist of 276 ("no.progression"), 265 ("attenuated.progression"), and 65 regions ("rapid.progression"), respectively.

(c-d) Bootstrapped power law exponent k, as in $P(D \leq d) \sim d^k$, fitted to cumulative probability distribution of normalised distance to tumour centre in each of bootstrap samples, in subsets of RCC tumours.

(e) Quantile-Quantile (Q-Q) plots of observed versus fitted distributions of microdiversity hotspots in subsets of RCC tumours.

Statistical annotations in (c-d) reflect two-sided Wilcoxon tests: "****" indicates $P \leq 0.0001$.

Supplementary Figure 8. Photograph and histological images of representative case K156.

Photograph of the ex-vivo tumour (top left) and histological images of regional biopsies (R21, R36, R37, and R38) are presented.

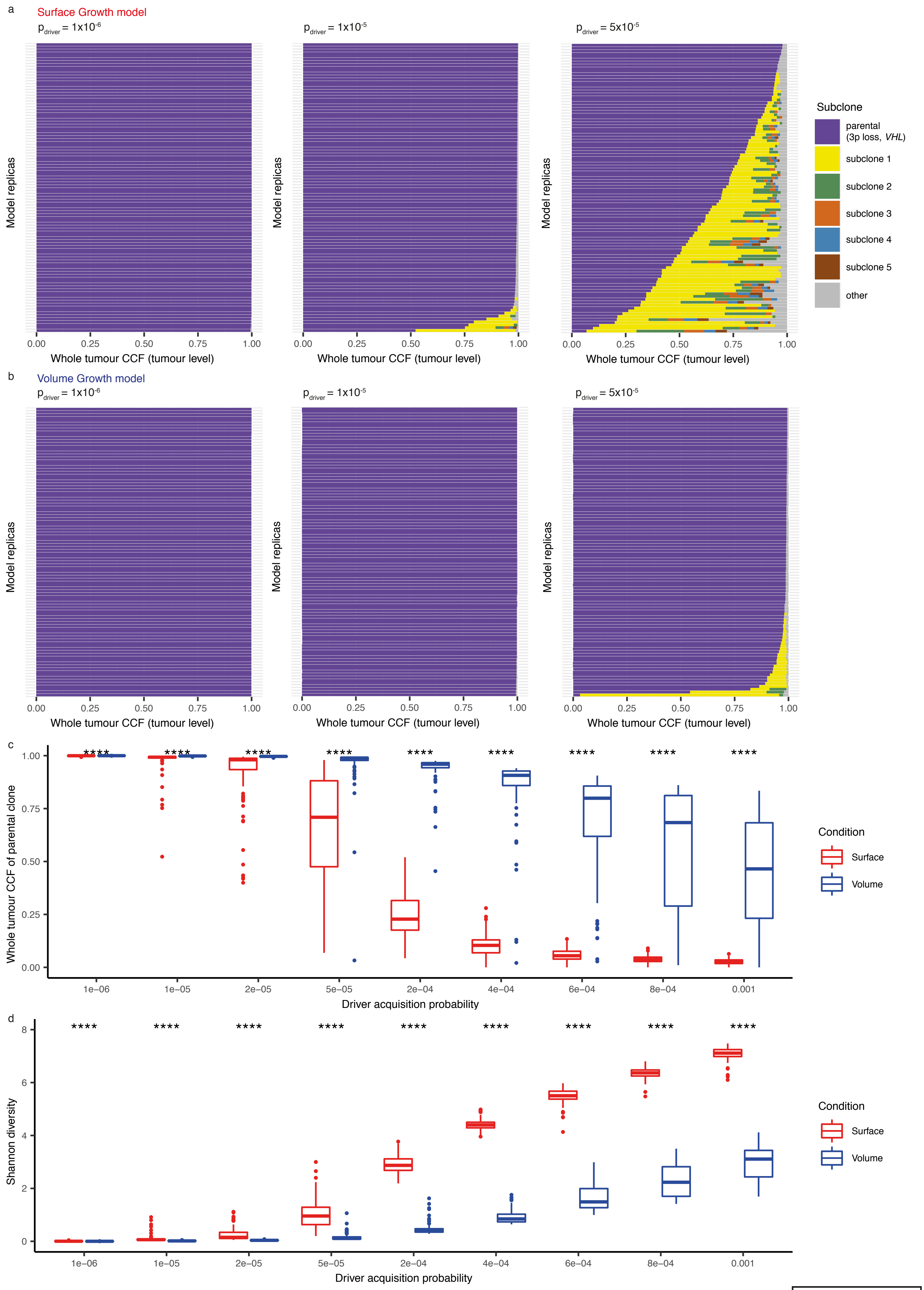

Supplemental Figure 1

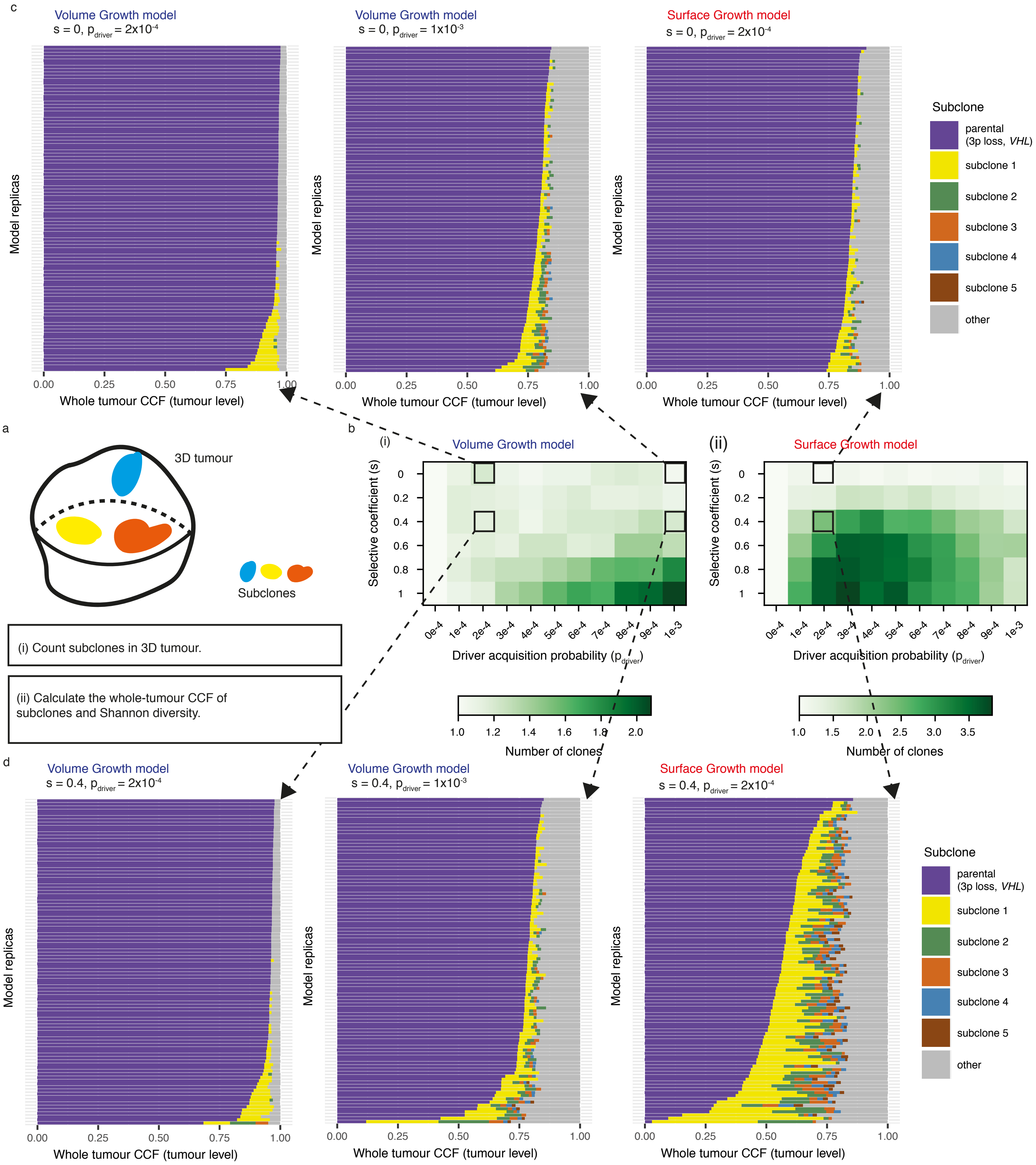

c
Volume Growth model
s = 0, p_{driver} = 2x10^{-4}
Volume Growth model
s = 0, p_{driver} = 1x10^{-3}
Surface Growth model
s = 0, p_{driver} = 2x10^{-4}
Model replicas
Whole tumour CCF (tumour level)
0.00
0.25
0.50
0.75
1.00
Subclone
parental (3p loss, *VHL*)
subclone 1
subclone 2
subclone 3
subclone 4
subclone 5
other
a
3D tumour
Subclones
(i) Count subclones in 3D tumour.
(ii) Calculate the whole-tumour CCF of subclones and Shannon diversity.
b
(i)
Volume Growth model
(ii)
Surface Growth model
Selective coefficient (s)
0
0.2
0.4
0.6
0.8
1
0e-4
1e-4
2e-4
3e-4
4e-4
5e-4
6e-4
7e-4
8e-4
9e-4
1e-3
Driver acquisition probability (p_{driver})
1.0
1.2
1.4
1.6
1.8
2.0
1.5
2.5
3.0
3.5
Number of clones
d
Volume Growth model
s = 0.4, p_{driver} = 2x10^{-4}
Volume Growth model
s = 0.4, p_{driver} = 1x10^{-3}
Surface Growth model
s = 0.4, p_{driver} = 2x10^{-4}

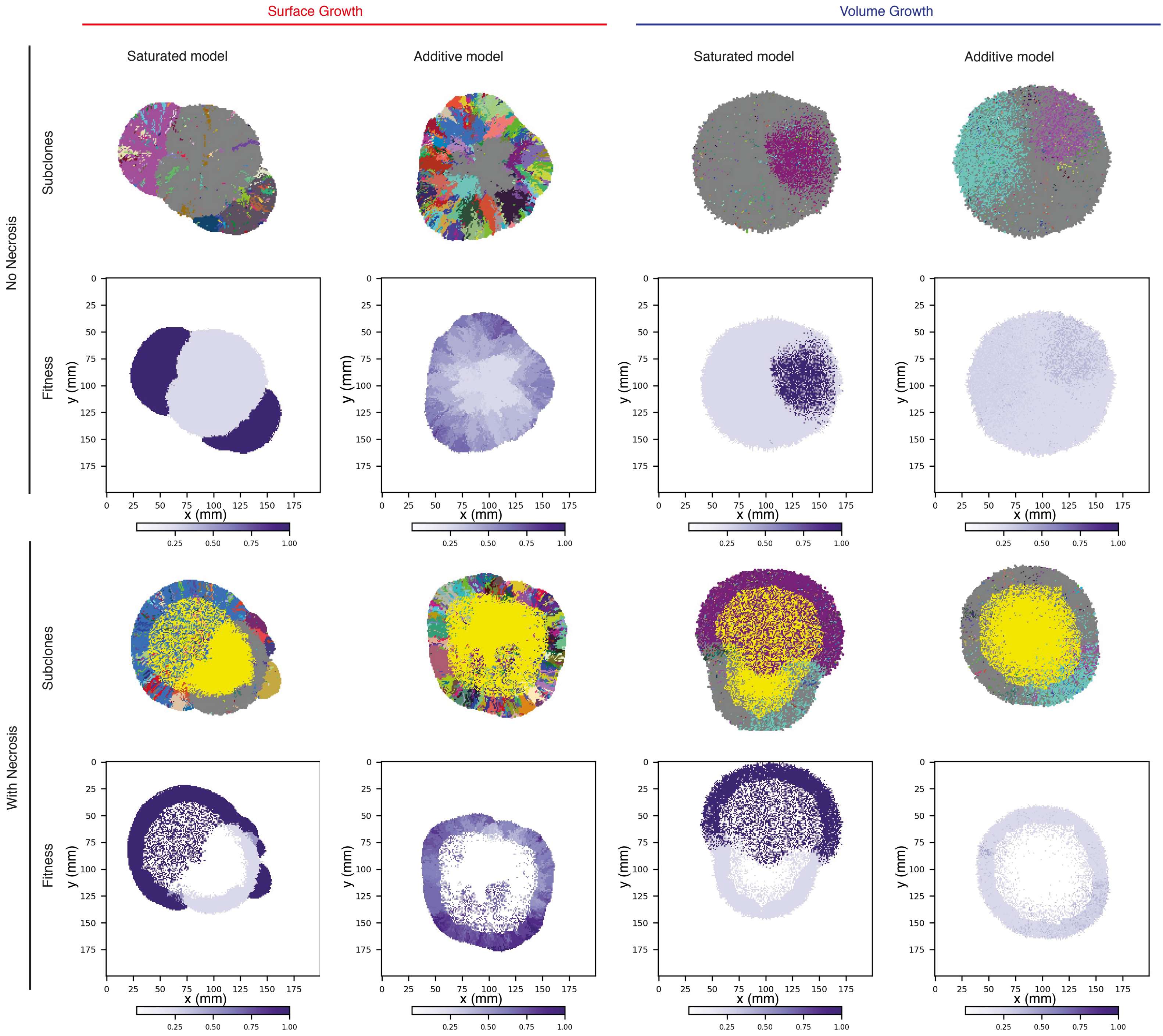

Supplemental Figure 3

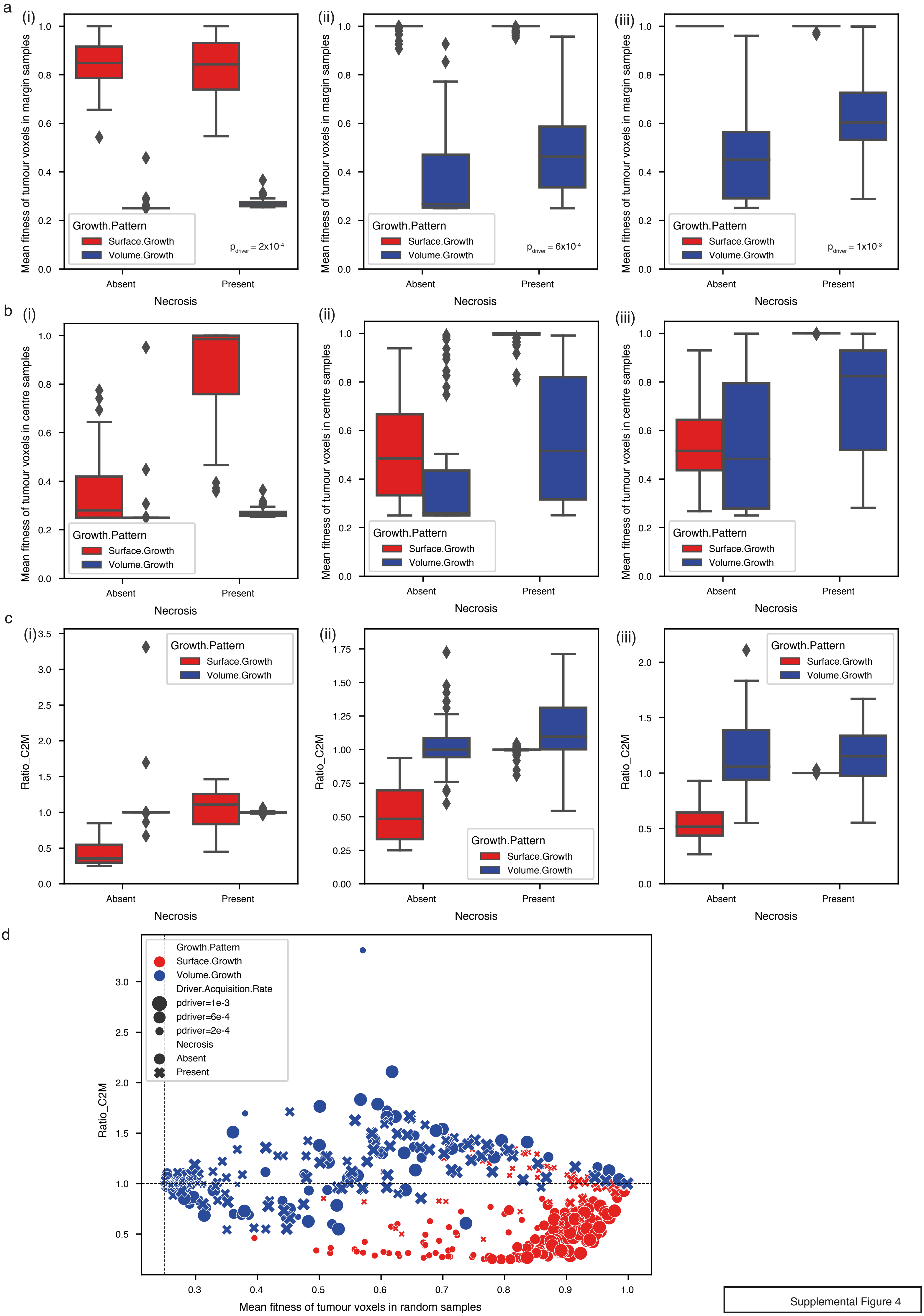

Supplemental Figure 4

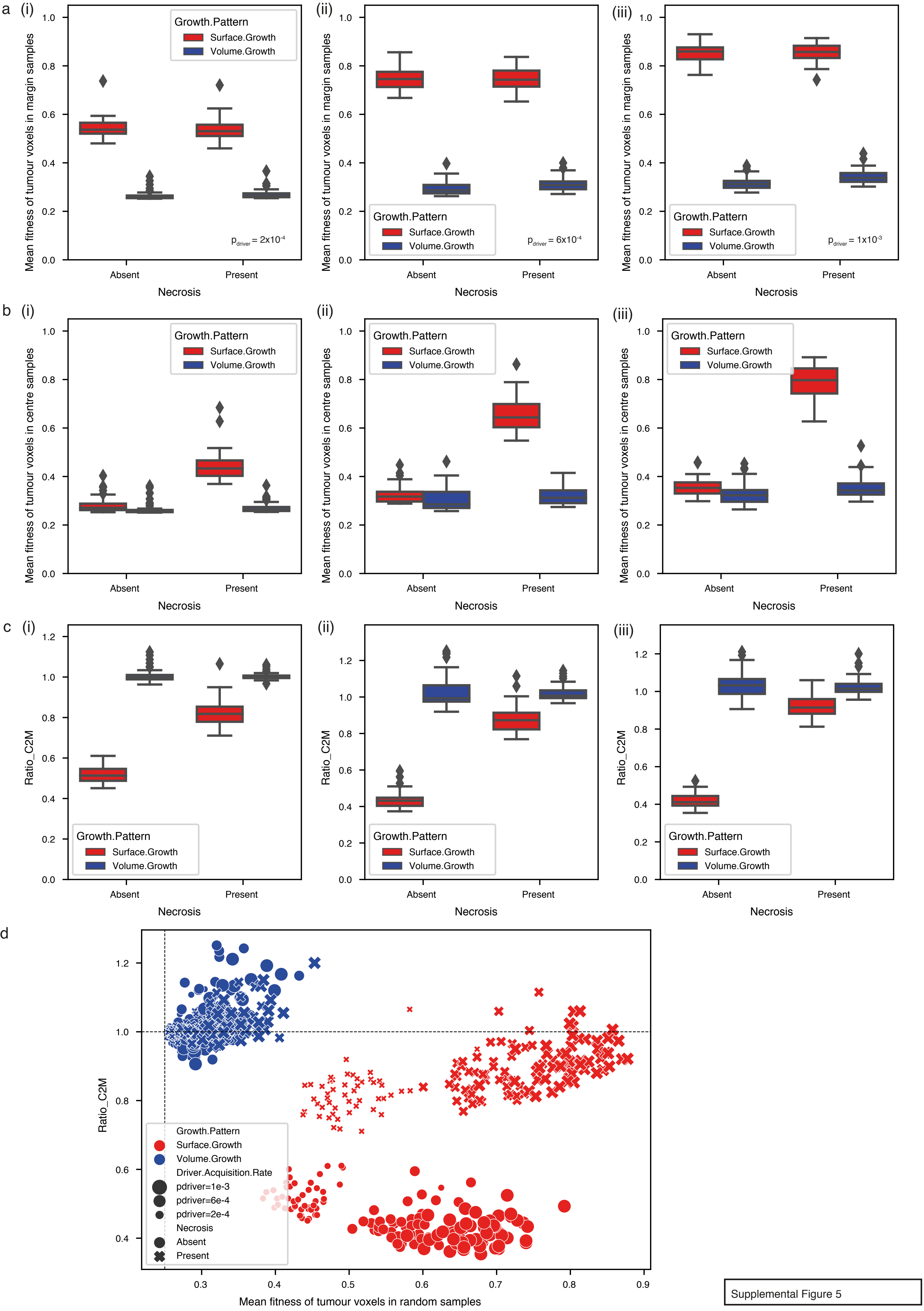

Supplemental Figure 5

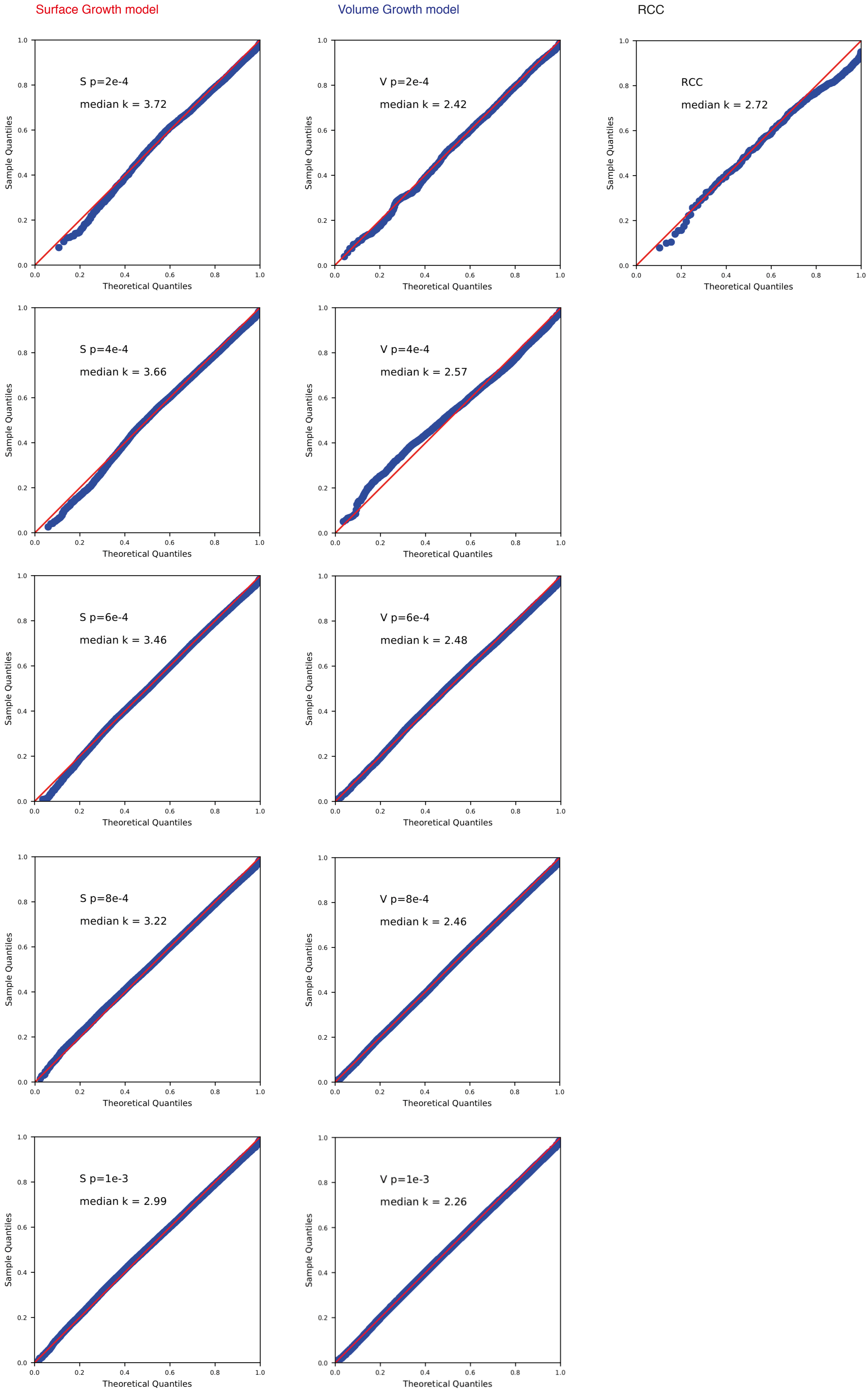

Supplemental Figure 6

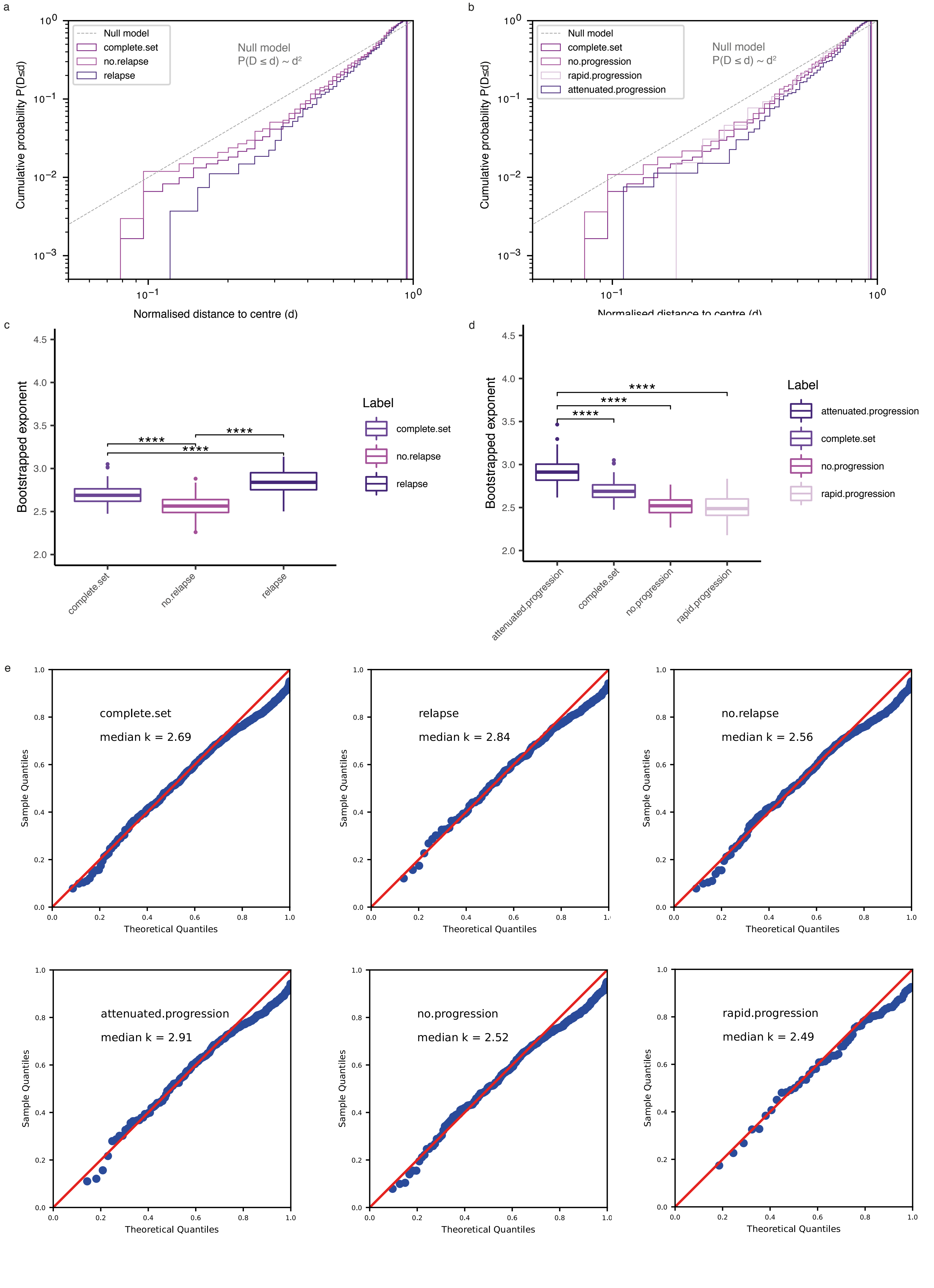

Supplemental Figure 7

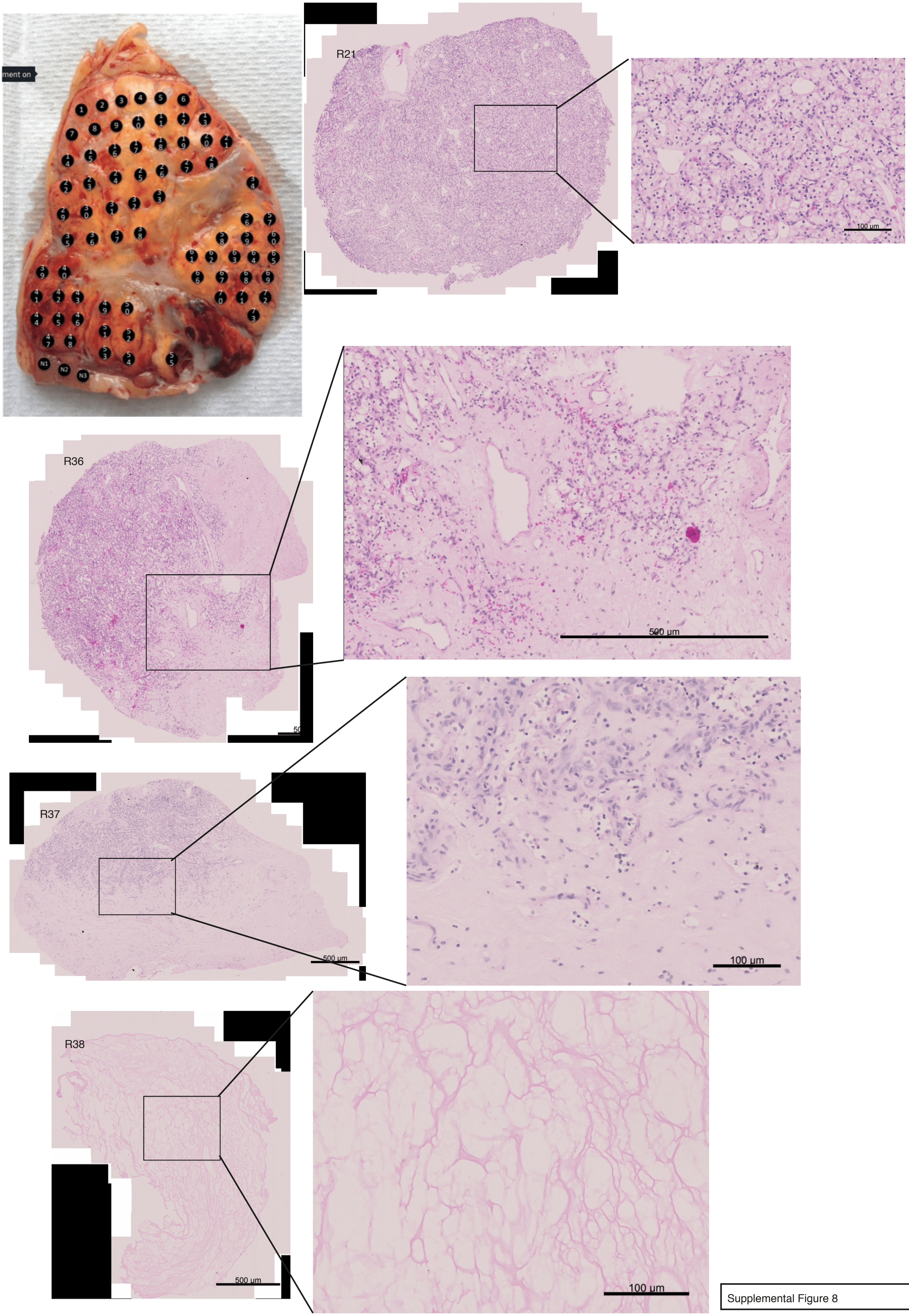

Supplemental Figure 8
