# Supplementary material for: Spatial patterns of tumour growth impact clonal diversification in a computational model and the TRACERx Renal study
Source: Nat Ecol Evol. 2021 Dec 23;6(1):88–102. doi: 10.1038/s41559-021-01586-x (PMC8752443; doi:10.1038/s41559-021-01586-x)
Supplement: Supplementary file 2 [file 41559_2021_1586_MOESM2_ESM.pdf]

nature portfolio

Corresponding author(s): Erik Sahai, Kevin Litchfield, Samra Turajlic, Paul A Bates

Last updated by author(s): Sep 27, 2021

Reporting Summary

Nature Portfolio wishes to improve the reproducibility of the work that we publish. This form provides structure for consistency and transparency in reporting. For further information on Nature Portfolio policies, see our Editorial Policies and the Editorial Policy Checklist.

Statistics

For all statistical analyses, confirm that the following items are present in the figure legend, table legend, main text, or Methods section.

n/a	Confirmed	
☐	☒	The exact sample size (*n*) for each experimental group/condition, given as a discrete number and unit of measurement
☐	☒	A statement on whether measurements were taken from distinct samples or whether the same sample was measured repeatedly
☐	☒	The statistical test(s) used AND whether they are one- or two-sided<br>*Only common tests should be described solely by name; describe more complex techniques in the Methods section.*
☒	☐	A description of all covariates tested
☒	☐	A description of any assumptions or corrections, such as tests of normality and adjustment for multiple comparisons
☐	☒	A full description of the statistical parameters including central tendency (e.g. means) or other basic estimates (e.g. regression coefficient) AND variation (e.g. standard deviation) or associated estimates of uncertainty (e.g. confidence intervals)
☐	☒	For null hypothesis testing, the test statistic (e.g. *F*, *t*, *r*) with confidence intervals, effect sizes, degrees of freedom and *P* value noted<br>*Give P values as exact values whenever suitable.*
☒	☐	For Bayesian analysis, information on the choice of priors and Markov chain Monte Carlo settings
☒	☐	For hierarchical and complex designs, identification of the appropriate level for tests and full reporting of outcomes
☒	☐	Estimates of effect sizes (e.g. Cohen's *d*, Pearson's *r*), indicating how they were calculated

Our web collection on statistics for biologists contains articles on many of the points above.

Software and code

Policy information about availability of computer code

Data collection	The code for simulation was written in CUDA C++, where Cmake (version 3.12.1) and Cuda compilation tools (release 9.2) were used. This code is used to generate simulated data. Contrast-enhanced CT images were obtained using standard-of-care imaging sequences in 91 patients and curated using a local research PACS (based on the XNAT platform, (Marcus, et al., 2007)).
Data analysis	WebPlotDigitizer (version 4.3) was used to extract X- and Y-coordinates of the macroscopic photos. R (version 3.6.2) was used for generating tumour maps and statistical tests and plotting. Python (version 3.7) and R (version 3.6.2) were used for analysis of simulations. Adobe Illustrator (version 25.1) was used for combining the figure panels. Digital images of Ki67 immunohistochemical staining were analyzed using StrataQuest version 5 (TissueGnostics, Vienna, Austria) for Ki67 quantification.

For manuscripts utilizing custom algorithms or software that are central to the research but not yet described in published literature, software must be made available to editors and reviewers. We strongly encourage code deposition in a community repository (e.g. GitHub). See the Nature Portfolio guidelines for submitting code & software for further information.

Data

Policy information about availability of data

All manuscripts must include a data availability statement. This statement should provide the following information, where applicable:

- Accession codes, unique identifiers, or web links for publicly available datasets
- A description of any restrictions on data availability
- For clinical datasets or third party data, please ensure that the statement adheres to our policy

Sequencing data that supports this study have been deposited at the European Genome-phenome Archive (EGA), which is hosted by the European Bioinformatics Institute (EBI); accession number EGAS00001002793. Source Data and analysis for generating plots are available in a github repository.

Field-specific reporting

Please select the one below that is the best fit for your research. If you are not sure, read the appropriate sections before making your selection.

☒ Life sciences ☐ Behavioural & social sciences ☐ Ecological, evolutionary & environmental sciences

For a reference copy of the document with all sections, see nature.com/documents/nr-reporting-summary-flat.pdf

Life sciences study design

All studies must disclose on these points even when the disclosure is negative.

Sample size	Estimated enrollment of TRACERx renal trial (NCT03226886): 360. Images were taken for the first 100 patients to match up previously published data.
Data exclusions	Of the 102 macroscopic photos taken, cases in which the whole tumour couldn't be imaged to high quality (n = 9), without a clearly defined boundary between tumour and normal tissues (n = 5), or without exact positions of the biopsy regions (n = 9) were excluded. In total, 79 tumour sections of 66 unique primary tumours were included in this study.
Replication	NA
Randomization	NA; observational study.
Blinding	NA; observational study.

Reporting for specific materials, systems and methods

We require information from authors about some types of materials, experimental systems and methods used in many studies. Here, indicate whether each material, system or method listed is relevant to your study. If you are not sure if a list item applies to your research, read the appropriate section before selecting a response.

Materials & experimental systems

n/a	Involved in the study	
☐	☒	Antibodies
☒	☐	Eukaryotic cell lines
☒	☐	Palaeontology and archaeology
☒	☐	Animals and other organisms
☐	☒	Human research participants
☐	☒	Clinical data
☒	☐	Dual use research of concern

Methods

n/a	Involved in the study	
☒	☐	ChIP-seq
☒	☐	Flow cytometry
☒	☐	MRI-based neuroimaging

Antibodies

Antibodies used	Primary antibody used for immunohistochemical staining for Ki67 was rabbit anti-Ki67 (AB16667, Abcam, Cambridge, UK) and secondary antibody was Discovery Omnimap anti-rabbit HRP RUO (760-4311, Roche, Rotkreuz, Switzerland). DAB kit was Discovery Chromomap DAB RUO (760-4311, Roche).
Validation	Validation of rabbit anti-Ki67 (AB16667, Abcam, Cambridge, UK): knockout validation. Reference: Sobecki M, Mrouj K, Camasses A, Parisis N, Nicolas E, LlÃ¨res D, et al. The cell proliferation antigen Ki-67 organises heterochromatin. elife. 2016;5:e13722.

Human research participants

Policy information about studies involving human research participants

Population characteristics	Of the 101 TRACERx renal clear-cell renal cell carcinoma cases, there were 68 males and 33 females, with the median age of 64 (range: 34 - 84).
Recruitment	TRACERx Renal Inclusion Criteria:<br>1) Age 18- years or older<br>2) Patients with histopathologically confirmed renal cell carcinoma, or suspected renal cell carcinoma, proceeding to neoadjuvant therapy and/or nephrectomy/metastasectomy, or identified as having progressive disease<br>3) Or in patients undergoing nephrectomy for non-malignant disease<br>4) Medical and/or surgical management in accordance with national and/or local guidelines

	5) Written informed consent<br>Exclusion Criteria:<br>1) Any concomitant medical or psychiatric problems which, in the opinion of the investigator, would prevent completion of treatment or follow-up<br>2) Lack of adequate tissue
Ethics oversight	Royal Marsden NHS Foundation Trust

Note that full information on the approval of the study protocol must also be provided in the manuscript.

Clinical data

Policy information about clinical studies

All manuscripts should comply with the ICMJE guidelines for publication of clinical research and a completed CONSORT checklist must be included with all submissions.

Clinical trial registration	NCT03226886
Study protocol	Details of the TRACERx Renal clinical study can be found here: https://clinicaltrials.gov/ct2/show/study/NCT03226886.
Data collection	Clinical, genomic and follow-up data will be collected between July 6, 2017 and September 1, 2023 (final data collection date for primary outcome measure).
Outcomes	Primary Endpoint: to validate ITH index and WGII as stage and grade independent prognostic markers of progression free survival in patients with ccRCC mutation in a gene of interest.
