# Supplementary material for: Spatial patterns of tumour growth impact clonal diversification in a computational model and the TRACERx Renal study
Source: Nat Ecol Evol. 2021 Dec 23;6(1):88–102. doi: 10.1038/s41559-021-01586-x (PMC8752443; doi:10.1038/s41559-021-01586-x)
Supplement: Supplementary file 3 [file 41559_2021_1586_MOESM4_ESM.pdf]

natureresearch

Peer Review Information

Journal: Nature Ecology & Evolution
Manuscript Title: Spatial patterns of tumour growth impact clonal diversification: computational modelling and evidence in the TRACERx Renal study
Corresponding author name(s): Xiao Fu, Yue Zhao

Editorial Notes:

**Redactions – published data**	Parts of this Peer Review File have been redacted as indicated to remove third-party material.

Reviewer Comments & Decisions:

Decision Letter, initial version:

25th February 2021

*Please ensure you delete the link to your author homepage in this e-mail if you wish to forward it to your co-authors.

Dear Dr Bates,

Many thanks for submitting your manuscript entitled "Spatial patterns of tumour growth impact clonal diversification: computational modelling and evidence in the TRACERx Renal study" to Nature Ecology & Evolution. I have now discussed it with my editorial colleagues and we are in principle interested in sending it for peer review. However, we would first like to ask you to make some revisions in order to explain more explicitly the links between it and the linked manuscript NATECOLEVOL-200711078B that has just been accepted for publication. The reviewers will likely be an overlapping panel with those who reviewed that manuscript, and if published, readers will read them as a pair. As currently written, we felt the current submission does not reference the other manuscript sufficiently, and in particular has the potential to confuse as to whether evolution in the tumour centre or the tumour margin is most important. We don't feel that any major restructuring is necessary, but a clearer discussion of the link between the two studies and the similarities and differences would, we think, be of benefit. I hope that sounds reasonable - please let me know if it would be useful to discuss this in more detail.

Please use the link below to submit your revised manuscript and related files:

[REDACTED]

Note: This URL links to your confidential home page and associated information about manuscripts you may have submitted, or that you are reviewing for us. If you wish to forward this email to co-authors, please delete the link to your homepage.

Nature Ecology & Evolution is committed to improving transparency in authorship. As part of our efforts in this direction, we are now requesting that all authors identified as 'corresponding author' on published papers create and link their Open Researcher and Contributor Identifier (ORCID) with their account on the Manuscript Tracking System (MTS), prior to acceptance. ORCID helps the scientific community achieve unambiguous attribution of all scholarly contributions. You can create and link your ORCID from the home page of the MTS by clicking on 'Modify my Springer Nature account'. For more information please visit please visit www.springernature.com/orcid.

Please do not hesitate to contact me if you have any questions or would like to discuss these revisions further.

We look forward to seeing the revised manuscript and thank you for the opportunity to review your work.

[REDACTED]

Decision Letter, first revision:

9th April 2021

*Please ensure you delete the link to your author homepage in this e-mail if you wish to forward it to your co-authors.

Dear Dr Bates,

Your manuscript entitled "Spatial patterns of tumour growth impact clonal diversification: computational modelling and evidence in the TRACERx Renal study" has now been seen by 3 reviewers, whose comments are attached. The reviewers have raised a number of concerns which will need to be addressed before we can offer publication in Nature Ecology & Evolution. We will therefore need to see your responses to the criticisms raised and to some editorial concerns, along with a revised manuscript, before we can reach a final decision regarding publication.

We are committed to providing a fair and constructive peer-review process. Do not hesitate to contact us if there are specific requests from the reviewers that you believe are technically impossible or unlikely to yield a meaningful outcome.

natureresearch

When revising your manuscript:

* Include a “Response to reviewers” document detailing, point-by-point, how you addressed each reviewer comment. If no action was taken to address a point, you must provide a compelling argument. This response will be sent back to the reviewers along with the revised manuscript.

* If you have not done so already please begin to revise your manuscript so that it conforms to our Article format instructions at http://www.nature.com/natecolevol/info/final-submission. Refer also to any guidelines provided in this letter.

* Include a revised version of any required reporting checklist. It will be available to referees (and, potentially, statisticians) to aid in their evaluation if the manuscript goes back for peer review. A revised checklist is essential for re-review of the paper.

Please use the link below to submit your revised manuscript and related files:

[REDACTED]

Note: This URL links to your confidential home page and associated information about manuscripts you may have submitted, or that you are reviewing for us. If you wish to forward this email to co-authors, please delete the link to your homepage.

We hope to receive your revised manuscript within four to eight weeks. If you cannot send it within this time, please let us know. We will be happy to consider your revision so long as nothing similar has been accepted for publication at Nature Ecology & Evolution or published elsewhere.

Nature Ecology & Evolution is committed to improving transparency in authorship. As part of our efforts in this direction, we are now requesting that all authors identified as ‘corresponding author’ on published papers create and link their Open Researcher and Contributor Identifier (ORCID) with their account on the Manuscript Tracking System (MTS), prior to acceptance. ORCID helps the scientific community achieve unambiguous attribution of all scholarly contributions. You can create and link your ORCID from the home page of the MTS by clicking on ‘Modify my Springer Nature account’. For more information please visit please visit www.springernature.com/orcid.

Please do not hesitate to contact me if you have any questions or would like to discuss these revisions further.

We look forward to seeing the revised manuscript and thank you for the opportunity to review your work.

[REDACTED]

Reviewers' comments:

Reviewer #1 (Remarks to the Author):

The authors investigated spatial features of clonal diversification through a combined computational modelling and experimental analysis in the TRACERx (Tracking cancer evolution through therapy) renal study. Six hundred and six regions from 54 tumors were analyzed.

1. Repositories that link patients' clinical and genomic data could be extremely valuable for understanding the tumor diversity. An algorithm should be proposed that uses the measurement of intra-tumor genetic heterogeneity to associate with clinical phenotype to form a clinical annotation.
2. Patient clinical outcome data should be presented and align with the diversity status.
3. The authors found that metastasis-competent subclones were enriched at the tumor center, suggesting that environmental factors favored their selection. Supportive data from metastatic tumors should be presented and discussed.
4. The authors should explain if the initial tumor mutations contribute to subsequent mutations in subclones, such as VHL inactivated and non-inactivated tumors.
5. Beside the environment insults, the authors should also address if other mutation mechanisms are involved. A "passenger" mutation can become a "driver" in the context of tumor progression or therapy resistance.
6. The genetic diversity among tumors and within tumor in multifocal tumors should be evaluated. Measurement algorithm should be proposed.
7. All of the abbreviations should have a full form at the first time. All the references should follow the journal style.

Reviewer #2 (Remarks to the Author):

This study uses computational models to examine patterns of clonal diversity in solid tumours. Model outcomes are compared to data from clear-cell renal cell carcinomas (ccCRC) from the TRACERx Renal cohort. The article is exceptionally well written and clearly structured. As far as I can tell without access to the code, the methods appear sound.

Although some results more or less confirm what has been reported in previous studies (such as those cited in the Discussion), other findings are both interesting and original. A particular strength is that the models incorporate specific features of ccCRC, so that the simulation results are readily comparable to data for that tumour type, with which the authors are uniquely familiar.

The examination of budding structure (pages 10-11; Figure 6) and the "replay" analysis (page 11; Supplemental Figure 8) are especially novel and interesting. Weaker points include apparent flaws in the analysis of the spatial distribution of microdiversity hotspots, and lack of explanation for differences between surface growth and volume growth models.

I have several specific comments on the reporting of the methods and results.

Major comments:

The analysis of the spatial distribution of microdiversity hotspots, which is reported as a main result (page 3 lines 9-10; page 9 lines 11-32; page 13 lines 4-6; page 17 lines 15-18; Figure 4e-g; Supplemental Figure 5; Supplemental Figure 9c-d) is unconvincing, simply because there are more

voxels near the surface of a tumour than near the core. Specifically, the number of voxels at distance d from the core increases with d squared, and it follows (from integration) that the cumulative distribution of microdiversity hotspots is expected to obey a power law with exponent 3 even if the hotspots are uniformly distributed throughout the tumour. It's therefore wholly unsurprising to observe a power law in both simulations and actual tumours, and it's unremarkable that the exponent is close to 3. If the authors agree with my reasoning then they should assess the spatial distribution relative to the expectation based on a uniform distribution (i.e. $k = 3$). Otherwise they should explain where I've gone wrong.

There's a missed opportunity to learn more about why outcomes were different for the surface growth model versus the volume growth model. Even if the authors can't demonstrate causation, it would be useful to at least examine correlations. In particular, the baseline growth curves (in the absence of mutation) are qualitatively different for the two models: one is polynomial and the other is exponential. My guess is that, even with mutation, the growth curve of the surface growth model is typically less convex, and it takes longer to reach the stopping condition of one million voxels. This would imply that the surface growth model has more birth events and hence a greater supply of mutations, especially while the tumour is relatively small, which might help explain why this model generates more clonal diversity. I suggest reporting the number of voxel divisions that occurred during tumour growth, and the timing of these divisions. Then the authors can comment on whether differences in growth curves might or might not help to explain differences in how the simulated tumours evolved. Note that here I'm using "mutation" in a broad sense, to include driver SCNAs.

Page 12, lines 11-12: "An important finding, via computational modelling, is that different spatial patterns of tumour growth impact the extent of subclonal diversification and shape divergent modes of evolution." Calling this a "finding" is misleading given that previous studies – such as those cited at the end of the paragraph – have reached the same conclusion. For example, the cited article by Noble et al. 2019 found "that differences in the range of cell-cell interaction and the mode of cell dispersal can explain the spectrum of evolutionary modes observed in human tumours". It would be fairer and more accurate to say that the current study corroborates or builds upon previous findings in this regard. Other precedents that could be cited here include Antal et al. 2015 (https://doi.org/10.1103/PhysRevE.92.022705), Ahmed & Gravel 2017 (https://doi.org/10.1093/molbev/msy115), Noble et al. 2020 (https://doi.org/10.1111/eva.13057), and West et al. 2021 (https://www.nature.com/articles/s41467-021-22123-1). The authors should rephrase to acknowledge this prior work.

Why did the authors choose to examine mutation rates between 2×10^-4 and 1×10^-3, relative to the voxel birth rate? Can they cite data to support this range of values?

It's unclear how the simulation worked in terms of choosing events (births, deaths and mutations). Is it some kind of Gillespie algorithm? This method should be explained more clearly.

It's also unclear exactly how the "evolutionary replays" were done. I guess that each simulation was initiated with a different seed for the pseudorandom number generator used in the Gillespie algorithm (or similar). This method should be made explicit.

What is the "Null model" curve in Figure 4e? This should be explained in the figure legend.

Typos:

“Supplemental Note 4. Evolutionary replay in silico” refers to Supplemental Figure 5, but I think it should be Supplemental Figure 8.

“Supplemental Note 5. Scaling between clonal diversity and sampling area” refers to Supplemental Figure 6, but I think it should be Supplemental Figure 9.

Reviewer #3 (Remarks to the Author):

This work by Fu et al uses in silico modeled tumor systems to evaluarate the evolutionary patters of RCC. This tumor type is ideally suited to this type of modeling to infer spatial patters of evolution. The authors apply two models-a volume proliferation and surface proliferation model and replicate some findings in multiply sampled tumor specimens. This study specifically explores microdiversity and parallel evolution--both topics of interest, and difficult to assess in tumor specimens.

A few questions to consider:

Fundamentally, determining the pattern of evolution of metastatic clones is not possible in the current model, could this be more clearly addressed, using information from TracerX sampled metastases?

The limitation to two base models seems overly simplistic given what we do know about tumor heterogeneity.

The focus on PBRM1 and BAP1 is not well rationalized, as these tumors were found to be the least heterogeneous. It would seem that the SETD2 mutation would be preferred to be modeled.

Overall, it is not clear if the modeling methodologies are inherently novel. The findings related to models of kidney cancer growth support considerations regarding surface outgrowth and create a conceptual framework, but the pattern of growth needs to be more directly related back to experimental data.

********************END********************

Author Rebuttal, first revision:

Response to reviewers' comments:

Reviewer #1 (Remarks to the Author):

The authors investigated spatial features of clonal diversification through a combined computational modelling and experimental analysis in the TRACERx (Tracking cancer evolution through therapy) renal study. Six hundred and six regions from 54 tumors were analyzed.

1. Repositories that link patients' clinical and genomic data could be extremely valuable for understanding the tumor diversity. An algorithm should be proposed that uses the measurement of intra-tumor genetic heterogeneity to associate with clinical phenotype to form a clinical annotation.

Response:

We thank the reviewer for raising the important point on linking patient's clinical and genomic data. For the data repository comment, we have included Supplemental Tables 1-4 in the revised manuscript and provide a detailed response below the Reviewer 1's Comment 2, which also relates to data reporting.

Concerning the important question raised as to how we can link intra-tumour heterogeneity (ITH) to clinical phenotype, we previously reported an association between overall genetic diversity (ITH and weighted genomic instability index (wgII) scores) and patient clinical outcome (Turajlic, Xu, Litchfield, *et al. Cell* (2018)).

[redacted]

(Figure 7c from reference: Turajlic, Xu, Litchfield, *et al. Cell* (2018))

In the present study, we focus on studying how tumour growth modes impact the emergence and spatio-temporal evolution of ITH, which brings us closer to understanding the impact of spatial features of ITH on clinical behaviour. In the revised manuscript, we demonstrated the association between spatial features of clonal diversity and clinical characteristics. In brief, we found that

- Both indolent mono-driver tumours and aggressive poly-driver tumours, showing a more uniform distribution of microdiversity hotspots, are mapped to Volume Growth models.
- Tumours with attenuated progression, showing more enrichment of microdiversity hotspots at the margin, are mapped to Surface Growth models.

We detail our analyses and findings below. In Figure 3, we showed that 606 regions within 54 tumours with at least 2 clones, termed microdiversity hotspots, were increasingly frequent towards the tumour margin; consistent with observations in the model, this increasing frequency is characterised by a power law distribution. To explore whether this pattern was associated with clinical characteristics, we further evaluated this pattern in subsets of tumours with different clinical behaviours, as annotated in our previous work (Turajlic, Xu, Litchfield, *et al. Cell* (2018)). When the

54 tumours were split into two subsets according to whether the patient has relapsed (270 tumour regions) or not (336 tumour regions), the subset where the patient has relapsed shows a significantly steeper gradient of spatially distributed microdiversity hotspots (i.e., larger power law exponent) than the subset where the patient hasn't (**Figure 3g, Supplementary Figure 13**). Furthermore, when the 54 tumours were split into three subsets according to the patterns of metastatic progression – attenuated progression (265 tumour regions), rapid progression (65 tumour regions), and no progression (276 tumour regions), the subset with attenuated progression showed the steepest gradient of spatially distributed microdiversity hotspots (**Figure 3h, Supplemental Figure 13**), in keeping with the finding of branched evolution and attenuated progression (Turajlic, Xu, Litchfield, *et al. Cell* (2018)). These analyses suggested that tumours mapped to a poorer clinical outcome were typically associated with a steeper spatial distribution of microdiversity hotspots and enrichment towards the tumour margin.

While an algorithm summarising this spatial feature of clonal diversity in single tumours is not feasible at the current stage, we propose that procedure to establish the algorithm will involve:

- More extensive sampling of tumour regional biopsies
- Analysis of clonal diversity in each tumour region
- Measurement of the distance from each region to the margin and to the centre
- Extraction of the power exponent from the cumulative probability distribution (see above)
- Survival analysis for subsets of tumours with large or small power law exponent

Changes in the revised manuscript:

1. Previous finding on association between overall genetic diversity and patient clinical outcome (Turajlic, Xu, Litchfield, *et al. Cell* (2018)) was cited and emphasised in the following text:
    - **a.** Introduction: **Line numbers 20-21 on Page 5**
    - **b.** Results: **Line numbers 1-2 on Page 14**
    - **c.** Tables: **Supplementary Table 1**
2. The new analysis showing that spatial features of microdiversity associates with patient clinical outcome was presented in the following text:
    - a. Results: **Line numbers 16-25 on Page 13**
    - b. Figures: **Figure 3g-h, Supplementary Figure 13**
    - c. Tables: **Supplementary Table 2**
    - d. More detailed discussion in **Supplementary Note 5**

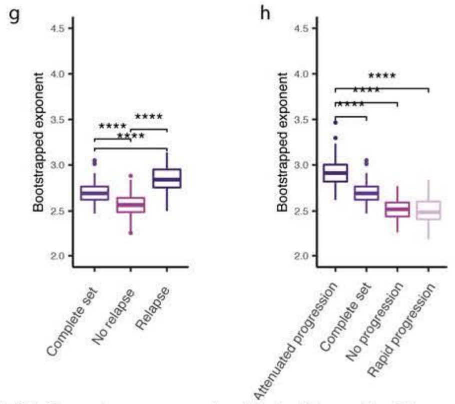

(This figure is presented as **Main Figure 3g-h**)

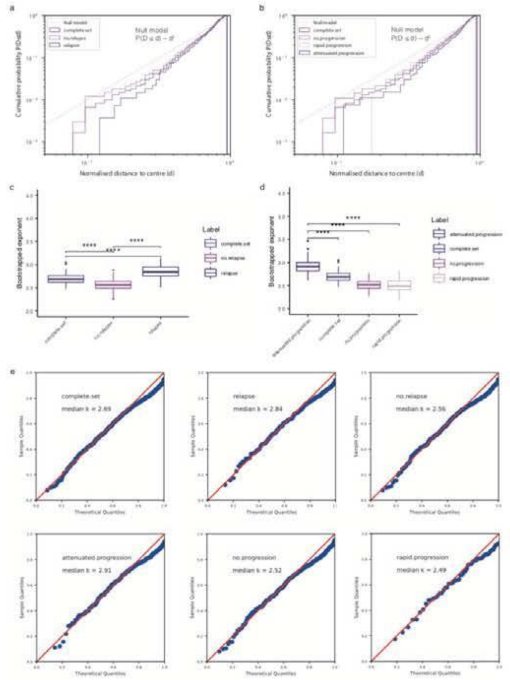

(This figure is presented as **Supplementary Figure 13**)

2. Patient clinical outcome data should be presented and align with the diversity status.

Response:

We thank the reviewer for highlighting this important point. The cohort in the current study represents a subset of 66 ccRCCs in the TRACERx100 Renal cohort published in Turajlic, Xu, Litchfield, *et al.* *Cell* (2018). In response to this comment and the data repository part of Comment 1 above, we have curated these data, including tumour details, evolutionary features, disease progression patterns, and clinical characteristics, for the subset of 66 tumours analysed in the current study, see Supplemental Table 1; the first few rows for which are presented below in this letter.

Table S1A: The subset of 66 tumours in the TRACERx Renal cohort: patient and tumour characteristics, reproduced from Turajlic, Xu, Litchfield, et al. Cell (2018)

Patient details			Tumour charcateristics													
Subject	Sex	Age	Histology	Overall Stage	T	N	M	Necrosis	Size of primary tumour (mm)	Leibovich score	Fuhrman Grade	Time to nephrectomy (days)	PFS (months)	Total follow up (months)	Outcome	Cancer related death (Y/N)
K021	M	71	Clear Cell	III	3a	X	0	Yes	32	6	3	39	14.5	43.7	Death	Y
K023	M	59	Clear Cell	III	3a	0	0	Yes	90	6	3	245	No progression event	49.2	Alive	-
K027	M	71	Clear Cell	I	1b	X	0	Yes	52	4	3	29	No progression event	44	Alive	-
K039	F	67	Clear Cell	IV	3a	1	1	Yes	125	9	3	133	3.5	42.7	Death	Y
K059	M	47	Clear Cell	III	3a	0	0	Yes	170	7	3	34	No progression event	46.4	Alive	-

(Complete data are presented in Table S1 - Subset of 66 tumours in TRACERx Renal - genomic and clinical characteristcs.xlsx)

Table S1B: - Evolutionary subtypes, progression patterns, clinical characteristics, reproduced from Turajlic, Xu, Litchfield, et al. Cell (2018)								
Patient	Overall Stage	TT (Y/N)	Leibovich score	Evolutionary subtype	ITH/WGII group	Progression group	wGII	ITH Index
K376	IV	N	11	Multiple Clonal Drivers	C3 Lo ITH Hi WGII	Rapid	0.46	0.00
K304	IV	N	9	Multiple Clonal Drivers	C3 Lo ITH Hi WGII	Rapid	0.42	0.33
K326	IV	Y	9	Multiple Clonal Drivers	C3 Lo ITH Hi WGII	Rapid	0.50	1.00
K263	IV	Y	8	Multiple Clonal Drivers	C3 Lo ITH Hi WGII	Rapid	0.64	0.56
K021	III	Y	6	Multiple Clonal Drivers	C3 Lo ITH Hi WGII	Attenuated	0.38	0.57

(Complete data are presented in Table S1 - Subset of 66 tumours in TRACERx Renal - genomic and clinical characteristcs.xlsx)

Additionally, we present new annotations, including spatial features of regional clone diversity (Supplementary Table 2; also see Figure 3) and parallel evolution (Supplementary Table 3; also see Figure 4) as well as the list of tumours with apparent budding structures (Supplementary Table 4; also see Figure 5). The first few rows from these tables are presented below in this letter.

Table S2 Spatial features of regional clone diversity.					
Patient	Region	Distance to margin (mm)	Distance to center (mm)	Number of clones	Microdiversity hotspot
K059	R10	24.77264364	25.02262908	1	No
	R11	14.30317551	42.03549363	1	No
	R13	7.669375559	76.87285624	2	Yes
	R2	7.641816901	44.85574283	1	No
	R3	9.81926839	31.0660747	1	No
	R4	4.47513904	39.25366092	2	Yes
	R5	10.50391872	71.9460464	1	No
	R6	8.621157279	42.07074601	1	No
	R8	7.70331501	70.53838963	1	No
	R9	7.930085722	52.82108111	2	Yes
K065	R1	7.434201859	13.9831164	1	No
	R2	6.748170049	11.19924491	1	No
	R3	9.655467899	12.88090057	1	No
	R4	5.44038835	13.76637873	1	No
	R5	4.333349665	24.06844241	1	No
	R6	3.6288442	17.65066948	1	No
	R7	7.803282976	15.48789324	1	No
	R8	4.932971222	25.06673572	1	No

(Complete data are presented in Table S2 - Spatial features of regional clone diversity.xlsx)

Table S3 Spatial features of parallel evolution in gene mutations.					
Patient	Genes	Parallel mutational event	Number of regions spanned by mutation	Total number of regions sampled	Max distance to margin (mm)
K096	MUC16	MUC16 19 9064616 G T T K096	12	12	22.10826899
K096	MUC16	MUC16 19 9086085 G A T K096	12	12	22.10826899
K099	PIEZO2	PIEZO2 18 10682214 G C R K099	3	3	11.61835018
K099	PIEZO2	PIEZO2 18 10787180 C T R K099	3	3	11.61835018
K252	BAP1	BAP1 3 52438527 C A G K252	2	7	7.331545802
K252	BAP1	BAP1 3 52440348 52440350 GGC - G K252	1	7	6.84508998
K252	BAP1	BAP1 3 52440856 52440873 GAGGC - G K252	1	7	5.358169091

(Complete data are presented in Table S3 - Spatial features of parallel evolution in gene mutations.xlsx)

Table S4: Early-stage (≤7cm) tumours: radiomic and genomic characteristics.						
Patient details		Radiomic characteristics	Genomic characteristics			
Subject	Size of primary tumour (mm)	Presence of budding structure	Number of regions (sampled and sequenced)	Number of clones	PBRM1 present in any region?	BAP1 present in any region?
K097 L	43	Yes	5	4	Yes (Subclonal)	Yes (Subclonal)
K105	65	Yes	3	1	Yes (Clonal)	No
K124	70	Yes	69	5	Yes (Clonal)	No
K138	45	Yes	20	2	No	No
K158	38	Yes	n/a	n/a	n/a	n/a

(Complete data are presented in Table S4 - Early-stage (sub 7 cm) tumours - radiomic and genomic characteristics.xlsx)

The alignment of clinical outcome and overall diversity was investigated in the previous work and presented in the Supplemental Table 1. In the current study, we further reported an association

between spatial features of microdiversity and clinical behaviours, as detailed in the response to Reviewer 1's Comment 1.

Changes in the revised manuscript:

1. Previous finding on association between overall genetic diversity and patient clinical outcome (Turajlic, Xu, Litchfield, *et al. Cell* (2018)) was cited and emphasised in the following text:
    a. Introduction: **Line numbers 20-21 on Page 5**
    b. Results: **Line numbers 1-2 on Page 14**
    c. Tables: **Supplementary Table 1**
2. The new analysis showing that spatial features of microdiversity associates with patient clinical outcome was presented in the following text:
    a. Results: **Line numbers 16-25 on Page 13**
    b. Figures: **Figure 3g-h, Supplementary Figure 13**
    c. Tables: **Supplementary Table 2**
    d. More detailed discussion in **Supplementary Note 5**

3. The authors found that metastasis-competent subclones were enriched at the tumor center, suggesting that environmental factors favored their selection. Supportive data from metastatic tumors should be presented and discussed.

Response:

We thank the reviewer for raising this interesting question. Our recent analyses (Zhao *et al Nat. Ecol. Evol.* (2021)) provided evidence and detailed discussion on the spatial patterns of metastasis-competent subclones through combined histological and genomic evaluation. Briefly, in that work, we investigated the spatial distribution of tumour regions that contain genomic alterations associated with metastatic competence and found a predominant distribution of these alternations in the tumour centre. We further demonstrated via computational modelling that central necrosis could be a plausible mechanism for the enrichment of these genomic alterations at the tumour interior. Supportive data from metastatic tumours were presented and discussed in that work (Zhao *et al Nat. Ecol. Evol.* (2021)). In the current study, we referred to the previous study for related discussion.

[redacted]

(Figure 2d from Zhao et al. Nature Ecology & Evolution (2021))

[redacted]

(Figure 2f from Zhao et al. Nature Ecology & Evolution (2021))

To extend our modelling-based understanding of the impact of necrosis on selection of fit subclones in the tumour interior (Zhao *et al Nat. Ecol. Evol.* (2021)) and to explore the impact of necrosis on spatial and temporal features of clonal diversification, a focus in the current manuscript, we incorporated necrosis in a subset of our extended set of growth models (**Revision Table 1**). By defining and measuring fitness of individual tumour voxels, a metric that quantifies the growth probability given the list of drivers it harbours (See Methods), we found that necrosis led to enhanced fitness in the tumour interior, in keeping with our previous analysis (Zhao *et al Nat. Ecol. Evol.* (2021)) (**Figure 2f-g, Supplementary Figures 7-9**). In addition, we found that necrosis could result in a dramatic reduction in macrodiversity at later stage in large tumours under Surface Growth (**Figure 5c, Supplementary Figure 16**) but facilitate the enrichment of microdiversity hotspots and youngest subclones in the interior (**Supplementary Figure 12**).

Together, investigation on the impact of necrosis with additional analyses performed in our revised manuscript not only connected the current study to our previous work concerning the selection of fitter clones by harsher environments, but also broadened our view on how different growth models could influence spatial and temporal features of clonal diversification, the focus of the current study.

Changes in the revised manuscript:

1. Previous finding on metastasis-competent subclones at the tumour interior (Zhao et al. Nature Ecology & Evolution (2021)) was referred to and discussed in the following text:
    a. Introduction: **Line numbers 6-9 on Page 7**
    b. Results: **Line numbers 23-24 on Page 11**
    c. Discussion: **Line numbers 15-17 on Page 20**
2. New model analyses showing that necrosis enhances fitness at the tumour centre were presented in the following text:
    a. Results: **Line numbers 9-24 on Page 11**
    b. Figures: **Figure 2f-g, Supplementary Figures 6-9**
    c. More detailed discussion in **Supplementary Note 2**
3. New model analyses showing that necrosis impacts spatial and temporal features of clonal diversification were presented in the following text:
    a. Results: **Line numbers 12-14 on Page 13, Line numbers 25 on Page 14, Line numbers 1-5 on Page 15, Line numbers 11-19 on Page 16**
    b. Figures: **Figure 4d, Figure 5c, Supplementary Figure 12, Supplementary Figure 14, Supplementary Figure 16**
    c. More detailed discussion in **Supplementary Note 4**

Driver advantage model	Growth mode	Necrosis	Driver acquisition rate
Saturated (See Supp Figure 1)	Volume Growth	Absent	Low
			Moderate
			High
		Present	Low
			Moderate
			High
	Surface Growth	Absent	Low
			Moderate
			High
		Present	Low
			Moderate
			High
Additive (See Supp Figure 2)	Volume Growth	Absent	Low
			Moderate
			High
		Present	Low
			Moderate
			High
	Surface Growth	Absent	Low
			Moderate
			High

		Present	Low
			Moderate
			High

(**Revision Table 1 (presented for reviewers, only in this letter):** the extended set of model conditions)

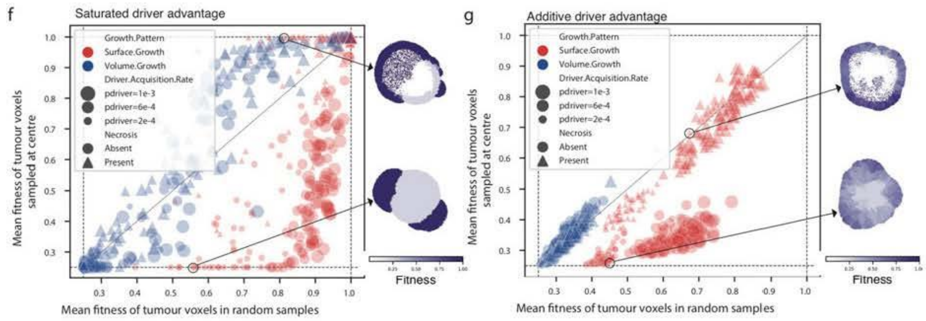

(This figure is presented as **Main Figure 2f-g**)

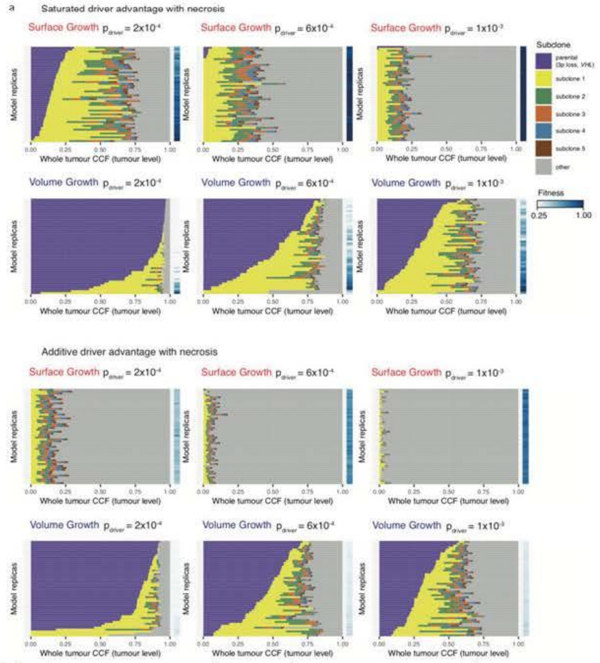

(This figure is presented as **Supplementary Figure 6**)

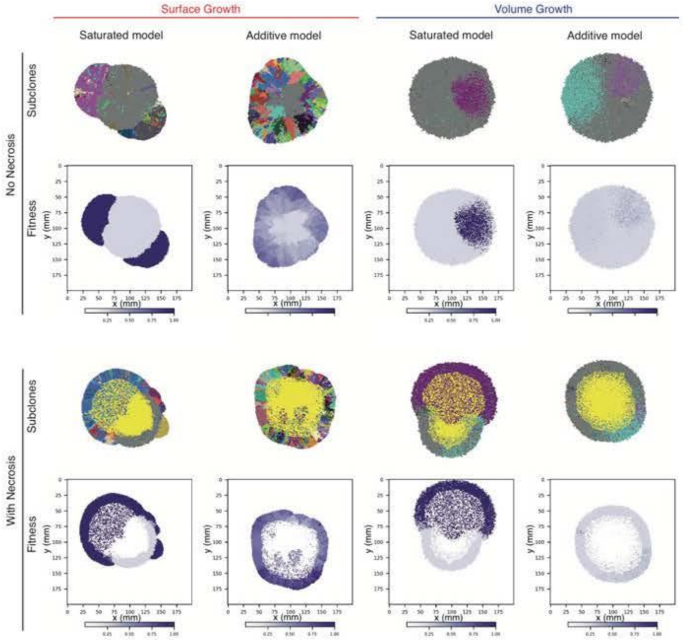

(This figure is presented as **Supplementary Figure 7**)

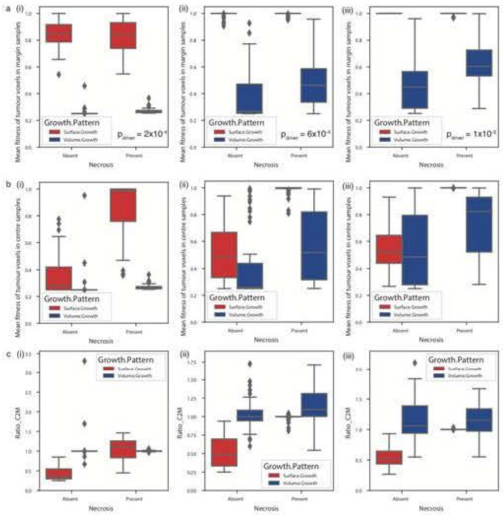

(This figure is presented as **Supplementary Figure 8**)

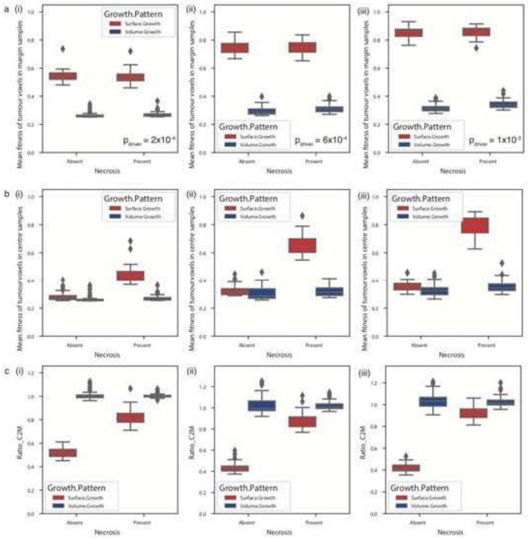

(This figure is presented as **Supplementary Figure 9**)

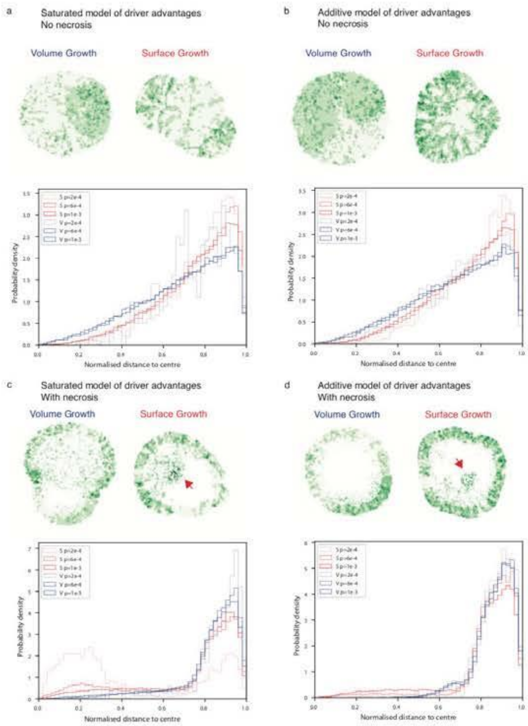

(This figure is presented as **Supplementary Figure 12**)

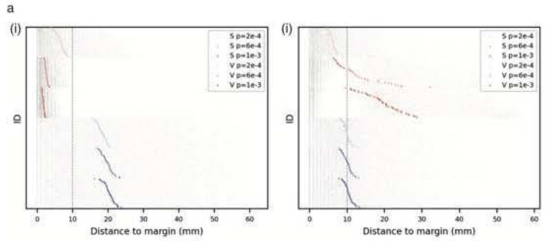

(This figure is presented as part of **Supplementary Figure 14**)

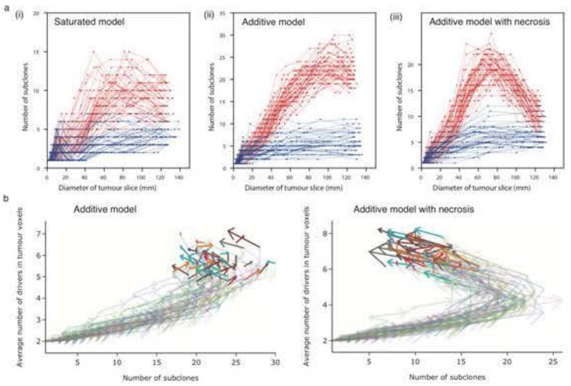

(This figure is presented as part of **Supplementary Figure 16**)

4. The authors should explain if the initial tumor mutations contribute to subsequent mutations in subclones, such as VHL inactivated and non-inactivated tumors.

Response:
In the present modelling work, we considered only *VHL* inactivated tumours, as the majority of ccRCCs have clonal *VHL* inactivation events, in general and in the Tx Renal cohort (Turajlic, Xu, Litchfield, et al. 2018. Cell). In the model, the founder tumour voxel of all simulated tumours harbour *VHL* inactivation and loss of 3p as clonal driver events. We assumed that mutation in *PBRM1* or *BAP1* promotes the acquisition of SCNAs, based on their association with chromosomal instability published in TRACERx Renal cohort (Turajlic, Xu, Litchfield, et al. 2018. Cell) as well as reported in pre-clinical studies (Varela, et al. 2012. Nature; Peng, et al. 2015. Cancer Lett.).

Changes in the revised manuscript:

1. The rationale for the assumption of *VHL* inactivation being one truncal event was stated in the following text:
    a. Results: **Line numbers 10-13 on Page 9**
2. The rationale for the assumption that *PBRM1 or BAP1* mutations promote acquisitions of SCNA was stated in the following text:
    a. Results: **Line numbers 13-15 on Page 9**

5. Beside the environment insults, the authors should also address if other mutation mechanisms are involved. A "passenger" mutation can become a "driver" in the context of tumor progression or therapy resistance.

Response:
We'd like to thank the reviewer for raising this interesting point. ccRCCs have a narrow repertoire of uniquely well-defined driver events, which reflect their strong selective advantage, as evidenced by their high frequency in the TRACERx cohort. Most clonal expansion associated with driver events in ccRCC is sustained throughout the life of a tumour. The "passenger" to "driver" phenomenon has not been reported in kidney cancer, potentially due to the absence of molecularly defined drug targets and mechanisms of drug resistance.
In response to the first comment, in the present work, we assumed that mutation and acquisition of SCNAs are proliferation dependent, which would imply DNA replication as the main source of genomic alterations. To clarify, the presence of necrosis, one of the potential environmental insults, is not assumed in the model to alter mutational mechanisms but is a source of selection for fitter clones. These simulation results are presented in response to the Comment 3 above.

Changes in the revised manuscript:
1. Clarification of model assumptions on these two points were provided in the following text:
    a. Results: **Line numbers 16-19 on Page 9**

6. The genetic diversity among tumors and within tumor in multifocal tumors should be evaluated. Measurement algorithm should be proposed.
Response:
In our computational modelling, we assessed the clonal diversity within individual simulated tumours and compare the results among distinct tumours (Figure 2C). Analyses on the genetic metrics, including ITH and wgII, in the TRACERx Renal cohort, have been previously published (Turajlic, Xu, Litchfield, et al. 2018. Cell). For reference, we have now curated data on the evolutionary features of the subset of 66 tumours analysed in the current study, see Supplemental Table 1. We have observed some patients with multiple tumours which we found to be clonally independent with distinct chromosome 3 and *VHL* events, or in one case a *VHL* and *TECB1* mutations, respectively (Turajlic, Xu, Litchfield, et al. 2018. Cell). In both cases, the pair of tumours only have *VHL* inactivation mono-driver yet distinct mutations, and therefore, lack macrodiversity (i.e., ITH = 0).

Changes in the revised manuscript:
1. Previous finding on overall genetic diversity and other annotations of the cohort (Turajlic, Xu, Litchfield, *et al. Cell* (2018)) was recapped in the following table:
    a. Tables: **Supplementary Table 1**

7. All of the abbreviations should have a full form at the first time. All the references should follow the journal style.

Response:
We would like to thank the reviewer for the important suggestion. In revision, we have added the full forms for the following abbreviations:
Introduction:
TRAcking Cancer Evolution through therapy (Rx) (TRACERx)
Methods:
Computed Tomography (CT)
picture archiving and communication system (PACS)
Extensible Neuroimaging Archive Toolkit (XNAT)
We have also changed the references to Nature style following journal guidelines.

Reviewer #2 (Remarks to the Author):

This study uses computational models to examine patterns of clonal diversity in solid tumours. Model outcomes are compared to data from clear-cell renal cell carcinomas (ccCRC) from the TRACERx Renal cohort. The article is exceptionally well written and clearly structured. As far as I can tell without access to the code, the methods appear sound.

Although some results more or less confirm what has been reported in previous studies (such as those cited in the Discussion), other findings are both interesting and original. A particular strength is that the models incorporate specific features of ccCRC, so that the simulation results are readily comparable to data for that tumour type, with which the authors are uniquely familiar.

The examination of budding structure (pages 10-11; Figure 6) and the "replay" analysis (page 11; Supplemental Figure 8) are especially novel and interesting. Weaker points include apparent flaws in the analysis of the spatial distribution of microdiversity hotspots, and lack of explanation for differences between surface growth and volume growth models.

Response:
We would like to thank Reviewer 2 for the complimentary comment on the presentation and structure of the manuscript and for the constructive suggestions on improving clarity of the microdiversity analysis and comparison between the two growth models. We are gratified that the Reviewer 2 found that our analyses on budding structure and evolutionary replay are especially novel and interesting.

As detailed in our point-by-point response, we have now included a Supplementary Table 4 related to budding structure for reference and a more detailed description of the "replay" analysis. Regarding microdiversity analysis, we have provided a more detailed explanation supplemented with additional analyses to support our existing conclusion. Regarding the comparison between two growth models, we have added various analyses, including some suggested by Reviewer 2, to provide a clearer picture illustrating how these growth modes lead to distinct clonal diversification.

I have several specific comments on the reporting of the methods and results.

Major comments:

The analysis of the spatial distribution of microdiversity hotspots, which is reported as a main result (page 3 lines 9-10; page 9 lines 11-32; page 13 lines 4-6; page 17 lines 15-18; Figure 4e-g; Supplemental Figure 5; Supplemental Figure 9c-d) is unconvincing, simply because there are more voxels near the surface of a tumour than near the core. Specifically, the number of voxels at distance d from the core increases with d squared, and it follows (from integration) that the cumulative distribution of microdiversity hotspots is expected to obey a power law with exponent 3 even if the hotspots are uniformly distributed throughout the tumour. It's therefore wholly unsurprising to observe a power law in both simulations and actual tumours, and it's unremarkable that the exponent is close to 3. If the authors agree with my reasoning then they should assess the spatial distribution relative to the expectation based on a uniform distribution (i.e. k = 3). Otherwise they should explain where I've gone wrong.

Response:

We would like to thank the reviewer for this very detailed comment. This comment motivates us to improve our presentation of "null model" conditions and to provide additional analyses for supporting the importance of spatial growth in shaping the emergent scaling patterns. In brief, we found that:

- The power law scaling exponent reflects the dimensionality of the analysis, as demonstrated by randomly sampling spots and measuring their normalised distances to centre
- Spatial homogenisation of subclone patterns in simulations led to the loss of characteristic power law scaling patterns in 2D tumour slices, with the exponent returning to approximately 2.

To begin with, we think that there could be a slight confusion around the dimensionality of the analysis performed. We appreciate that the reviewer's reasoning is indeed correct for the scaling of the cumulative distribution of microdiversity hotspots in three dimensions (3D). However, in Figure 4 (now Figure 3 in the revised manuscript), we focused our analyses entirely on two-dimensional (2D) tumour slices. The choice to focus on 2D provides convenience when comparing the same analysis applied to the tumour data. With the same intuition as the reviewer provided, the scaling of the cumulative distribution of microdiversity hotspots should have an exponent of 2, as the number of voxels at a distance of d from the centre increases with d in 2D in contrast to d squared in 3D. We performed this scaling analyses on random samples of spots in 1D, 2D, and 3D and found that the power law scaling exponent closely reflects the dimensionality of the analysis (**Supplementary Figure 11a-b**).
In addition to our analysis using random samples of spots, we further demonstrated the importance of spatial tumour growth and accordingly the spatial clonal structure in establishing the observed scaling pattern of microdiversity as follows. Briefly, we spatially homogenised the subclone patterns within the area of a tumour slice for each tumour (see Methods). Importantly, the proportions of subclones in the tumour slice were kept unchanged but the spatial organisation of subclones was entirely lost (**Supplementary Figure 11c-d**). Interestingly, this spatial homogenisation of subclones was sufficient to reduce the scaling exponent to approximately 2 (as would be expected in a random distribution), suggesting the importance of the emergent spatial organisation of subclones, as a consequence of spatial tumour growth, in shaping the scaling pattern of microdiversity hotspots (**Supplementary Figure 11e-f**).

Changes in the revised manuscript:

1. New analyses on spatial homogenisation of subclone patterns for demonstrating that characteristic scaling patterns arise from spatial tumour growth were presented in the following text:
    a. Results: **Line numbers 7-10 on Page 13**
    b. Figures: **Supplementary Figure 11**
    c. More detailed discussion in **Supplementary Note 3**

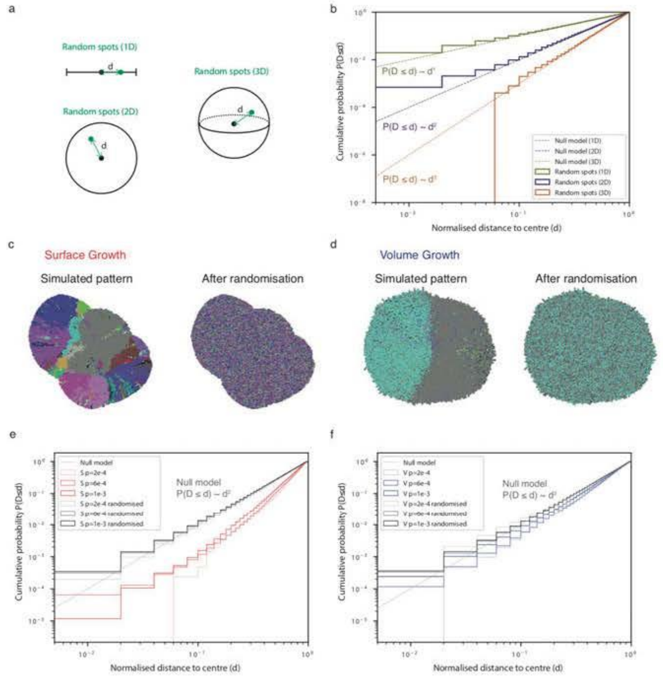

(The same figure is presented as **Supplementary Figure 11**)

There's a missed opportunity to learn more about why outcomes were different for the surface growth model versus the volume growth model. Even if the authors can't demonstrate causation, it would be useful to at least examine correlations. In particular, the baseline growth curves (in the absence of mutation) are qualitatively different for the two models: one is polynomial and the other is exponential. My guess is that, even with mutation, the growth curve of the surface growth model is typically less convex, and it takes longer to reach the stopping condition of one million voxels. This would imply that the surface growth model has more birth events and hence a greater supply of mutations, especially while the tumour is relatively small, which might help explain why this model generates more clonal diversity. I suggest reporting the number of voxel divisions that occurred during tumour growth, and the timing of these divisions. Then the authors can comment on whether differences in growth curves might or might not help to explain differences in how the simulated tumours evolved. Note that here I'm using "mutation" in a broad sense, to include driver SCNAs.

Response:
We would like to thank the reviewer for these very relevant suggestions. We have performed the suggested analyses including the time course of tumour growth and accumulation of drivers. Furthermore, we have expanded our investigation to the variation in tumour morphology and fitness over time. In brief, we found that:

- As Reviewer 2 correctly predicted, in our model, Surface Growth led to polynomial growth with longer time to reach the stopping condition, while Volume Growth resulted in exponential growth.
- Surface Growth models accumulated more drivers over time than Volume Growth models

- Surface Growth models favours a cycle of outgrowth of fitter subclones via surface budding followed by more accumulation of drivers in these fitter subclones

We detail these analyses below.

Surface Growth models showed slower tumour growth (**Revision Figures 1-5** in this letter).
By measuring the diameter of simulated tumour slices over time, we confirmed that the two base models differ in growth dynamics and the time it takes to grow to the final tumour size. This difference in growth dynamics was consistently observed in models with additive driver advantages and those with incorporation of necrosis. Reviewer 2 was indeed correct. Overall, Volume Growth models showed an exponential growth with the baseline growth probability, suggesting that the founder clone largely contributed to the overall growth and that subclonal diversification, and accordingly selection of advantageous subclones, was rare. In contrast, Surface Growth models showed regimes of polynomial growth, where growth rate was at the baseline early on and increased to higher levels at later stages. This increase in growth rate reflected continuing subclonal diversification and fixation of proliferatively advantageous subclones. Note that these growth dynamics are broadly concordant with mathematical formulation (Rodriguez-Brenes et al. 2013, Gerlee 2013) without considering the spatial context of tumour growth.
A closer comparison between alternative implementations of driver advantages in the Surface Growth models revealed that models with saturated driver advantages underwent sharper transition between regimes of polynomial growth, due to larger leaps in growth probability of a tumour voxel once acquiring strong drivers. By contrast, while in models with additive driver advantages the transition is more gradual and smoother as a tumour voxel becomes only slightly more advantageous when acquiring a new driver. This more gradual evolution enables the co-existence of subclones that had similar growth probabilities and generates an even higher clonal diversity in models with additive driver advantages.
Together, the faster growth rate in Volume Growth models means a large contribution of parental clone to overall tumour growth and shorter time for advantageous subclones to outgrow and compete, leading to tumours with limited diversification.

Surface Growth models showed more drivers accumulated over time (**Figure 5c, Supplementary Figure 16, Revision Figures 6-9** in this letter)
In addition to the inherent differences in growth dynamics between Surface and Volume Growth modes, the rate of accumulating advantageous drivers, which fuel faster subclone growth, should also contribute to the distinct features of clonal diversification.
To evaluate this, we assessed the average number of drivers accumulated by tumour voxels within a tumour slice over time. As a function of tumour size, Surface Growth models consistently show a greater number of drivers accumulated by tumour voxels on average than the Volume Growth models, regardless of model conditions (i.e., saturated or additive driver advantage; absence or presence of necrosis). While this finding confirmed Reviewer 2's correct reasoning about "a greater supply of mutations", we think that it's not as simple as the number of voxel divisions. In fact, to reach the same number of tumour voxels (i.e., same size of tumour), a tumour must undergo the same number of divisions, and in our analysis, we essentially normalised the number of drivers by the size of tumour. Instead of the number of tumour voxels being the reason, we think that Surface Growth models inherently favour a cycle of outgrowth of fitter subclones via budding at the tumour surface, followed by accumulation of more drivers in these fitter subclones, as they occupy the tumour frontier. In other words, for Surface Growth models, the number of drivers accumulated by tumour voxels was highly imbalanced since some tumour voxels became non-proliferative in the interior while the others remained proliferative at the frontier, thereby permitting accumulation of more drivers. Subsequently, a vicious cycle ensued, whereby these subclones had a greater chance of becoming more advantageous, by acquiring new drivers, and took up a higher proportion of the tumour, ultimately resulting in a larger number of drivers on average in the whole tumour.
With the implementation of necrosis, Surface Growth models showed accumulation of even more drivers on average, as necrosis eliminated the less advantageous subclones, those commonly with

fewer drivers. This permitted the outgrowth and continuing evolution of the more advantageous subclones in the tumour interior.
Collectively, a larger number of drivers were found accumulated in Surface Growth models, leading to a greater chance of the birth and outgrowth of advantageous subclones. Therefore, the observed differences in clonal diversification between growth models could be explained by and related to multiple orthogonal features, including growth dynamics, driver accumulation, tumour fitness and morphology (**Figure 5e**).

Changes in the revised manuscript:

1. New analyses on the time course of driver acquisition were presented in the following text:
    a. Results: **Line numbers 21-25 on Page 16, 1-3 on Page 17**
    b. Figures: **Figure 5c, Supplementary Figure 16**
2. New analyses for illustrating clonal diversification in conjunction with tumour fitness and morphology:
    a. Results: **Line numbers 15-18 on Page 17**
    b. Figures: **Figure 5e**

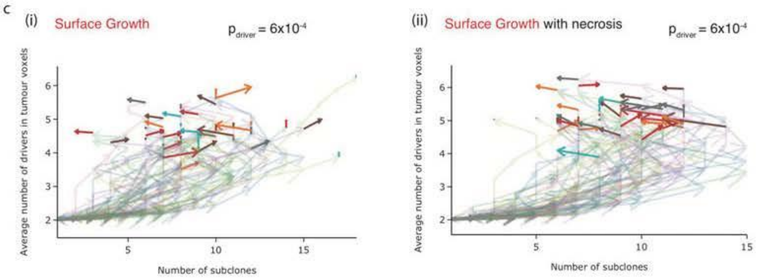

(This figure is presented as **Main Figure 5c**)

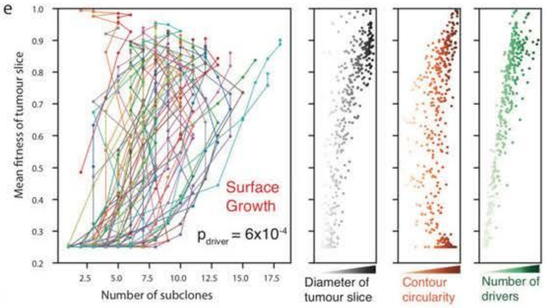

(This figure is presented as **Main Figure 5e**)

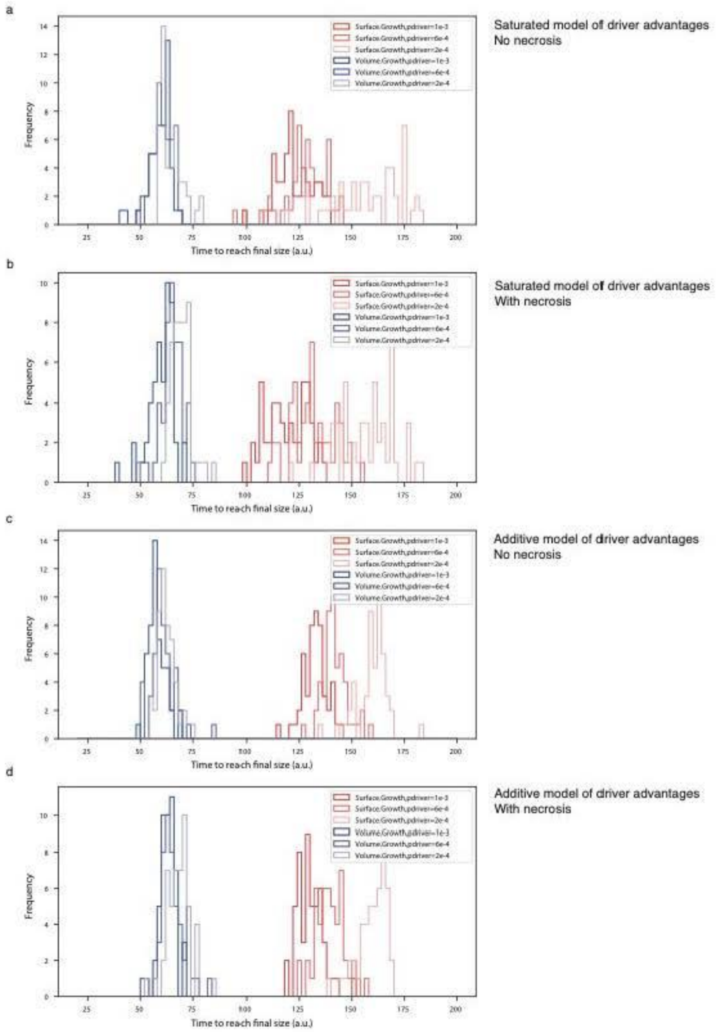

Revision Figure 1 (presented for reviewers, only in this letter). Time to reach final tumour size in various model conditions.

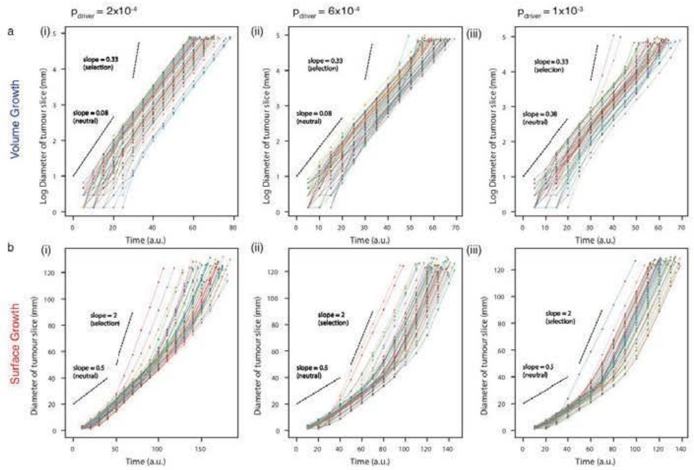

Revision Figure 2 (presented for reviewers, only in this letter). Growth dynamics of tumour slices, with implementation of “saturated” driver advantages, under Volume Growth (a) or Surface Growth (b). Panels (i)-(iii) reflect runs with varying driver acquisition probability. N = 50 runs for each condition.

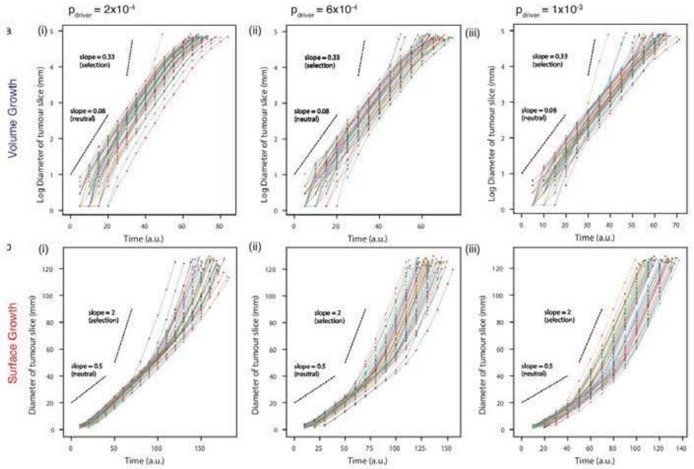

Revision Figure 3 (presented for reviewers, only in this letter). Growth dynamics of tumour slices, with implementation of “additive” driver advantages, under Volume Growth (a) or Surface Growth (b) with central necrosis. Panels (i)-(iii) reflect runs with varying driver acquisition probability. N = 50 runs for each condition

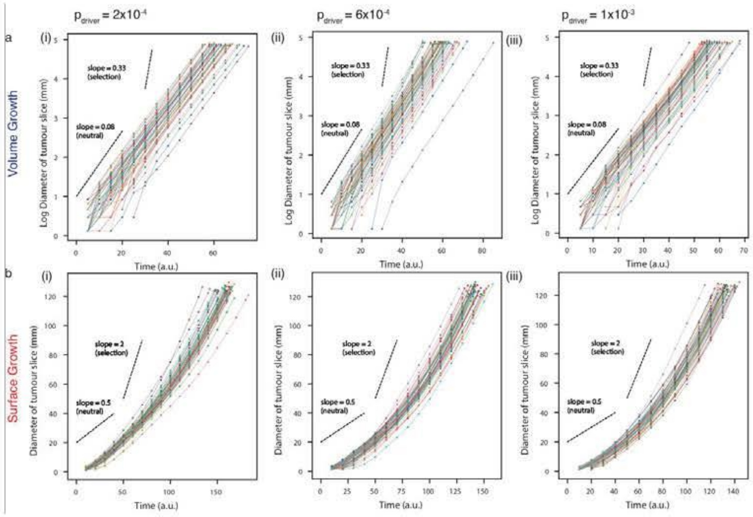

Revision Figure 4 (presented for reviewers, only in this letter). Growth dynamics of tumour slices, with implementation of "saturated" driver advantages, under Volume Growth (a) or Surface Growth (b). Panels (i)-(iii) reflect runs with varying driver acquisition probability. N = 50 runs for each condition.

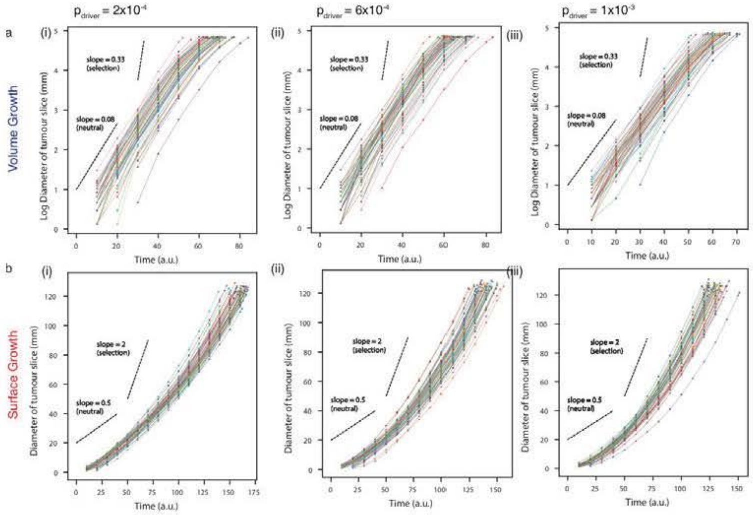

Revision Figure 5 (presented for reviewers, only in this letter). Growth dynamics of tumour slices, with implementation of "additive" driver advantages, under Volume Growth (a) or Surface Growth (b) with central necrosis. Panels (i)-(iii) reflect runs with varying driver acquisition probability. N = 50 runs for each condition

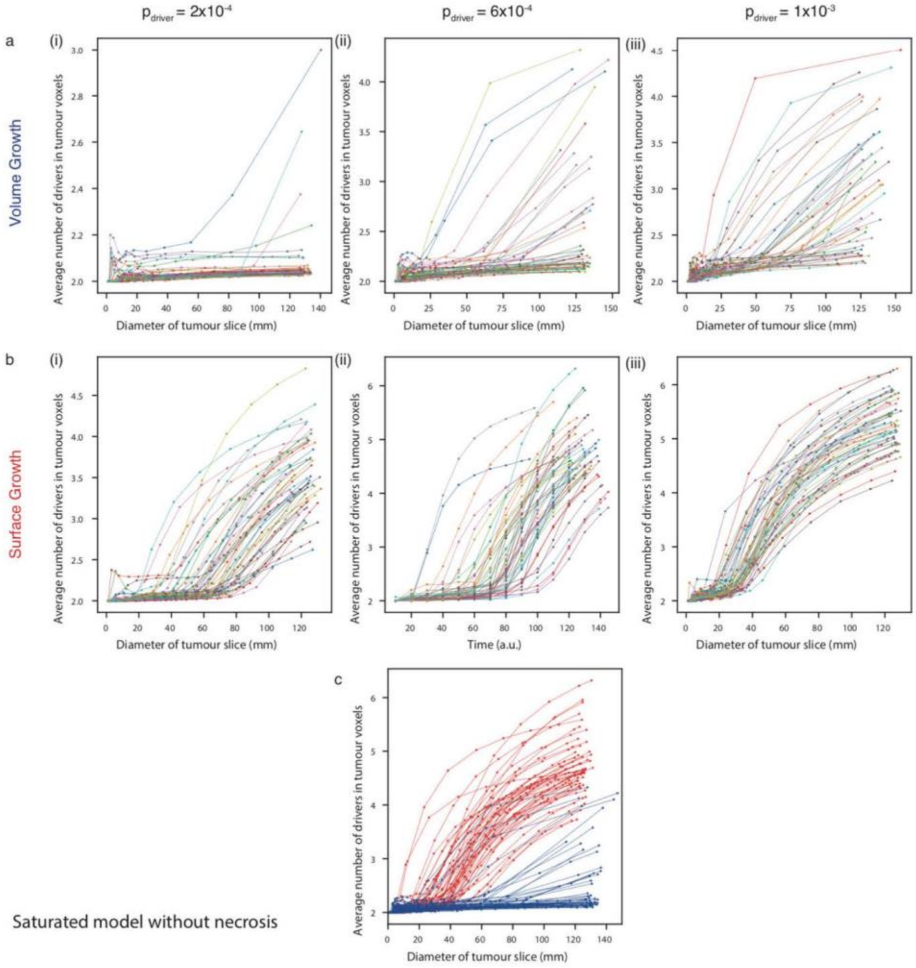

Revision Figure 6 (presented for reviewers, only in this letter). Average number of drivers harboured by tumour voxels over time, with implementation of “saturated” driver advantages, under Volume Growth (a) or Surface Growth (b). Panels (i)-(iii) reflect runs with varying driver acquisition probability. An overlay of observations from (a-ii) and (b-ii) is shown in (c). N = 50 runs for each condition.

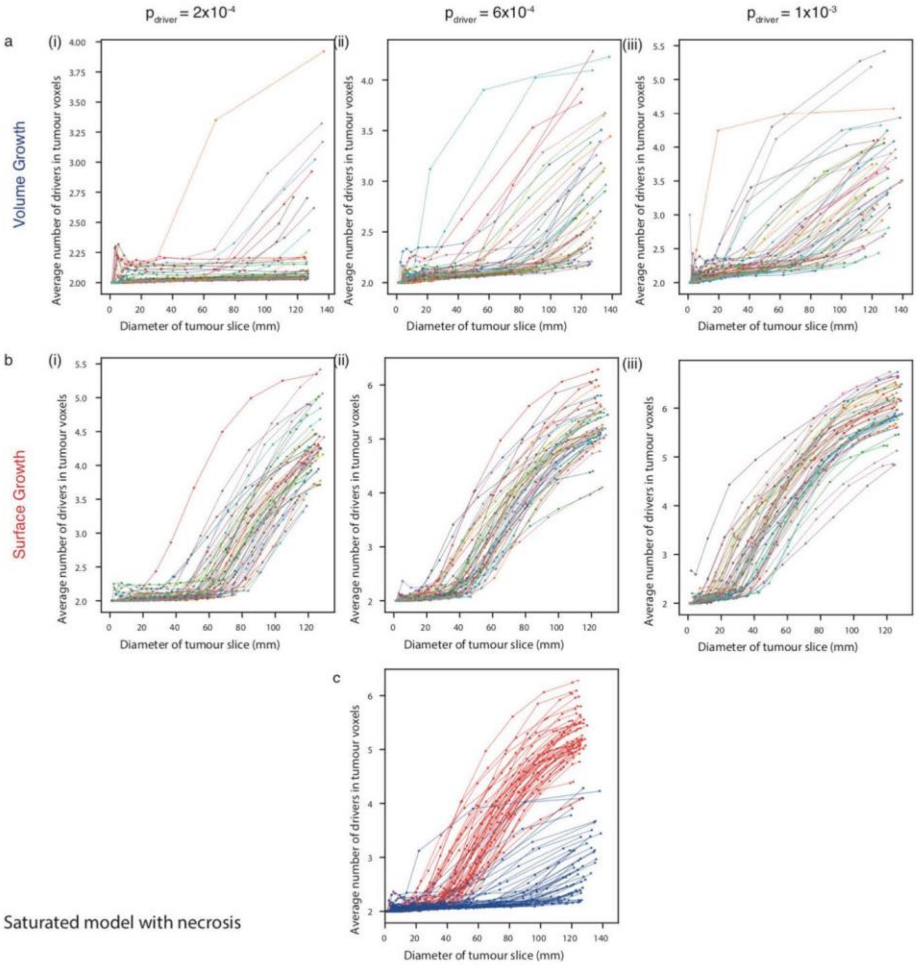

Revision Figure 7 (presented for reviewers, only in this letter). Average number of drivers harboured by tumour voxels over time, with implementation of "saturated" driver advantages, under Volume Growth (a) or Surface Growth (b) with central necrosis. Panels (i)-(iii) reflect runs with varying driver acquisition probability. An overlay of observations from (a-ii) and (b-ii) is shown in (c). N = 50 runs for each condition.

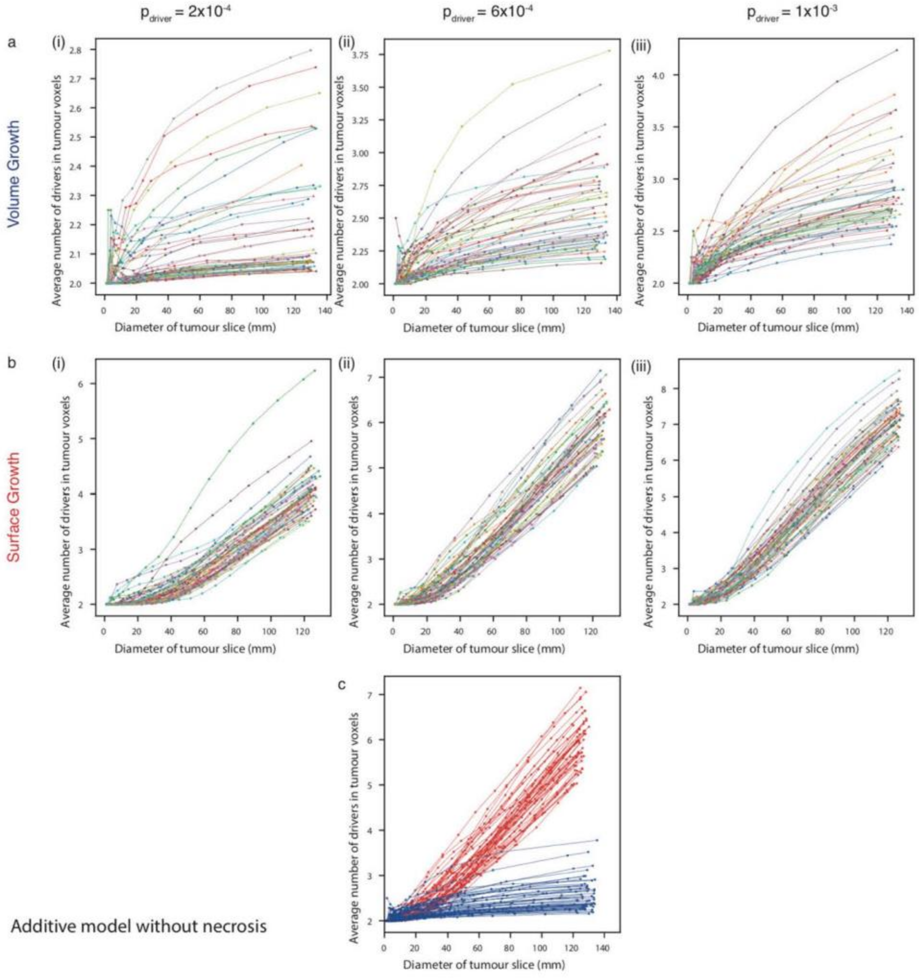

Revision Figure 8 (presented for reviewers, only in this letter). Average number of drivers harboured by tumour voxels over time, with implementation of "additive" driver advantages, under Volume Growth (a) or Surface Growth (b). Panels (i)-(iii) reflect runs with varying driver acquisition probability. An overlay of observations from (a-ii) and (b-ii) is shown in (c). N = 50 runs for each condition.

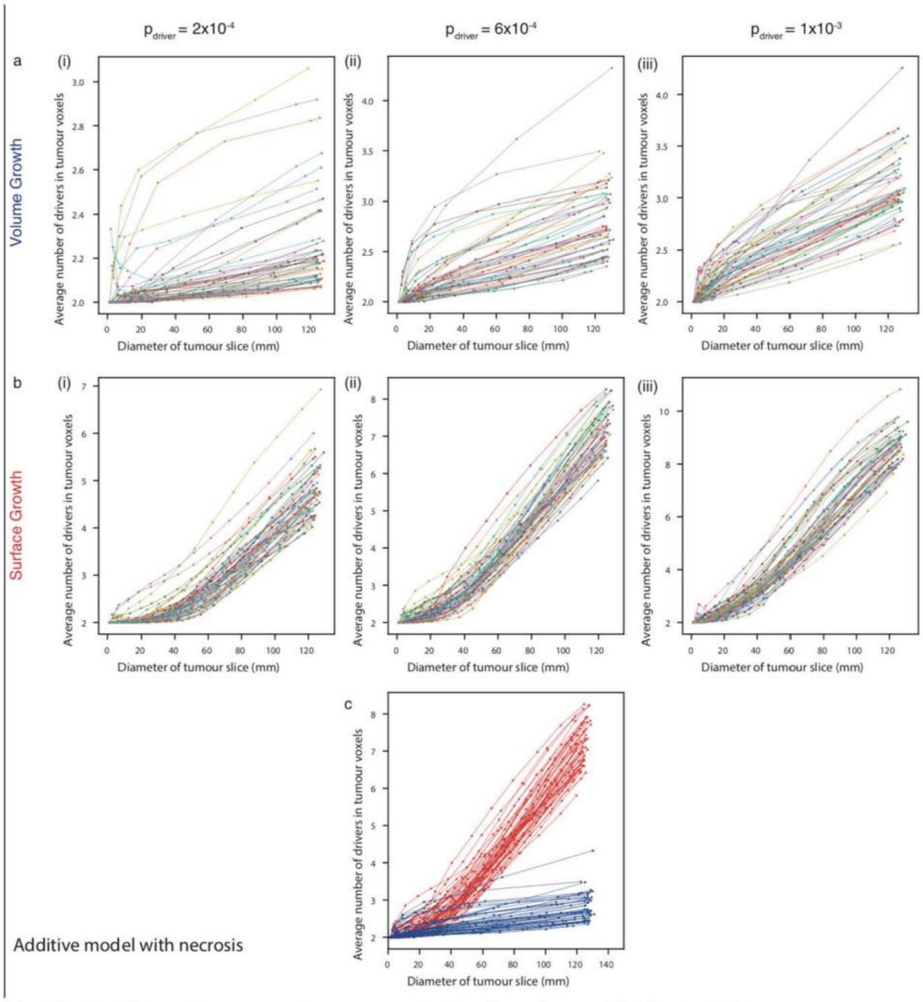

(A subset of these figure panels are presented in **Supplemental Figure 17**)

Revision Figure 9 (presented for reviewers, only in this letter). Average number of drivers harboured by tumour voxels over time, with implementation of "additive" driver advantages, under Volume Growth (a) or Surface Growth (b) with central necrosis. Panels (i)-(iii) reflect runs with varying driver acquisition probability. An overlay of observations from (a-ii) and (b-ii) is shown in (c). N = 50 runs for each condition.

Page 12, lines 11-12: "An important finding, via computational modelling, is that different spatial patterns of tumour growth impact the extent of subclonal diversification and shape divergent modes of evolution." Calling this a "finding" is misleading given that previous studies – such as those cited at the end of the paragraph – have reached the same conclusion. For example, the cited article by Noble et al. 2019 found "that differences in the range of cell-cell interaction and the mode of cell dispersal can explain the spectrum of evolutionary modes observed in human tumours". It would be fairer and more accurate to say that the current study corroborates or builds upon previous findings in this regard. Other precedents that could be cited here include Antal et al. 2015 (https://doi.org/10.1103/PhysRevE.92.022705), Ahmed & Gravel 2017 (https://doi.org/10.1093/molbev/msy115), Noble et al. 2020 (https://doi.org/10.1111/eva.13057), and West et al. 2021 (https://www.nature.com/articles/s41467-021-22123-1). The authors should rephrase to acknowledge this prior work.

Response:

We would like to thank the reviewer for the suggested changes and additional references. We have included these references and modified the relevant text in the Discussion.

"Adding to previous modelling work on spatial elements of tumour growth[26–29,33–37], our model demonstrated that growth modes impact subclonal diversification. Specifically, Volume Growth resulted in either limited evidence of evolution or punctuated evolution with early fixation of a fit clone, Surface Growth gave rise to branched evolution with extensive subclonal diversification (**Figure 6**)."

Changes in the revised manuscript:

1. New references were added to the re-phrased sentences in the following text:
   a. Discussion: **Line numbers 16-20 on Page 19**

Why did the authors choose to examine mutation rates between 2×10^-4 and 1×10^-3, relative to the voxel birth rate? Can they cite data to support this range of values?

Response:

We appreciate that this is a point slightly under-explained. For a number of reasons, we believe that choosing an exact driver acquisition rate in this coarse-grained model is fundamentally difficult and may be unnecessary for our aim of study:

Firstly, and most importantly, employing a mutation rate based on a "macroscopic" metric of evolutionary outcome via inference (e.g., as in Williams et al. (2016) Nature Genetics) may well lead to the risk of generating circular arguments in our study. As discussed in detail in response to one of Reviewer 2's main comments above, Volume Growth and Surface Growth models show very different time scales of growth and spatial extent and uniformity of mutation accumulation/supply. These two models would apparently differ in the inferred mutation rates given the same "macroscopic" metric. For example, to achieve the same Shannon diversity at the end, it requires a much higher driver acquisition rate for Volume Growth than for Surface Growth (Figure 2e). Thus, we chose to contrast two models always at the same driver acquisition rate, for a range of values, and with the same implementation of driver advantages.

Secondly, a mapping relationship from cell-level mutation rate per cell division to the effective mutation rate at the tumour voxel level is lacking and would be an interesting question for future study. With our coarse-grained approach, we focused on large-scale clonal dynamics and therefore neglected finer-scale clonal dynamics within each tumour voxel as well as the impact of cell migratory dynamics, which will be needed for establishing a mapping relationship. Interestingly, a

recent study started to shed lights on this question by simulating clonal dynamics under domains with varying sizes and found that the spatial constraint could influence the type of realised evolution. i.e., neutral vs. Darwinian evolution (West et al Nature Communications (2021)).

Thirdly, the ways to collecting samples could make inference of evolutionary parameters difficult. As nicely shown in Chkhaidze et al PLoS Comput Biol. (2019), spatial tumour growth and specific sampling procedures could influence the inferred type of evolution. Given that in the TRACERx cohort, the number and spatial distribution of regional samples vary among tumours, it's difficult to dissect the difference in mutation rates from these factors.

With all the considerations above, we decided to examine our model outputs across a range of driver acquisition rates and evaluate rates that range from very small value (**Supplementary Figure 4**) where both growth models lack macrodiversity to large value where both growth models show high macrodiversity. While this choice doesn't inform us of the mutation rates in kidney cancer, which is not the focus of our study, we were able to consistently contrast outcomes in clonal diversification between Volume Growth and Surface Growth models at the same driver acquisition rate, across a wide range of values.

It's unclear how the simulation worked in terms of choosing events (births, deaths and mutations). Is it some kind of Gillespie algorithm? This method should be explained more clearly.

Response:
We would like to thank the reviewer for raising this important point. To better explain our algorithm used to simulate tumour growth and evolution, we have now included a new paragraph in Methods supplemented by a detailed flow diagram illustrating the steps and sequence of calling modules in **Supplementary Figure 3**.

This paragraph was added to Methods:
Simulation
The procedure for simulating events of death, proliferation, and acquisition of driver events is illustrated in a flow diagram (**Supplemental Figure 3**). Briefly, each simulation starts from a single tumour voxel (i.e., founder tumour voxel) that harbours *VHL* mutation and 3p loss as truncal events, placed at the centre of the lattice, (x_0, y_0, z_0). During the evaluation of possible death events, for each of all tumour voxels alive, p_{death} is compared to a random number generated between [0,1]. If p_{death} is larger, a death event occurs, resulting in the lattice site freed for accommodating a newly born tumour voxel in the future. During the evaluation of possible proliferation events, for each of all valid tumour voxels (see above for the difference between Surface Growth and Volume Growth), p_{growth} is determined according to the driver events harboured (see above for the difference between "saturated" and "additive" models of fitness advantage) and compared to a random number generated between [0,1]. If p_{growth} is larger, a proliferation event occurs, resulting in a new tumour voxel created nearby. During the evaluation of possible acquisition of driver events, for each of all daughter tumour voxels just arising from proliferation and for each of the ccRCC drivers, p_{driver} is compared to a random number generated between [0,1]. If p_{driver} is larger and that driver is not currently harboured by the tumour voxel, acquisition of the driver takes place in the given tumour voxel. In a subset of simulations, necrosis is implemented. During the evaluation of necrotic death events, for each of all tumour voxels alive, if it's located at a distance of greater than $d_{necrosis}$ from the tumour surface, $p_{necrosis}$ is compared to a random number generated between [0,1]. If $p_{necrosis}$ is larger, a necrotic death event occurs, resulting in the lattice site freed for accommodating a newly born tumour voxel in the future. The simulation runs until the tumour grows to at least 1 million tumour voxels after the last simulation step. The computer code is written in CUDA C++.

Changes in the revised manuscript:

1. New paragraph with more detailed description of the computational algorithm was added in the following text:
    - **a.** Methods: **Line numbers 2-23 on Page 26, 1-2 on Page 27**
    - **b.** Figures: **Supplementary Figure 3**

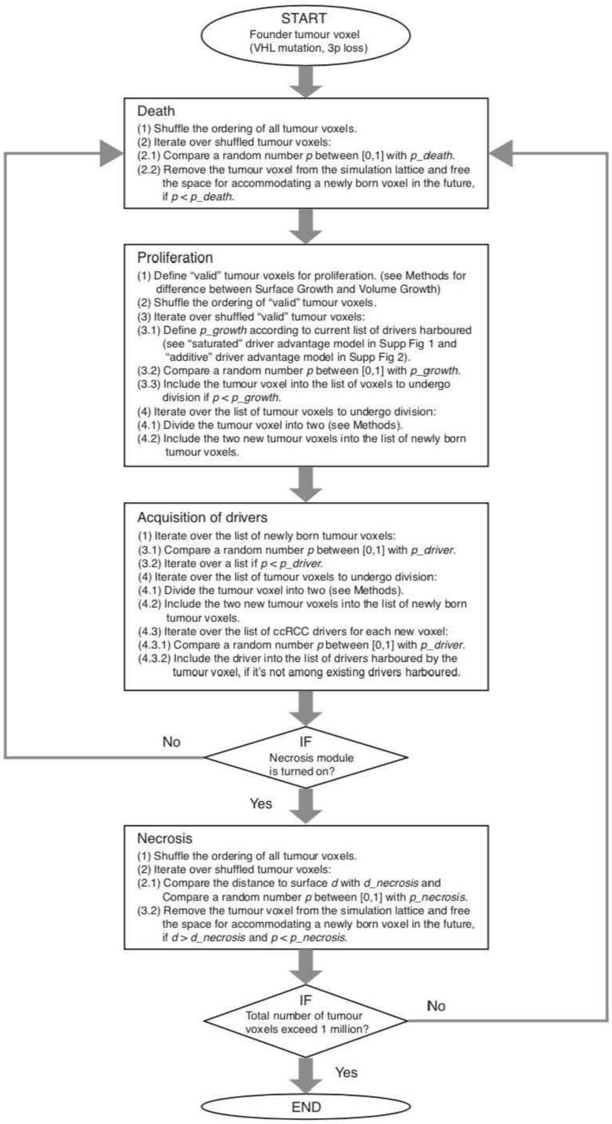

(This figure is presented as **Supplementary Figure 3**)

It's also unclear exactly how the "evolutionary replays" were done. I guess that each simulation was initiated with a different seed for the pseudorandom number generator used in the Gillespie algorithm (or similar). This method should be made explicit.

Response:
We would like to thank the reviewer for the suggestion on making this part more clearly described. To better explain the procedure of performing evolutionary replay, we have now included a paragraph in Methods, supplemented by a flow diagram illustrating the key steps in **Supplementary Figure 17**.

This paragraph was added to Methods:
Evolutionary replay
The procedure for simulating evolutionary replay is illustrated in a flow diagram (**Supplementary Figure 17**). Briefly, a preparation step is performed to create evolutionary snapshots of a simulated tumour at different time points. Specifically, each snapshot contains precise information about the positions, subclone identities, and drivers of all tumour voxels. At the beginning of evolutionary replay, *N* replicate tumours are reconstructed, each with a copy of the same evolutionary snapshot at a given time point *t*. Then, these replicate tumours undergo the events described above, each with a different unique random seed, and grow to the predefined stopping size. Evolutionary outcomes from these replicate tumours are evaluated and compared.

Changes in the revised manuscript:
1. New paragraph with more detailed description of the evolutionary replay procedure was added in the following text:
    a. Methods: **Line numbers 4-13 on Page 27**
    b. Figures: **Supplementary Figure 17**

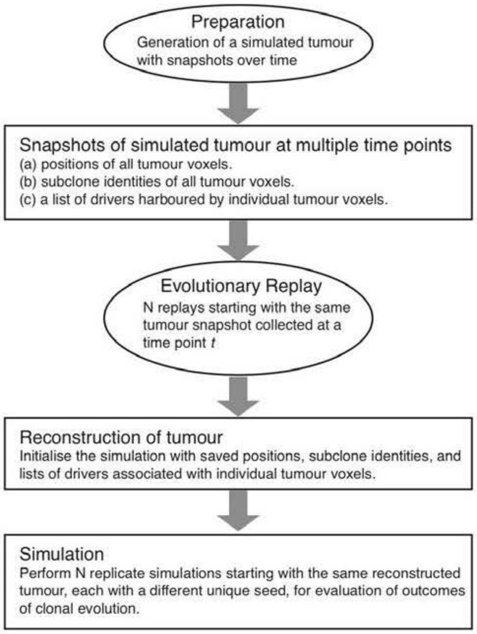

(This figure is presented as **Supplementary Figure 17**)

What is the "Null model" curve in Figure 4e? This should be explained in the figure legend.

Response:
As explained above in our response to Reviewer 2's main comment related to microdiversity patterns, the "Null model" curve in Figure 4e (now Figure 3e in revised manuscript) refers to the scaling results based on a power law with an exponent of 2.

Changes in the revised manuscript:
1. In-figure equation and figure legends were modified to explicitly clarify the "null model" in the following text:
    a. Figures: **Figure 3e, Supplementary Figure 11, Supplementary Figure 13**
    b. Figure legends: **Line numbers 16-17 on Page 44**

Typos:

"Supplemental Note 4. Evolutionary replay in silico" refers to Supplemental Figure 5, but I think it should be Supplemental Figure 8.

"Supplemental Note 5. Scaling between clonal diversity and sampling area" refers to Supplemental Figure 6, but I think it should be Supplemental Figure 9.

Response:
We would like to thank the reviewer for the suggested corrections for typos. We have carefully corrected the numbering for supplemental notes accordingly.

Reviewer #3 (Remarks to the Author):

This work by Fu et al uses in silico modeled tumor systems to evaluarate the evolutionary patters of RCC. This tumor type is ideally suited to this type of modeling to infer spatial patters of evolution. The authors apply two models-a volume proliferation and surface proliferation model and replicate some findings in multiply sampled tumor specimens. This study specifically explores microdiversity and parallel evolution--both topics of interest, and difficult to assess in tumor specimens.

A few questions to consider:

Fundamentally, determining the pattern of evolution of metastatic clones is not possible in the current model, could this be more clearly addressed, using information from TracerX sampled metastases?

Response:

We appreciate the reviewer's comment that the explanation on observation of metastasis-competent subclones in the tumour interior needs to be clearer. In our recent study (Zhao et al Nat. Ecol. Evol. (2021)), relevant data from metastatic tumours were presented in detail, through combined histological and genomic analyses regarding the spatial patterns of metastasis-competent subclones. In the separate analysis (Zhao et al Nat. Ecol. Evol. (2021)), we specifically looked at matched primary-metastasis pair from Tx Renal to determine the origin of metastasising clones at the primary tumour site. In brief, we found that metastasis-competent clones were significantly more likely to arise in the tumour interior, which is characterised by the presence of necrosis. In the complementary computational model in that study, we found that central necrosis could be a plausible mechanism for the enrichment of these genomic alterations at the tumour centre.

[redacted]

(Figure 2d from Zhao et al. Nature Ecology & Evolution (2021))

[redacted]

(Figure 2f from Zhao et al. Nature Ecology & Evolution (2021))

To extend our modelling-based understanding of the impact of necrosis on selection of fit subclones in the tumour interior (Zhao et al Nat. Ecol. Evol. (2021)) and to explore the impact of necrosis on spatial and temporal features of clonal diversification, a focus in the current manuscript, we incorporated necrosis in a subset of our extended set of growth models (**Revision Table 1**). By defining and measuring fitness of individual tumour voxels, a metric that quantifies the growth probability given the list of drivers it harbours (See Methods), we found that necrosis led to enhanced fitness in the tumour interior, in keeping with our previous analysis (Zhao et al Nat. Ecol. Evol. (2021)) (**Figure 2f-g, Supplementary Figure 7-9**). In addition, we found that necrosis could result in a dramatic reduction in macrodiversity at later stage in large tumours under Surface Growth but facilitate the enrichment of microdiversity hotspots and youngest subclones in the interior.

Together, investigation on the impact of necrosis with additional analyses performed in our revised manuscript not only connected the current study to our previous work concerning the selection of fitter clones by harsher environments, but also broadened our view on how different growth models could influence spatial and temporal features of clonal diversification, the focus of the current study. Additional consideration of fitness parameter for metastatic competence independent of proliferation-related metric and incorporation of microenvironment will be interesting in future studies.

Changes in the revised manuscript:

1. Previous finding on metastasis-competent subclones at the tumour interior (Zhao et al. Nature Ecology & Evolution (2021)) was referred to and discussed in the following text:
   a. Introduction: **Line numbers 6-9 on Page 7**
   b. Results: **Line numbers 23-24 on Page 11**
   c. Discussion: **Line numbers 15-17 on Page 20**
2. New model analyses showing that necrosis enhances fitness at the tumour centre were presented in the following text:
   a. Results: **Line numbers 9-24 on Page 11**
   b. Figures: **Figure 2f-g, Supplementary Figures 6-9**
   c. More detailed discussion in **Supplementary Note 2**
3. New model analyses showing that necrosis impacts spatial and temporal features of clonal diversification were presented in the following text:
   a. Results: **Line numbers 12-14 on Page 13, Line numbers 25 on Page 14, Line numbers 1-5 on Page 15, Line numbers 11-19 on Page 16**
   b. Figures: **Figure 4d, Figure 5c, Supplementary Figure 12, Supplementary Figure 14, Supplementary Figure 16**
   c. More detailed discussion in **Supplementary Note 4**

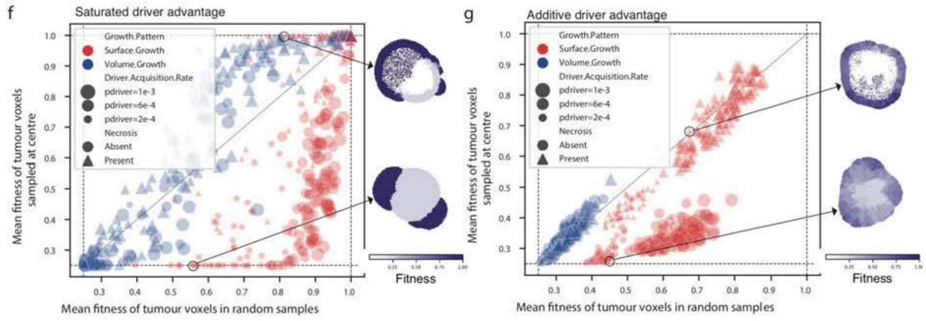

(This figure is presented as **Main Figure 2f-g**)

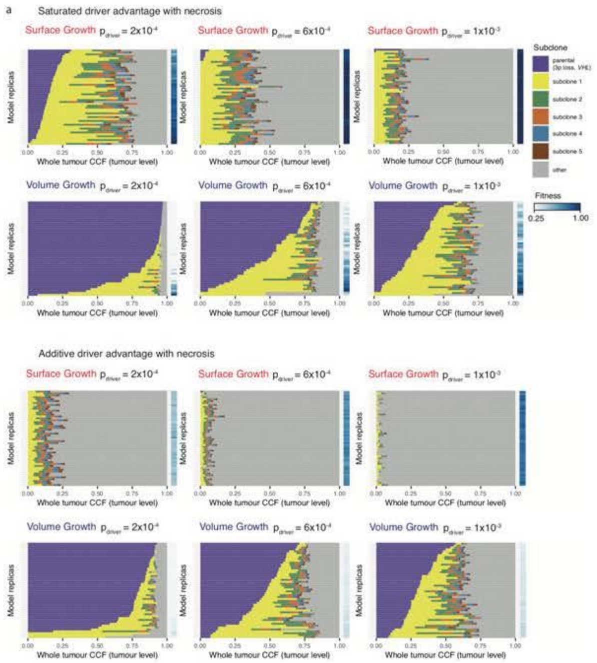

(This figure is presented as **Supplementary Figure 6**)

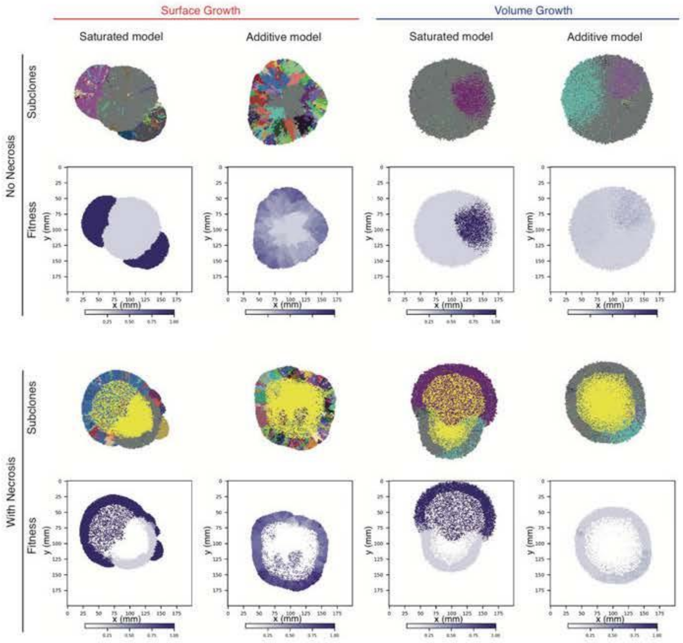

(This figure is presented as **Supplementary Figure 7**)

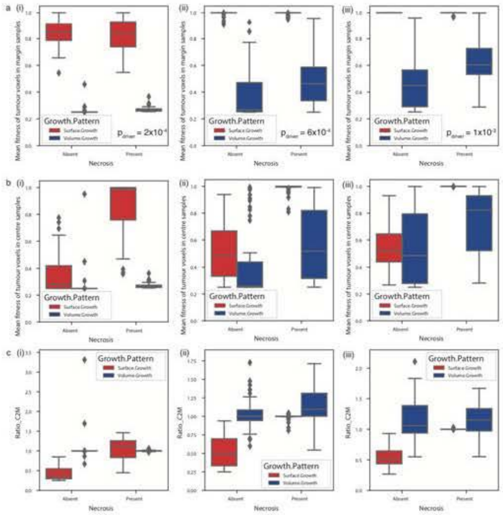

(This figure is presented as **Supplementary Figure 8**)

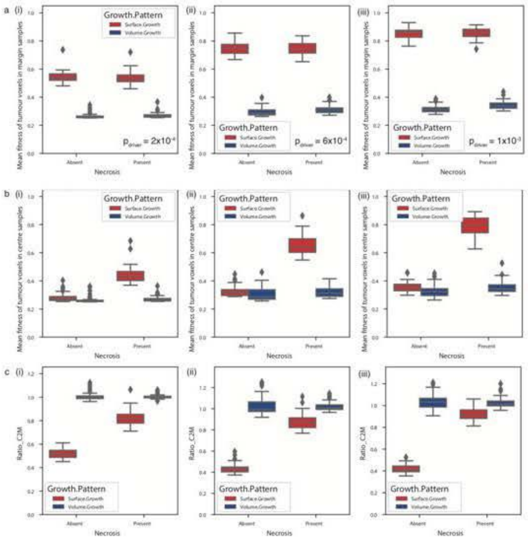

(This figure is presented as **Supplementary Figure 9**)

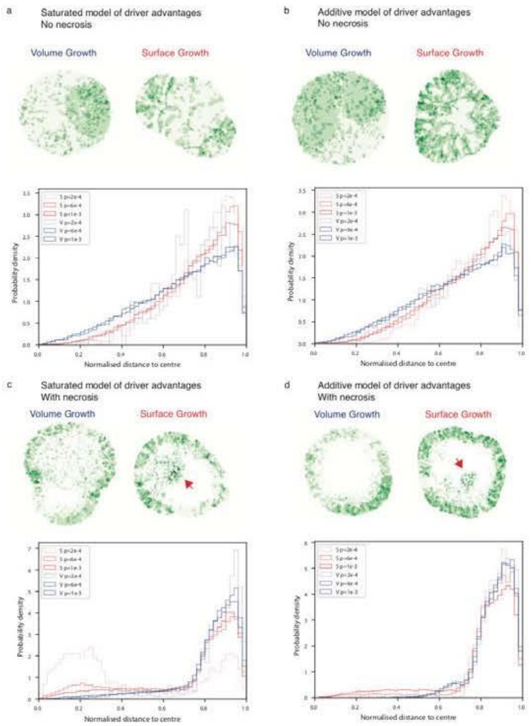

(This figure is presented as **Supplementary Figure 12**)

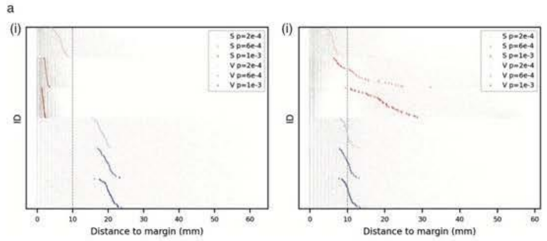

(This figure is presented as part of **Supplementary Figure 14**)

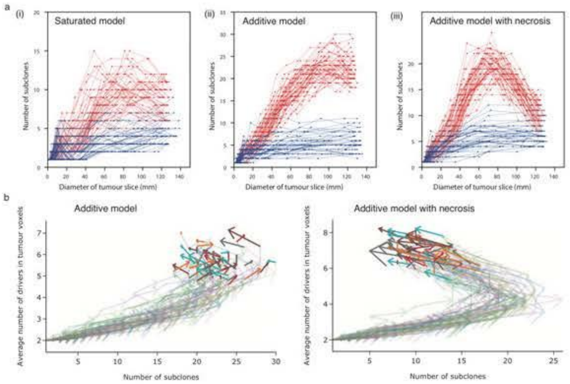

(This figure is presented as part of **Supplementary Figure 16**)

The limitation to two base models seems overly simplistic given what we do know about tumor heterogeneity.

Response:

We thank the reviewers for this very important point and acknowledge that solid tumours are characterised by varying extents and patterns of ITH and clonal complexities. To address the potential limitation of two base models in representing tumour growth and clonal evolution, we expanded the number of our base model conditions to 8, further considering two implementations of driver advantages (saturated vs additive) and two implementations of necrosis (absence vs presence) in the revised manuscript. In brief, the larger set of model conditions enabled to establish a richer understanding of spatial and temporal features of clonal diversification that align with observations in the tumour data:

- Volume growth models reflect both indolent mono-driver and aggressive poly-driver tumours while Surface growth models reflect tumours with attenuated progression, a finding enabled by corroborating microdiversity analysis in different growth models with that in groups of tumours showing distinct disease progression in the TRACERx Renal study
- Surface Growth models, especially in those with additive driver advantages or with necrosis incorporated, showed initial increase but subsequent collapse of clonal diversity, an observation that explains the static data of tumour size and clonal diversity in the TRACERx Renal study

Overall, these additional model conditions and observations provide sources for a more comprehensive comparison between modelling and tumour analysis in the future.

Changes in the revised manuscript:

1. Brief description of additional model conditions in the following text:
    a. Results: **Line numbers 1-6 on Page 9, Line numbers 9-14 on Page 11**
2. New model analyses showing that necrosis enhances fitness at the tumour centre were presented in the following text:
    a. Results: **Line numbers 9-24 on Page 11**
    b. Figures: **Figure 2f-g, Supplementary Figures 6-9**
    c. More detailed discussion in **Supplementary Note 2**
3. New model analyses illustrating the impact of diverse growth models on spatial features of clonal diversity
    a. Results: **Line numbers 10-15 on Page 13**

b. Figures: **Supplementary Figure 12**
c. More detailed discussion in **Supplementary Note 4**

4. New model analyses illustrating the impact of diverse growth models on spatial features of recent subclones
    a. Results: **Line numbers 18-25 on Page 14, 1-5 on Page 15**
    b. Figures: **Figure 4c-d, Supplementary Figure 14**
5. New model analyses illustrating the impact of diverse growth models on temporal features of clonal diversification
    a. Results: **Line numbers 11-19 on Page 16**
    b. Figures: **Figure 5c, Supplementary Figure 16**
6. New analyses on tumour data in terms of spatial features of clonal diversity connecting Volume Growth models to either indolent or aggressive tumours and Surface Growth models to tumours with attenuated progression
    a. Results: **Line numbers 16-25 on Page 13, 1-3 on Page 14**
    b. **Figures: Figure 3g-h, Supplementary Figure 13**
    c. More detailed discussion in **Supplementary Note 5**

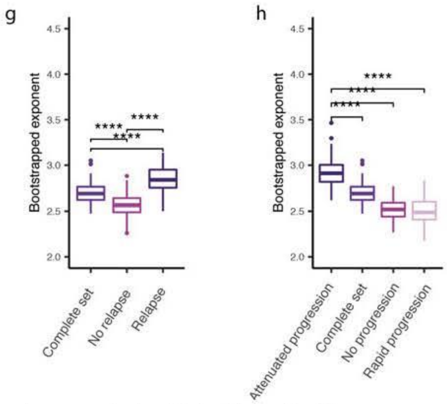

(This figure is presented as **Main Figure 3g-h**)

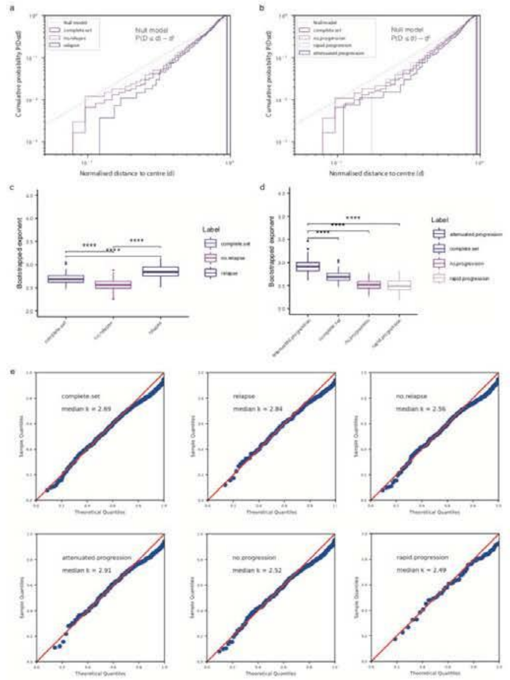

(This figure is presented as **Supplementary Figure 13**)

The focus on PBRM1 and BAP1 is not well rationalized, as these tumors were found to be the least heterogeneous. It would seem that the SETD2 mutation would be preferred to be modeled.

Response:

The justification for the choice of these two genes as our focus is their association with distinct evolutionary trajectory as well as their high frequency in ccRCCs. *PBRM1* mutation is linked to high levels of ITH and also clonal diversification, whereas is linked to low level of ITH and clonal sweep and early fixation of chromosomal instability. Acquisition of copy number alterations, due to chromosomal instability, is linked to metastatic potential (Turajlic, Xu, Litchfield, et al. 2018b. Cell). Recently, we showed that subclones with a higher burden of SCNAs were significantly more likely to be found at the tumour interior (Zhao et al. 2021). Both *BAP1* and *PBRM1* had the highest association with SCNAs in Tx Renal data (Turajlic, Xu, Litchfield, et al. 2018. Cell) and were mechanistically linked to chromosomal instability (Varela, et al. 2012. Nature; Peng, et al. 2015. Cancer Lett.). *SETD2* is usually after *PBRM1* along evolutionary trajectories, therefore its independent contribution is challenging to dissect. Moreover, *SETD2* events seem to offer a narrow fitness advantage and are frequently late in evolution, further complicating evaluation of their impact in the computational model.

Overall, it is not clear if the modeling methodologies are inherently novel. The findings related to models of kidney cancer growth support considerations regarding surface outgrowth and create a conceptual framework, but the pattern of growth needs to be more directly related back to experimental data.

Response:

We would like to thank the reviewer for these insightful comments. In the current study, through computational modelling, we aimed at addressing the unmet need for temporal understanding of clonal evolution and exploring predictive features of evolutionary trajectories. To this end, we focused on evaluating the contribution of tumour growth modes to clonal diversification in ccRCCs, with incorporation of ccRCC drivers characterised in the TRACERx Renal study (Turajlic et al. 2018). Our model revealed the potential of spatial and temporal features of clonal diversity as indication of future evolutionary trajectories. Several modelling analyses, including the patterns of microdiversity (association with patient clinical annotation included in the revision), budding structures (correlation with other features presented in the revision), and recent subclones (new analysis corroborated with genomic and histological evidence in the revision), were linked to the tumour data in the well annotated TRACERx Renal cohort. In brief, we found that

- Tumours with attenuated progression had a larger exponent, which is consistent with their more branched phylogenetic trees. Both indolent mono-driver and aggressive poly-driver tumours had lower exponents suggesting Volume Growth patterns, with the aggressive tumours simply determined in our model by the early acquisition of multiple strong drivers.
- Surface Growth models revealed a non-monotonic variation in clonal diversity over time with a dramatic collapse of diversity at large tumour sizes, consistent with the apparent relationship between static data of tumour size and clonal diversity in the TRACERx Renal study.
- Patterns of proliferation and necrosis in diverse growth models underlie the spatial features recent subclone births. Examination of this feature in the tumour data led us to inferring the likely growth patterns in representative tumours, based on genomic and histological evidence (see below).

In the following, we detailed our new analysis on youngest subclones for elucidating possible growth patterns in ccRCCs supported by genomic and histological evidence. Specifically, we focused on the genomic alterations found only in a single region. Whilst sampling bias could confound this analysis and interpretation in some tumours, these genomic alterations could correspond to late events defining recently born subclones in the tumour's evolutionary history. One caveat is that given the available data, we are unable to discriminate between young subclones and older subclones with narrow fitness to outgrow.

Given that late events tend to emerge near the tumour surface in Surface Growth models while throughout the tumour volume in the Volume Growth models (as well as Surface Growth models with necrosis), we examined the spatial localisation of late events, specifically the distance from the region containing the event to the tumour margin (**Figure 5, Supplementary Figure 14**). Interestingly, in a subset of tumours, all such events are found adjacent to the tumour margin, while in others such events can be found in regions either close to or far from the tumour margin. We then focused on mapping single-region events in tumours with at least 10 regions sampled so as to be less confounded by sampling bias. Cases K523, K360, and K234 are examples with all events spanning a single region located within 10 mm from the tumour margin. Intriguingly, relevant tumour regions are among those with highest regional clonal diversity and located behind bulging segments of the tumour contour, both features being characteristic of Surface Growth models. Note that K523 is also the example we highlighted in Figure 5 for the presence of budding structure in both the radiomic image and tumour contour map.
In contrast, cases K165, K272, and K156 are examples with events spanning a single region located at varying distances to the tumour margin. These cases also have high macrodiversity and high ITH index and thus may represent Surface Growth models with necrosis rather than Volume Growth models. In fact, in case K165, several regions harbouring single-region events were located near the bulging segments of the tumour contour, supporting the possibility of Surface Growth pattern. Moreover, in case K156, histological assessment supported that the interior of the section includes

large paucicellular areas, which could reflect available space created by massive cell death in the past and ongoing evolution of fitter clones in the present.

While we are limited by the number of tumours, our preliminary assessment suggested that simulated tumours can capture the diverse growth patterns observed in actual tumours. We will extend these analyses to the complete TRACERx Renal cohort of 324 patients when the data is available.

Changes in the revised manuscript:

1. New model and tumour analyses on spatial features of recent subclones as a step closer towards linking growth modes in computational modelling to ccRCCs.
    a. Results: **Line numbers 5-25 on Page 14, 1-25 on Page 15, 1-2 on Page 16**
    b. Figures: **Figure 4c-h, Supplementary Figures 14-15**
2. New analyses on tumour data in terms of spatial features of clonal diversity connecting Volume Growth models to either indolent or aggressive tumours and Surface Growth models to tumours with attenuated progression
    a. Results: **Line numbers 16-25 on Page 13, 1-3 on Page 14**
    b. **Figures: Figure 3g-h, Supplementary Figure 13**
    c. More detailed discussion in **Supplementary Note 5**

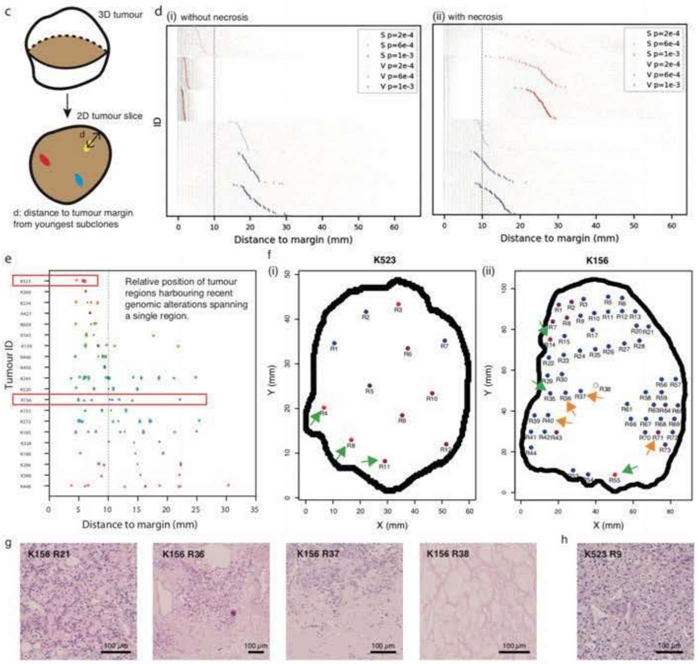

(This figure is presented as **Main Figure 4c-h**)

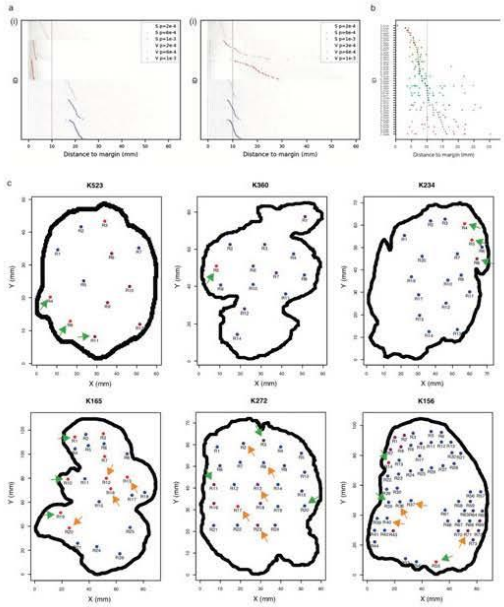

(This figure is presented as **Supplemental Figure 14**)

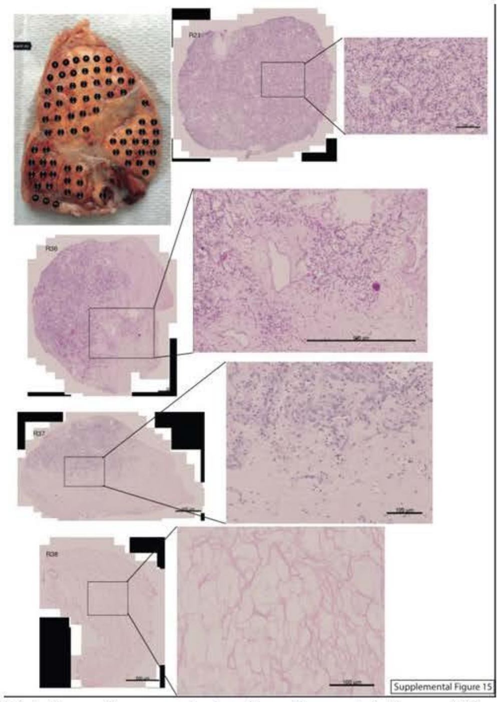

(This figure is presented as **Supplemental Figure 15**)

********************END********************

Decision Letter, second revision:

28th July 2021

Dear Dr. Bates,

Thank you for submitting your revised manuscript "Spatial patterns of tumour growth impact clonal diversification" (NATECOLEVOL-210212914B). It has now been seen again by the original reviewers and their comments are below. The reviewers find that the paper has improved in revision, and therefore we'll be happy in principle to publish it in Nature Ecology & Evolution, pending minor revisions to satisfy the reviewers' final requests and to comply with our editorial and formatting guidelines.

If the current version of your manuscript is in a PDF format, please email us a copy of the file in an editable format (Microsoft Word or LaTex)-- we can not proceed with PDFs at this stage.

We are now performing detailed checks on your paper and will send you a checklist detailing our editorial and formatting requirements in about a week. Please do not upload the final materials and make any revisions until you receive this additional information from us.

Thank you again for your interest in Nature Ecology & Evolution. Please do not hesitate to contact me if you have any questions.

[REDACTED]

Reviewer #1 (Remarks to the Author):

congratulations!

Reviewer #2 (Remarks to the Author):

The authors have responded thoroughly to all my comments and acted on almost all of them. I think that in two cases they can further improve the article by incorporating additional material from their Response.

First, the substantial difference between the growth curves of the surface growth and volume growth models, which the authors describe in detail in their reply, is an important factor in interpreting their findings and therefore needs to be mentioned in the Results. I suggest including the following from the Response (or words to the same effect): "Surface Growth led to polynomial growth with longer time to reach the stopping condition, while Volume Growth resulted in exponential growth," citing a supplementary figure based on Revision Figure 1, and "the faster growth rate in Volume Growth models means a large contribution of parental clone to overall tumour growth and shorter time for advantageous subclones to outgrow and compete, leading to tumours with limited diversification."

Second, the informative discussion of mutation rates on pages 24-25 of the reply should be included

in the Methods or as a supplementary note.

Reviewer #3 (Remarks to the Author):

The authors have comprehensively addressed all points.

Our ref: NATECOLEVOL-210212914B

25th August 2021

Dear Dr. Bates,

Thank you for your patience as we've prepared the guidelines for final submission of your Nature Ecology & Evolution manuscript, "Spatial patterns of tumour growth impact clonal diversification" (NATECOLEVOL-210212914B). Please carefully follow the step-by-step instructions provided in the attached file, and add a response in each row of the table to indicate the changes that you have made. Please also check and comment on any additional marked-up edits we have proposed within the text. Ensuring that each point is addressed will help to ensure that your revised manuscript can be swiftly handed over to our production team.

We would like to start working on your revised paper, with all of the requested files and forms, as soon as possible (preferably within two weeks). Please get in contact with us immediately if you anticipate it taking more than two weeks to submit these revised files.

When you upload your final materials, please include a point-by-point response to any remaining reviewer comments.

If you have not done so already, please alert us to any related manuscripts from your group that are under consideration or in press at other journals, or are being written up for submission to other journals (see: https://www.nature.com/nature-research/editorial-policies/plagiarism#policy-on-duplicate-publication for details).

In recognition of the time and expertise our reviewers provide to Nature Ecology & Evolution's editorial process, we would like to formally acknowledge their contribution to the external peer review of your manuscript entitled "Spatial patterns of tumour growth impact clonal diversification". For those reviewers who give their assent, we will be publishing their names alongside the published article.

Nature Ecology & Evolution offers a Transparent Peer Review option for new original research manuscripts submitted after December 1st, 2019. As part of this initiative, we encourage our authors to support increased transparency into the peer review process by agreeing to have the reviewer

comments, author rebuttal letters, and editorial decision letters published as a Supplementary item. When you submit your final files please clearly state in your cover letter whether or not you would like to participate in this initiative. Please note that failure to state your preference will result in delays in accepting your manuscript for publication.

Cover suggestions

As you prepare your final files we encourage you to consider whether you have any images or illustrations that may be appropriate for use on the cover of Nature Ecology & Evolution.

Covers should be both aesthetically appealing and scientifically relevant, and should be supplied at the best quality available. Due to the prominence of these images, we do not generally select images featuring faces, children, text, graphs, schematic drawings, or collages on our covers.

We accept TIFF, JPEG, PNG or PSD file formats (a layered PSD file would be ideal), and the image should be at least 300ppi resolution (preferably 600-1200 ppi), in CMYK colour mode.

If your image is selected, we may also use it on the journal website as a banner image, and may need to make artistic alterations to fit our journal style.

Please submit your suggestions, clearly labeled, along with your final files. We'll be in touch if more information is needed.

Nature Ecology & Evolution has now transitioned to a unified Rights Collection system which will allow our Author Services team to quickly and easily collect the rights and permissions required to publish your work. Approximately 10 days after your paper is formally accepted, you will receive an email in providing you with a link to complete the grant of rights. If your paper is eligible for Open Access, our Author Services team will also be in touch regarding any additional information that may be required to arrange payment for your article.

Please note that <i>Nature Ecology & Evolution</i> is a Transformative Journal (TJ). Authors may publish their research with us through the traditional subscription access route or make their paper immediately open access through payment of an article-processing charge (APC). Authors will not be required to make a final decision about access to their article until it has been accepted. Find out more about Transformative Journals

<B>Authors may need to take specific actions to achieve compliance with funder and institutional open access mandates. For submissions from January 2021, if your research is supported by a funder that requires immediate open access (e.g. according to Plan S principles) then you should select the gold OA route, and we will direct you to the compliant route where possible. For authors selecting the subscription publication route our standard licensing terms will need to be accepted, including our self-archiving policies. Those standard licensing terms will supersede any other terms that the author or any third party may assert apply to any version of the

manuscript.

Please note that you will not receive your proofs until the publishing agreement has been received through our system.

For information regarding our different publishing models please see our Transformative Journals page. If you have any questions about costs, Open Access requirements, or our legal forms, please contact ASJournals@springernature.com.

Please use the following link for uploading these materials:
[REDACTED]

If you have any further questions, please feel free to contact me.

[REDACTED]

Reviewer #1:
Remarks to the Author:
congratulations!

Reviewer #2:
Remarks to the Author:
The authors have responded thoroughly to all my comments and acted on almost all of them. I think that in two cases they can further improve the article by incorporating additional material from their Response.

First, the substantial difference between the growth curves of the surface growth and volume growth models, which the authors describe in detail in their reply, is an important factor in interpreting their findings and therefore needs to be mentioned in the Results. I suggest including the following from the Response (or words to the same effect): “Surface Growth led to polynomial growth with longer time to reach the stopping condition, while Volume Growth resulted in exponential growth,” citing a supplementary figure based on Revision Figure 1, and “the faster growth rate in Volume Growth models means a large contribution of parental clone to overall tumour growth and shorter time for advantageous subclones to outgrow and compete, leading to tumours with limited diversification.”

Second, the informative discussion of mutation rates on pages 24-25 of the reply should be included in the Methods or as a supplementary note.

Reviewer #3:

naturereresearch

Remarks to the Author:
The authors have comprehensively addressed all points.

Final Decision Letter:

7th October 2021

Dear Dr Bates,

We are pleased to inform you that your Article entitled "Spatial patterns of tumour growth impact clonal diversification", has now been accepted for publication in Nature Ecology & Evolution.

Before your manuscript is typeset, we will edit the text to ensure it conforms to house style.

Once your manuscript is typeset you will receive a link to your electronic proof via email, with a request to make any corrections as soon as possible. If you have queries at any point during the production process then please contact the production team at rjsproduction@springernature.com. Once your paper has been scheduled for online publication, the Nature press office will be in touch to confirm the details.

Acceptance of your manuscript is conditional on all authors' agreement with our publication policies (see www.nature.com/authors/policies/index.html). In particular your manuscript must not be published elsewhere and there must be no announcement of the work to any media outlet until the publication date (the day on which it is uploaded onto our web site).

Please note that <i>Nature Ecology & Evolution</i> is a Transformative Journal (TJ). Authors may publish their research with us through the traditional subscription access route or make their paper immediately open access through payment of an article-processing charge (APC). Authors will not be required to make a final decision about access to their article until it has been accepted. Find out more about Transformative Journals

<B>Authors may need to take specific actions to achieve compliance with funder and institutional open access mandates. For submissions from January 2021, if your research is supported by a funder that requires immediate open access (e.g. according to Plan S principles) then you should select the gold OA route, and we will direct you to the compliant route where possible. For authors selecting the subscription publication route our standard licensing terms will need to be accepted, including our self-archiving policies. Those standard licensing terms will supersede any other terms that the author or any third party may assert apply to any version of the manuscript.

In approximately 10 business days you will receive an email with a link to choose the appropriate publishing options for your paper and our Author Services team will be in touch regarding any additional information that may be required.

naturereseach

You will not receive your proofs until the publishing agreement has been received through our system.

If you have any questions about our publishing options, costs, Open Access requirements, or our legal forms, please contact ASJournals@springernature.com

An online order form for reprints of your paper is available at https://www.nature.com/reprints/author-reprints.html. All co-authors, authors' institutions and authors' funding agencies can order reprints using the form appropriate to their geographical region.

We welcome the submission of potential cover material (including a short caption of around 40 words) related to your manuscript; suggestions should be sent to Nature Ecology & Evolution as electronic files (the image should be 300 dpi at 210 x 297 mm in either TIFF or JPEG format). Please note that such pictures should be selected more for their aesthetic appeal than for their scientific content, and that colour images work better than black and white or grayscale images. Please do not try to design a cover with the Nature Ecology & Evolution logo etc., and please do not submit composites of images related to your work. I am sure you will understand that we cannot make any promise as to whether any of your suggestions might be selected for the cover of the journal.

You can now use a single sign-on for all your accounts, view the status of all your manuscript submissions and reviews, access usage statistics for your published articles and download a record of your refereeing activity for the Nature journals.

To assist our authors in disseminating their research to the broader community, our SharedIt initiative provides you with a unique shareable link that will allow anyone (with or without a subscription) to read the published article. Recipients of the link with a subscription will also be able to download and print the PDF.

You can generate the link yourself when you receive your article DOI by entering it here: <a href="http://authors.springernature.com/share">http://authors.springernature.com/share<a>.

[REDACTED]

P.S. Click on the following link if you would like to recommend Nature Ecology & Evolution to your librarian http://www.nature.com/subscriptions/recommend.html#forms

** Visit the Springer Nature Editorial and Publishing website at www.springernature.com/editorial-and-publishing-jobs for more information about our career opportunities. If you have any questions please click here.**
